# Habitat fragmentation and species diversity in competitive communities

**DOI:** 10.1111/ele.13450

**Published:** 2019-12-20

**Authors:** Joel Rybicki, Nerea Abrego, Otso Ovaskainen

**Affiliations:** ^1^ Institute of Science and Technology Austria (IST Austria) Am Campus 1 3400 Klosterneuburg Austria; ^2^ Department of Agricultural Sciences University of Helsinki PO Box 27 FI‐00014 Helsinki Finland; ^3^ Oragnismal and Evolutionary Biology Research Programme University of Helsinki PO Box 65 FI‐00014 Helsinki Finland

**Keywords:** Fragmentation, habitat loss, individual‐based models, metacommunity theory, simulation model, spatial models, species richness

## Abstract

Habitat loss is one of the key drivers of the ongoing decline of biodiversity. However, ecologists still argue about how fragmentation of habitat (independent of habitat loss) affects species richness. The recently proposed habitat amount hypothesis posits that species richness only depends on the total amount of habitat in a local landscape. In contrast, empirical studies report contrasting patterns: some find positive and others negative effects of fragmentation *per se* on species richness. To explain this apparent disparity, we devise a stochastic, spatially explicit model of competitive species communities in heterogeneous habitats. The model shows that habitat loss and fragmentation have complex effects on species diversity in competitive communities. When the total amount of habitat is large, fragmentation *per se* tends to increase species diversity, but if the total amount of habitat is small, the situation is reversed: fragmentation *per se* decreases species diversity.

## Graphical abstract

Habitat loss is one of the key drivers of the ongoing decline of biodiversity, but ecologists still debate how fragmentation of habitat (independent of habitat loss) affects species richness. We devise a model of competitive species communities, which shows that habitat loss and fragmentation have complex effects on species diversity. When the total amount of habitat is large, fragmentation *per se* tends to increase species diversity, but if the total amount of habitat is small, the situation is reversed: fragmentation *per se* decreases species diversity

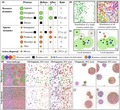

## Introduction

Degradation and loss of natural habitat due to anthropogenic modification and climate change is a key factor contributing to the ongoing sixth extinction event (Tilman *et al*. 1994; Fahrig 2003; Thomas *et al*. 2004; Kuussaari *et al*. 2009; Pereira *et al*. 2010; Butchart *et al*. 2010; Pimm *et al*. 2014). Habitat *loss* (reduction in area with suitable habitat) typically goes hand in hand with habitat *fragmentation* (division of the habitat into several parts), where the former is a process causing the latter landscape pattern (Fahrig 2003; Ewers & Didham 2005; Wilson *et al*. 2016). While the decline of biodiversity due to habitat loss is uncontested, the effect of habitat fragmentation *per se* on species richness has been much debated over the past decades (Fahrig 2003; Ewers & Didham 2005; Didham *et al*. 2012; Fahrig 2013; Hanski 2015; Haddad *et al*. 2015; Fahrig 2017; Fletcher *et al*. 2018; Fahrig *et al*. 2019): given the same total amount of habitat, how does the spatial configuration of the habitat, that is, the locations, shapes and sizes of habitat fragments, influence biodiversity?

Indeed, one of the key questions in conservation biology is whether protecting biodiversity is better achieved using a single large or several small (SLOSS) reserves (Diamond 1975; Ewers & Didham 2005). For species that follow classical metapopulation dynamics (Hanski 1999), the effects of fragmentation and spatial configuration are well‐understood from the theoretical perspective (Bascompte & Solé 1996; Hanski & Ovaskainen 2000; Ovaskainen 2002; Hanski & Ovaskainen 2003; Gilarranz & Bascompte 2012; Grilli *et al*. 2015): single species metapopulation theory predicts that increasing fragmentation is detrimental for species – although the response is not necessarily monotone (Ovaskainen 2002) – assuming that no evolutionary responses take place; see Legrand *et al*. (2017) for a review of ecoevolutionary responses to habitat fragmentation. In contrast, increasing connectivity in fragmented landscapes can increase synchrony in metapopulations, and consequently, lead to increased extinction risk (Kahilainen *et al*. 2018).

While there is a fairly good understanding of single‐species metapopulation dynamics, not all species necessarily follow metapopulation dynamics or the scale at which they do is limited. Moreover, the situation becomes much more muddled when considering species communities that comprise several interacting species. While increasing fragmentation is known to largely have negative effects for metapopulations, this does not necessarily hold for metacommunities of several species. Indeed, while some species (e.g. habitat specialists) may suffer from fragmentation, others may benefit from it (e.g. generalists and edge species) (Henle *et al*. 2004). When considering community‐level properties, such as species richness, both theoretical and empirical studies have observed that different spatial configurations of the habitat can have both negative and positive effects on species richness (Tilman *et al*. 1997; Rybicki & Hanski 2013; Hanski *et al*. 2013; Hanski 2015; Haddad *et al*. 2015; Fahrig 2017; Thompson *et al*. 2017; Haddad *et al*. 2017; Loke *et al*. 2019) depending on the species' traits together with structure of the habitat (e.g. degree of spatial autocorrelation in habitat types). Nevertheless, current theory still suggests that fragmentation (*per se*) tends to increase extinctions in predator‐prey metapopulations and competitive metacommunities (Tilman *et al*. 1997).

In a recent meta‐analysis of empirical fragmentation studies, Fahrig (2017) concluded that the most significant ecological responses to habitat fragmentation were positive; see also Fletcher *et al*. (2018) for a critique of this meta‐analysis and a response by Fahrig *et al*. (2019). The positive effects of fragmentation have been attributed to numerous causes including – but not limited to – increase in functional connectivity, diversity of habitat types, persistence of predator–prey systems and decrease in intra‐ and interspecific competition.

In contrast, other studies have reported negative effects including increased risk of extinction due to reduced genetic diversity, and increased environmental and demographic stochasticity in small patches (Ewers & Didham 2005). Various edge effects have been proposed to have both positive and negative effects (Ewers & Didham 2005; Fahrig 2017) depending on the species traits. Furthermore, fragmentation may alter species interactions and community composition, as invasive or pest species may replace the original species pool, increase the transmission and prevalence of disease in small fragments, and the effects of fragmentation can be confounded by the associated time lags (Ewers & Didham 2005; Haddad *et al*. 2015). Indeed, long‐term experiments suggest that full responses to altered habitat configuration and connectivity unfold over extended periods of time (Damschen *et al*. 2019).

### The habitat amount hypothesis

To make sense of the effects of fragmentation on species richness, Fahrig (2013) has proposed the *habitat amount hypothesis*, which postulates that species richness is best explained by the sample area effect: large areas of habitat tend to support more individuals, and hence, more species (Rosenzweig 1995). More specifically, the hypothesis posits that what truly matters is the *total amount* of habitat in an *appropriate spatial extent of the local landscape* independent of its spatial configuration (Fahrig 2013). Namely, when examining the number of species in sample sites placed within habitat, and considering the local landscapes surrounding each sample site, the hypothesis makes the following predictions (see Fahrig 2013 for a detailed exposition):

- Prediction 1: Given equal‐sized sample sites, species richness increases with total amount of habitat in the local landscape surrounding the sample site.
- Prediction 2: Species richness in a sample site only depends on the total amount of habitat in the surrounding local landscape. That is, it is independent of the area of the particular habitat fragment in which the site is located, except to the extent of habitat area the fragment itself contributes to the surrounding landscape.

Fahrig (2013, 2015) has called for a research programme to test the hypothesis, and subsequently, the hypothesis has recently received considerable attention and several empirical ecologists have tested its validity; yet they have reported disparate results. While Melo *et al*. (2017) found that the habitat amount hypothesis holds for South American small mammals, Haddad *et al*. (2017) found that the hypothesis does not hold for plant and micro‐arthropod communities. Furthermore, Arnillas *et al*. (2017) reported that fragmentation may have positive effect on species richness – at least on the short‐term – and De Camargo *et al*. (2018) found that for birds there is no detectable response to fragmentation at the landscape level, but did not rule out the possibility that fragmentation matters at smaller scalers.

The habitat amount hypothesis has been criticised for lacking an underlying mechanistic explanation of how species interactions and community dynamics affect species richness, and which would predict the appropriate spatial scale at which the hypothesis holds (Hanski 2015; Fletcher *et al*. 2018; but see Jackson & Fahrig 2012; Fahrig 2013). Recent empirical results (MacDonald *et al*. 2018; Vieira *et al*. 2018) suggest that response of species diversity to habitat loss and fragmentation is best explained by connecting the habitat amount hypothesis to the theory of island biogeography (MacArthur & Wilson 1967) and habitat diversity hypothesis (Williams 1964). Indeed, it is important to take into account the scale of the landscape, as the relative importance of habitat diversity and amount varies with island or fragment area (Lomolino & Weiser 2001; Sfenthourakis & Triantis 2009), which may explain why the habitat amount hypothesis remains so controversial.

However, it still remains unclear which species and landscape attributes lead to different responses to habitat fragmentation (Fahrig 2017), and there is no unifying theoretical framework explaining the impact of habitat loss and fragmentation on biodiversity. As pointed out by Fletcher *et al*. (2018), there is an urgent need for mechanistic models that help to discern the separate effects of habitat loss and fragmentation.

### Contributions

In this work, we set out to make better understanding of the effects of fragmentation and habitat loss using a mechanistic simulation model. We develop a novel individual‐based, spatially explicit model of competitive species communities in spatiotemporally varying landscapes. Our model relaxes many assumptions made in prior metacommunity models; see Appendix A for a short overview of prior work. For example, we do *not* assume that the species follow metapopulation dynamics or that the habitat consists of discrete patches. Instead, we keep track of individuals who follow stochastic continuous‐time birth‐death dynamics in continuous spatiotemporally varying landscapes.

Using our model, we examine how habitat fragmentation *per se* influences species richness in competitive species communities. Our results show that fragmentation can have *positive* effects on species richness of competitive habitat specialists if the total amount of habitat in a landscape is large. However, when the total amount of habitat is small, high fragmentation yields *negative* effects. In general, we see that response to fragmentation is not necessarily monotone: species richness may increase at small and intermediate levels of fragmentation, but decrease at high levels of fragmentation. Finally, we observe that fragmentation has the same *qualitative* effect on both species that are sessile after dispersal and non‐sessile species that actively move in the landscape in order to find suitable habitat.

We use our simulation model to test the habitat amount hypothesis as proposed by Fahrig (2013). In some scenarios, we obtain results compatible with the hypothesis, whereas in others not. In particular, we see that different analyses of fragmentation effects applied to the same data may lead to apparently contradictory results. This suggests that much caution is needed when interpreting whether empirical data shows that fragmentation has positive or negative effects on species richness of habitat specialists.

## Model and Methods

### Overview of the individual‐based spatial model

We devised an individual‐based community model in continuous time and continuous two‐dimensional, spatiotemporally heterogeneous landscapes, where the survival and reproduction of all individuals is governed by a limiting resource. Our model contains three basic entity types: resource patches, resource particles, and individuals of (each) species. The resource patches produce resource particles into their neighbourhood, and the individuals of the species consume these particles. Thus, we obtain intra‐ and interspecific resource competition yielding density‐dependent population growth. The species individuals convert the resources into offspring and all individuals follow birth–death dynamics.

**Figure 1:**
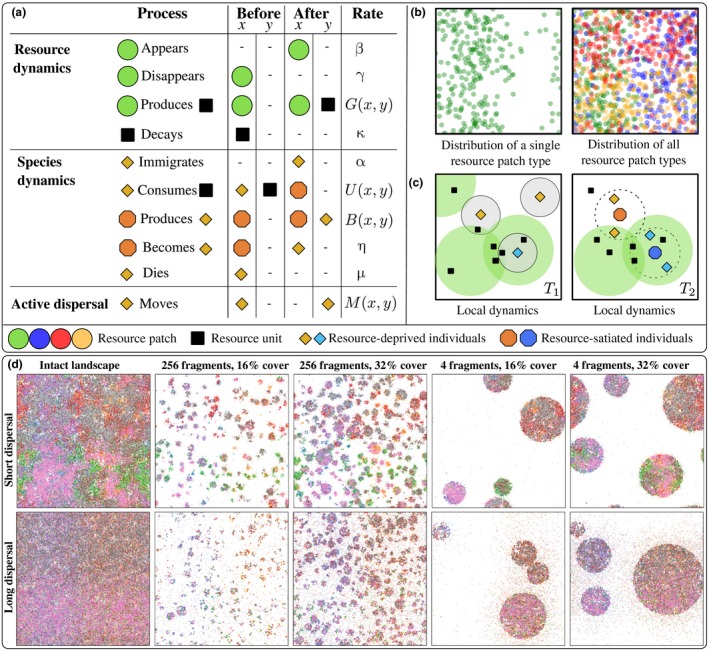
(a). Overview of the model. Rows represent processes which turn entities in positions *x* and *y* (‘before’ column) into a new configuration (‘after’ column) at given rates (last column). The Greek letters are positive constants, *G* and *U* are top‐hat kernels, and *B* and *M* are Gaussian kernels (see SI text for details). (b) Illustration of the large‐scale environmental variation in habitat types. The panels show a snapshot of the sinusoidal resource patch distributions in an intact 100 × 100 continuous landscape. (c) Example cartoon of local dynamics over time in a continuous 4 × 4 area contained in a larger landscape. *T*_1_: Resource‐deprived individuals immigrate into the area. They consume resources that are within their utilisation radius (gray area). *T*_2_: some individuals have become resource‐satiated by consuming resource units, and have produced offspring into their surroundings within distance δ (dotted circles). (d) Snapshots of simulations with eight species in landscapes with varying degree of fragmentation and habitat cover in a continuous 100 × 100 landscape. The top row shows a scenario with δ = 1 and bottom row a scenario with δ = 10, both with passive dispersal. Coloured dots represent species individuals (larger ones are resource‐satiated and smaller are resource‐deprived individuals, respectively). The resource patches, resource units and habitat fragments are not drawn. In both cases the matrix is habitable.

Formally, our model is a spatiotemporal point process, or in the mathematical terminology, a Markov evolution in the space of locally finite configurations (see e.g. Ovaskainen *et al*. 2014; Cornell *et al*. 2019): The dynamics of the model can be described by listing all events that can take place and the rates at which these events occur. These rates can depend on the current spatial configuration of all individuals (e.g. individuals can only consume resources that are within their proximity). Fig. 1a gives an overview of the model; the full description appears in Appendix B.

#### Resource and species dynamics

A species' individual can be either *resource‐satiated* or *resource‐deprived*. Resource‐deprived individuals become satiated when consuming resources. Satiated individuals produce new individuals who start in the resource‐deprived state. Resource‐satiated individuals become resource‐deprived at constant per capita rate, and resource‐deprived individuals die at a constant per capita rate.

#### Large‐scale environmental variation in habitat types

We assume that the species pool is divided into four equal‐sized groups, where each group is specialised on a distinct resource type. Besides this difference, each species shared identical parameters (summarised in Table S1 in Appendix B). Each resource type had spatial variation in the form of a nonlinear, sinusoidal environmental gradient along one axis (Fig. 1b) so that the total resource production rate for all resource types was equal in an intact landscape (see Appendix B). In Appendix C, we investigate the completely neutral setting with a single limiting resource shared by all species.

#### Dispersal and immigration

We consider two modes of dispersal for the species:

- *passive* (one‐shot) dispersal, where individuals do not move during their life time,
- *active* dispersal, where resource‐deprived individuals move to find suitable habitat.

In both cases, the range of dispersal is controlled by a scale parameter δ. We model immigration of individuals from outside the focal landscape by assuming that resource‐deprived individuals appear at a (small) constant per unit area rate of α = 10^−3^ (see Table S1).

#### Fragmentation

We examine finite two‐dimensional landscapes. To avoid boundary effects, we assume the focal landscape to be a two‐dimensional torus of size *V* ×* V*. To model habitat loss and fragmentation, we consider scenarios where the focal landscape is partitioned into *N* disjoint (non‐overlapping) circular *habitat fragments*, where the fragment *i* is centred at location *x_i_* and consists of all points within radius *r_i_*. The area covered by habitat fragments is said to be *habitat*, whereas the *matrix* M then consists of the points that are not part of any habitat fragment. Note that *resource patch* and *habitat fragment* are distinct notions in our model.

We consider two contrasting scenarios on how fragmentation influences the community dynamics:

- *Habitable matrix*: Fragmentation only influences resource production, but the species themselves are not (directly) affected by the matrix in any way. More precisely, resource particles (produced by resource patches) can only establish within habitat fragments so that a resource particle may only appear to location *y* if y∉M. That is, the resource production rate in the matrix is always zero. The fragment and matrix have no other direct effects.
- *Hostile matrix*: Fragmentation influences directly both the resource production and the species survival. In addition to the above constraint on resource production, we assume that the species individuals cannot survive in the matrix. That is, any resource‐deprived individual that immigrates or disperses into location y∉M in the matrix is immediately killed. As satiated individuals do not move, it follows that satiated individuals can only reside within fragments.

The first scenario makes minimal assumptions on effects of fragmentation, as it only affects how resource particles are generated. In particular, the scenario allows species to survive in the matrix given close enough proximity to habitat fragments, whereas the second scenario explicitly prevents this.

### Simulation experiments

We simulated the model in continuous landscapes of size 100 × 100 with a pool of *S* = 128 species. To generate different types of species communities, we considered three scales of dispersal (from short δ = 1, to intermediate δ = 3 and long‐range δ = 10), two modes of dispersal (passive and active) and two matrix types (habitable and hostile). For the sake of simplicity, we excluded the scenario with active dispersal and hostile matrix, as any individual moving outside the habitat fragment would immediately die in a hostile matrix, thus limiting the potential benefit of active dispersal under fragmentation. In total, we obtained nine different types of species communities.

We constructed landscapes with varying number *N* = 4*^k^* of habitat fragments, where 0 ≤ *k* *≤* 5, and fraction *C* of total area covered by the fragments with *C* = 0.48 or *C* = 2*^a^*/100, where −3 *≤* *a* *≤* 5 is an integer; see Fig. 1d and Fig. S1 for examples of the generated landscapes. For each combination of *N* and *C*, we generated *R* = 100 random replicate landscapes yielding a total of 6 × 10 × 100 = 6000 landscapes. Each replicate was simulated for *T* = 400 time units, as by this time the system had converged close to the stationary state (Fig. S3). In each landscape, we collected data of the total number of *resource‐satiated* individuals (of each species) in

1. each individual habitat fragment,
2. the entire focal landscape and
3. fixed‐size sampling windows of radius τ = 1 centred in each fragment with radius of at least τ (so that each sample site intersects with only one habitat fragment).

From data (1), we carried out the SLOSS analysis (Fahrig 2013), data from (2) were used to plot species–fragmented area relationship (SFAR) curves (Rybicki & Hanski 2013) and data from (3) was used to test the sample area effect.

#### SLOSS analysis

For the SLOSS curves, we sorted the habitat fragments in both increasing and decreasing order and then plotted the cumulative number of species against the cumulative habitat amount. We then took the average of the cumulative species number *S_k_* and fraction of total habitat amount *A_k_* overall replicates, where

$$A_{k}=\sum_{i=1}^{k}{\bar{A}}_{i}\;\text{and}\;S_{k}=\sum_{i=1}^{k}{\bar{S}}_{i}$$

and ${\bar{A}}_{i}$ and ${\bar{S}}_{i}$ denote the average area and species count of the *i*th fragment in the given sorted order (either decreasing or increasing in area).

#### SFAR curves

For the SFAR curves, we plotted the average number of species *S* present in each simulated landscape. For every landscape with a total habitat cover of *C* and *N* fragments, we calculated the average number of species present in the entire landscape.

#### Testing the sample area effect

From data (3), we examined how well the amount of habitat (i.e. area covered by habitat fragments) in the surrounding *local* landscape explains the number of species at a fixed‐size sample site. First, to obtain independent data points, we sampled from each replicate landscape a single sampling site (completely contained within a habitat fragment) uniformly at random.

Then for all sampling sites obtained from the independent replicate landscapes, we examined the radius‐*r* local landscapes centred on the sample sites for increasing values of *r*. For each radius‐*r* local landscape, we approximated the amount of habitat cover within the radius‐*r* ball using a rejection sampling method. We then tested at which scale the amount of habitat in the *r*‐radius local landscape best predicted the number of species at a sample site. This was done by fitting a Poisson regression model, where the response variable was the number of species in a fixed‐size sample site and the explanatory variable was the *log‐transformed* amount of habitat in the *r*‐radius neighbourhood surrounding the sample site. Then for each value of *r*, we calculated the pseudo‐*R* ^2^ value 1 – *D/D*_null_, where *D* was the deviance and *D*_null_ the null deviance of the fitted Poisson regression model (with the intercept‐only model being the null model). We then identified the appropriate radius of the local landscape by choosing the value of *r* that maximised the pseudo‐*R* ^2^ value.

The habitat amount hypothesis predicts that (Prediction 1) species richness in a give sample site increases with the amount of habitat in the local landscape, and that (Prediction 2) if the amount of habitat in the local landscape remains constant, species richness should be independent of the size of the habitat fragment containing the sample size (Fahrig 2013, fig. 7). To test these predictions, we fitted to these data Poisson regression models where the explanatory variables were log‐transformed habitat amount *L* in the local landscape and log‐transformed area *F* of the focal fragment containing the sample site. We fitted four models which contained either both of the candidate explanatory variables *L* and *F*, either one of them alone, or neither of them. We compared the models with ∆AIC, normalised to zero for the best supported model. Prediction 1 is supported if the model containing habitat amount in the local landscape performs better than the null model and has a positive slope. Prediction 2 is not supported if fragment area has an additional positive effect on top of the effect of local habitat amount.

## Results

### The SLOSS analysis

**Figure 2:**
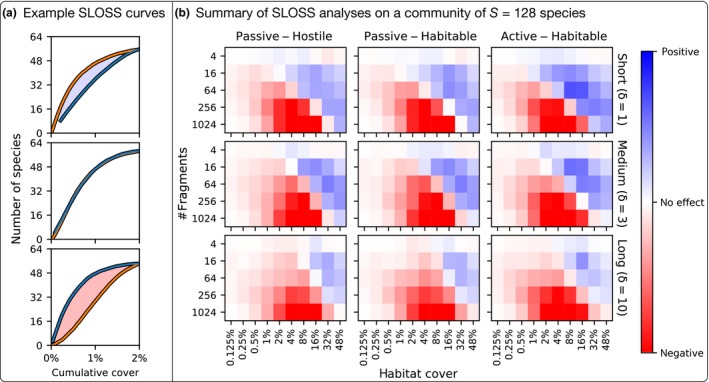
Summary of the SLOSS analysis. (a) Example SLOSS analysis curves from an analysis on a community with *S* = 64 species. The habitat fragments have been sorted in increasing (from smallest patch to largest, orange) and decreasing (largest patch to the smallest, blue) order. The horizontal axis indicates the *cumulative* habitat cover and the vertical axis the *cumulative* number of species averaged over all replicates. When the orange line is above the blue line, fragmentation has a positive effect on species richness (top box), whereas when the blue line is above the orange line, fragmentation has a negative effect on species richness (bottom box). If both lines overlap (middle box), then fragmentation *per se* has no effect (as predicted by the habitat amount hypothesis). (b) SLOSS analysis on the simulated data with *S* = 128 species in a 100 × 100 landscape. For each value of δ and dispersal mode, there is a coloured grid that summarises the SLOSS analysis for 5 × 10 landscape scenarios. In each grid, the vertical axis gives the total number of fragments in the landscape and the horizontal axis the total habitat cover. The colour of the cell indicates the value *I*/*C*, where *I* is the integral between the orange and blue lines normalised by the maximum cover *C*. Blue indicates positive effect of fragmentation (orange line above blue line, top box) and red indicates negative effect (blue above orange line, bottom box). Thus, stronger the colour, more pronounced the effect of fragmentation.

Fahrig (2013) suggested conducting SLOSS analyses as an indirect test for whether the mechanisms underlying species–area relationship is due to the sample area effect, island effect or possibly some other mechanisms. As discussed, there are two SLOSS curves: (1) the fragments are sorted in both increasing and (2) decreasing order of area, and then the cumulative number of species is plotted against the cumulative habitat amount; see Fig. 2a for examples. In our case, as the SLOSS analysis needs to be done for each landscape separately, this yields thousands of plots for each of the nine community scenarios which we have considered. The result of all these analyses is summarised in Fig. 2b.

The relationship between the two curves can be interpreted as follows (see e.g. fig. 5 of Fahrig 2013 and fig. 7 of Fahrig 2017):

- Positive fragmentation effect (blue values): Cumulative number of species increases faster when considering fragments in increasing order (top box Fig. 2a). That is, given the same cumulative habitat area, several small patches contain more species than few large patches.
- No fragmentation effect: The curves overlap so that the cumulative number of species is the same irrespective of the number of fragments making up the total habitat (centre box Fig. 2a). In this case, the species–area relationship is driven by the sample area effect, which is consistent with the habitat amount hypothesis.
- Negative fragmentation effect (red values): There are more species in a single large fragment than in several small fragments of the same total area (bottom box Fig. 2b). The species‐area relationship is driven by the ‘island effect’.

Thus, positive values (blue) in Fig. 2b suggest that the size of the fragments affects species richness (at fragment‐level) less than expected from sampling area effect, whereas negative values mean that the sample area effect alone is insufficient to explain the species‐area relationship, that is, there is an island effect (Fahrig 2013). From Fig. 2b, we see evidence for the sample area effect only at very limited ranges. In particular, we see that when the dispersal distances are short or intermediate, fragmentation shows both positive and negative effects. This depends on the total amount of habitat available: at low habitat cover, fragmentation is detrimental, whereas fragmentation in landscapes with high habitat cover has a positive effect.

### Species–fragmented area relationships

**Figure 3:**
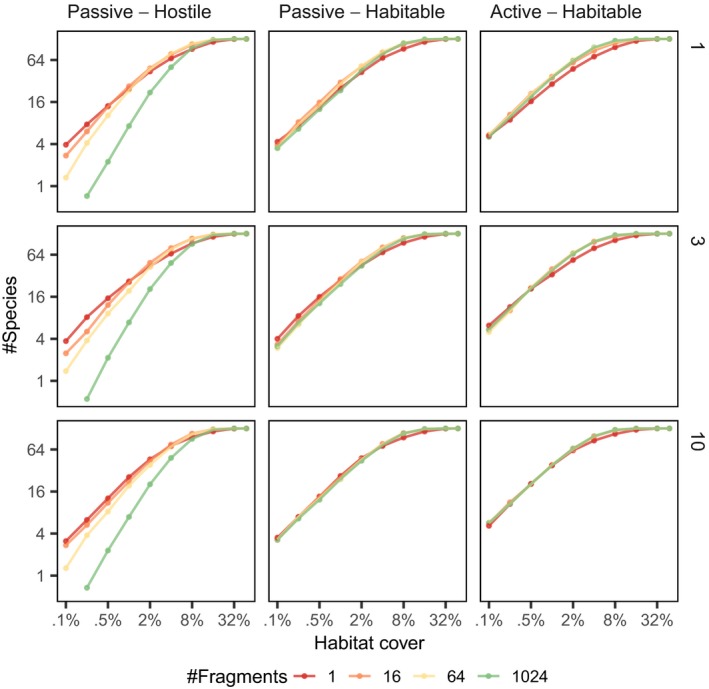
Species–fragmented area curves. The plot shows the average number of species (vertical axis) over in a landscape of given total habitat cover (horizontal axis) and number of fragments (averaged over all replicates). Both axes have logarithmic scale. Each row of panels represents results for different dispersal range and each column for different dispersal/matrix scenario.

The SLOSS curves only account for species observed within habitat fragments, but the species–area relationship curves (Fig. 3) show the species diversity at the metacommunity level that is, how many species were present in the entire landscape. Note that even if there is a single resource‐satiated individual in the landscape, then the species is counted to be present (no matter how unlikely sampling it might be).

The species–area relationship exhibits different patterns depending on whether the matrix is habitable or hostile for the focal species. If the matrix is hostile, that is the individuals cannot move and survive the matrix, then the SFAR plots show higher levels of fragmentation being detrimental for metacommunity diversity: increasing fragmentation but keeping total amount of habitat constant (first column of Fig. 3) lowers species richness at the metacommunity level.

However, when the matrix is habitable, that is individuals can move and survive in the matrix (but the resources still have to be obtained from habitat fragments), SFAR plots shows that fragmentation increases species richness if the dispersal distances are short (last column in Fig. 3). When the dispersal distances are large compared to the size of the entire landscape, the SFAR suggests that the metacommunity species richness depends mostly only on the total amount of habitat.

**Figure 4:**
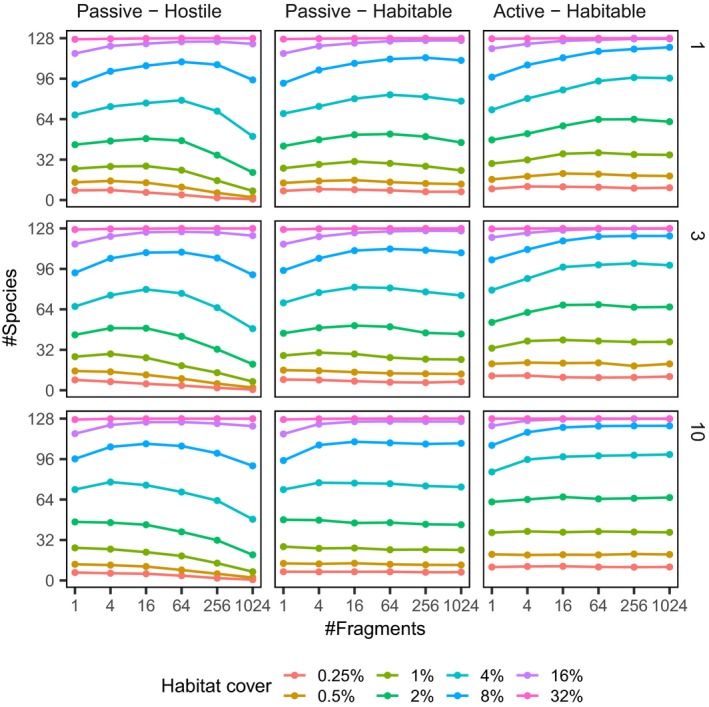
Species richness as a function of habitat fragmentation for the different scenarios. Each point represents the average species richness in landscapes with given number of habitat fragments and amount of total habitat. The vertical axis gives average species richness in landscapes, whereas the horizontal axis represents the degree of fragmentation (in logarithmic scale, fragmentation increases from left to right) and colours denote the total amount of habitat cover. The columns represent different dispersal/matrix scenarios and rows represent different dispersal ranges.

To see better how total species richness at the landscape level responds to fragmentation *per se*, Fig. 4 plots species richness as a function of the number of habitat fragments when total habitat cover is kept fixed. At high amounts of habitat (≥32%), the level of fragmentation has little to no effect, but as the total amount of habitat decreases, fragmentation starts to show effects on species diversity. At short dispersal distances (top row), fragmentation can increase species richness under the habitable matrix scenarios (last two columns), but in the hostile matrix scenario, high levels of fragmentation are detrimental for species richness. When dispersal distances are large (bottom row), fragmentation has little effect in scenarios where the matrix is habitable and a negative effect when the species cannot survive in the matrix (first column).

### Testing the sample area effect

**Figure 5:**
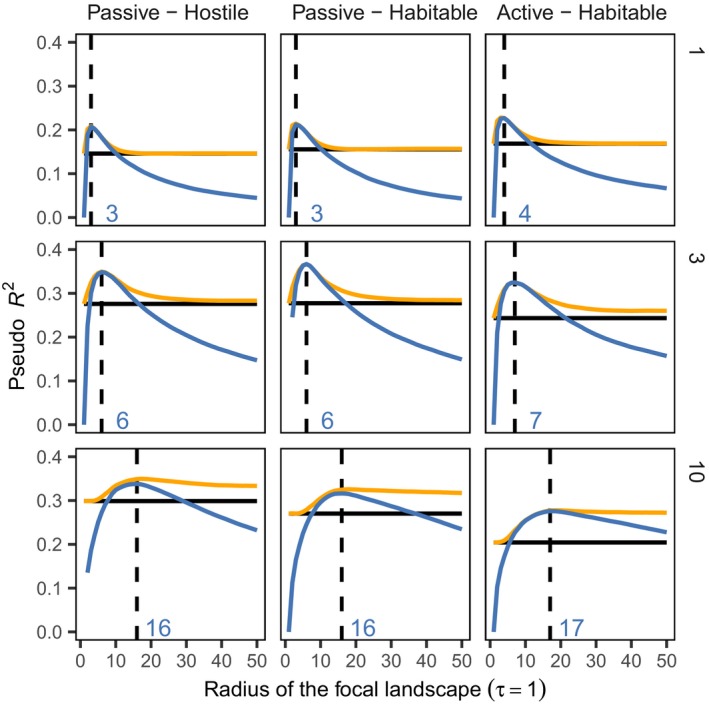
Inferring the appropriate scale of local landscape and testing the sample area effect. The rows correspond to different dispersal scales δ = 1, 3, 10 and columns different dispersal/matrix scenarios. We considered three types of predictors of species richness: the amount of habitat in the local landscape *A_r_* (blue line), the amount of habitat together with the size of the focal fragment (orange line) and area *F* of the focal fragment alone (horizontal black line). The horizontal axis denotes the radius of the local landscape (in the first two models) and the vertical axis the goodness of fit of the model (pseudo‐*R* ^2^). The vertical dashed line and blue number give the scale at which the amount of habitat in the local landscape best explains species richness (i.e. blue line attains its maximum).

For testing the sample area effect, we examined the number of species in fixed‐size sample sites, as a function of the area of the *local* landscape. The scale at which the amount of habitat in the surrounding landscape best explained species richness (highest pseudo‐*R* ^2^ value) was chosen as the appropriate scale of the local landscape. The blue lines in Fig. 5 give the goodness‐of‐fit of for different radii of local landscape in the nine different scenarios and the vertical dashed line shows the inferred scale of the local landscape. For comparison, the horizontal dashed line shows how well the area of the focal fragment (in which the sample site resides) explains species richness. The orange line corresponds to the model with both the amount of habitat in the local landscape and the total area of the focal fragment as predictors. The exact goodness‐of‐fit measure (e.g. deviance) did not affect the qualitative results of the analysis.

When the appropriate scale of the local landscape is correctly chosen, the total amount of habitat in the local landscape explains species richness better than size of the focal fragment alone (blue line above the horizontal line). However, if the dispersal distance is short (top row), the explanatory power of total habitat amount in a local landscape is sensitive to the correct choice of scale for the local landscape. As dispersal distances increase (lower rows), the less sensitive the models become to the choice of the scale.

When considering the two predictions of the habitat amount hypothesis, the results (Fig. 5; Table 1) provide full support for Prediction 1: the model with local habitat amount as the only explanatory variable had always a positive slope and was much better supported than the null model. Prediction 2 was supported when dispersal is long (δ = 10), as in these cases, the fragment area did not improve the model that already contained local habitat amount or its effect was negative rather than positive. For cases with short dispersal, fragment area provided a small additional positive contribution to species richness, even if the size of the local landscape was optimised, but the influence of fragment area was much smaller than the local habitat amount, giving partial support to Prediction 2. However, we observe that Prediction 2 is sensitive to correctly identifying the appropriate scale for the local landscape, as for non‐optimal radii for the local landscape, support for Prediction 2 vanishes (orange line goes above blue line in Fig. 5).

**Table 1:** Testing the sample area effect for two models

	δ	β*_L_* (*M_L_*_+_ *_F_*)	β*_F_* (*M_L_*_+_ *_F_*)	β*_L_* (*M_L_*)	β*_F_* (*M_F_*)	AIC *M_L_*_+_ *_F_*	AIC *M_L_*	AIC *M_F_*	AIC *M*_null_
H	1	0.43	0.02	0.48	0.13	0	4.18	334.85	1116.4
H	3	0.43	0.02	0.48	0.21	0	5.73	569.25	2712.16
H	10	0.29	0.08	0.4	0.25	0	86.46	404.52	2819.03
P	1	0.41	0.03	0.47	0.13	0	9.78	301.38	1101.75
P	3	0.48	0	0.48	0.21	1.9	0	701.21	2872.84
P	10	0.29	0.07	0.38	0.24	0	64.7	423.59	2534.52
A	1	0.34	0.01	0.36	0.13	0	0.07	330.02	1283.33
A	3	0.37	−0.01	0.36	0.17	1.25	0	626.2	2496.63
A	10	0.26	0.02	0.29	0.17	0	8.53	565.51	2155.28

The row labels H, P and A denote the three modes (passive/hostile, passive/habitable and active/habitable) and δ denotes the average dispersal distance. Here, *L* denotes the log‐transformed total amount of habitat in the local landscape and *F* the log‐transformed area of the fragment. There are three regression models that explain the number of species given *L* and/or *F*. The columns β*_x_*(*M*) give the coefficient for explanatory variable *x* in model *M*. The last four columns give the ∆AIC values for each model and the null model.

### Sensitivity of the model

We examined how the model responds to varying assumptions on the size of the simulation domain and the number of species. Furthermore, we considered a completely neutral variant of the model, where the whole species pool shared the same singe limiting resource. In all cases, we observed that the general patterns remain qualitatively similar for all experiments. Due to space constraints, these results are deferred to Appendix C.

## Discussion

The question of how landscape structure and fragmentation *per se* affect species richness has been long debated. The habitat amount hypothesis posits that fragmentation *per se* does not have a strong effect, but it is only the total amount of habitat in a local landscape that matters (Fahrig 2013). In this work, we investigated how fragmentation *per se* influences species richness in competitive metacommunities and when does the habitat amount hypothesis hold in such communities following the tests outlined by Fahrig (2013).

Our main conclusions can be summarised as follows. First, fragmentation has non‐trivial interactions with habitat loss: it is not unequivocally either good or bad (Fletcher *et al*. 2018; Fahrig *et al*. 2019). At high amounts of habitat, fragmentation *per se* matters little, but when the amount of available habitat decreases, fragmentation starts to exhibit complex effects on species richness, which range from negative, unimodal or positive (Fig. 4). In particular, for conservation purposes, this suggests that increasing fragmentation may be detrimental if little habitat is available, but intermediate degrees of fragmentation may be beneficial for competitive communities when the amount of habitat is fairly high (Figs 2 and 4).

Second, concerning the habitat amount hypothesis, we see that even in our idealised setting with synthetic data, one cannot easily get clear‐cut support or refutation for the hypothesis when following the tests outlined by Fahrig (2013). The extent of the response depends on total amount of habitat, the dispersal range of the species, and other the community properties. Furthermore, different analyses on the same data can seemingly exhibit different responses to fragmentation (see e.g. Figs 2 and 3). This highlights that caution is needed when inferring whether empirical data shows positive or negative effects of fragmentation.

Third, we observed that the habitat amount hypothesis is sensitive to the scale at which ‘local landscapes’ are defined. Given that our data is from an idealised simulation model with no sampling error and much simpler underlying processes than can be expected for real communities (e.g. all species had the same dispersal distance and responses to the matrix), this may indicate that inferring the appropriate scales necessary for the hypothesis is difficult with real‐world (limited and noisy) empirical data.

Naturally our modelling work has its limitations. First and foremost, our results should not be used as such to infer *quantitative* extinction thresholds for real‐world communities. The exact numerical thresholds for the total amount of habitat, at which fragmentation effects become prominent, will naturally depend on the ecology of the species and the spatial scale of the dynamics (e.g. size of the focal landscape compared to dispersal distances). Thus, our results do not directly convey specific values for ‘low’ and ‘high’ amounts of habitat, as these will be context‐dependent.

Second, we limited our attention to fairly simple species communities, where all species are ecologically similar. While real communities arguably are more complex, we observe that even under these simplistic assumptions habitat fragmentation can influence species richness in multitude of ways: fragmentation *per se* can show *both* negative and positive effects on species richness in competitive metacommunities, and intermediate levels of fragmentation may sometimes positively influence species richness. Interestingly, the non‐monotone patterns similar to those predicted by our model were recently been observed in an experimental fragmentation study (Loke *et al*. 2019).

Our modelling approach simplifies the examination of the habitat amount hypothesis: all species in the community (1) share the same habitat type (making it simpler to define what parts of the landscape counts as habitat) and (2) have identical responses at different spatial scales (making it easier to infer the scale of local landscape and tests its effects). Even in such an idealised setting, clear support for the habitat amount hypothesis remains elusive. This suggests that either (1) the habitat amount hypothesis does not hold in all situations, (2) the hypothesis needs to be refined or (3) our model lacks some critical features. Naturally, all models are simplifications of reality and omit various details. Prior models have been criticised, for example De Camargo *et al*. (2018) suggest that ‘model of area‐dependent, stochastic patch occupancy and extinction’ by Rybicki & Hanski (2013) ‘fails to capture some critical aspect of habitat loss’, and hence, exhibit adverse effects of fragmentation. Nevertheless, in this work, we used a model with fundamentally different structural assumptions and yet still obtain the same qualitative results, and our results are consistent with prior theory and modelling work (Tilman *et al*. 1997; Rybicki & Hanski 2013). Indeed, classic metapopulation theory predicts that species persistence in fragmented landscapes is influenced not only by just dispersal distances, extinction–colonisation dynamics and habitat quality, but also by the spatial configuration of the habitat (Hanski & Ovaskainen 2000, 2003).

Whether or not our model captures all key aspects governing species dynamics under fragmentation, mathematical and conceptual models can still help shed light on which mechanisms drive the community patterns emerging in fragmented landscapes. Thus, even if the habitat amount hypothesis holds, the underlying mechanisms are poorly understood. Our work suggests that the hypothesis should rely on some mechanisms not accounted in our or any of the prior models, for example fast eco‐evolutionary responses (Legrand *et al*. 2017) or specific metacommunity dynamics (Leibold *et al*. 2004). Furthermore, not all species are equally sensitive to habitat fragmentation and how different species tolerate the matrix varies (Gascon *et al*. 1999). In this work, we restricted our attention to competitive habitat specialists, and thus, areas inflicted with habitat loss cannot sustain any species (unless near habitat). In particular, we do not consider *habitat conversion*, where one type of habitat is converted to another, which may still be suitable to some existing species or new species that can replace the original species in the species pool.

Beyond examining the effects of fragmentation effects on species richness, we believe that our modelling framework lends itself to be a useful tool for spatially explicit investigations of various other species interactions in metacommunities. So far, our work only considers communities of competing habitat specialists, and thus, it could be extended to other community structures and dynamics, such as predator–prey dynamics, competition–colonisation tradeoffs, mutualist species or successive metacommunities. Such extensions may reveal how responses to fragmentation depend on specific of metacommunity dynamics. Indeed, Wilson *et al*. (2016) point out that ‘other measures of community structure, such as community composition, trophic organization, species persistence, and species residency, may better inform how fragmentation affects biotic communities, even when species richness *per se* is not altered by fragmentation’. Therefore, we conclude that it may be time to move on from debating whether fragmentation matters or not, onto developing a comprehensive and fine‐grained understanding of *when* and *how* fragmentation matters.

## Authorship

All authors designed the research. JR implemented the model, conducted the experiments, performed analyses, wrote and revised the manuscript. NA edited the manuscript. OO conducted the statistical analyses and edited the manuscript.

## Data Accessibility Statement

The source code and data related to the manuscript has been placed in a public online repository: https://doi.org/10.5281/zenodo.3552447

## References

[ele13450-bib-0001] Arnillas, C.A. , Tovar, C. , Cadotte, M.W. & Buytaert, W. (2017). From patches to richness: assessing the potential impact of landscape transformation on biodiversity. Ecosphere, 8(11), e02004.

[ele13450-bib-0002] Bascompte, J. & Solé, R.V. (1996). Habitat fragmentation and extinction thresholds in spatially explicit models. J. Anim. Ecol., 65(4), 465.

[ele13450-bib-0003] Butchart, S.H.M. , Walpole, M. , Collen, B. , van Strien, A. , Scharlemann, J.P.W. , Almond, R.E.A. *et al* (2010). Global biodiversity: Indicators of recent declines. Science, 328(5982), 1164–1168. 20430971 10.1126/science.1187512

[ele13450-bib-0004] Cornell, S.J. , Suprunenko, Y.F. , Finkelshtein, D. , Somervuo, P. & Ovaskainen, O. (2019). A unified framework for analysis of individual‐based models in ecology and beyond. Nat. Commun., 10(4716). 10.1038/s41467-019-12172-y. PMC6797757 31624268

[ele13450-bib-0005] Damschen, E.I. , Brudvig, L.A. , Burt, M.A. , Fletcher, R.J. , Haddad, N.M. , Levey, D.J. *et al* (2019). Ongoing accumulation of plant diversity through habitat connectivity in an 18‐year experiment. Science, 365(6460), 1478–1480. 31604279 10.1126/science.aax8992

[ele13450-bib-0006] De Camargo, R.X. , Boucher‐Lalonde, V. & Currie, D.J. (2018). At the landscape level, birds respond strongly to habitat amount but weakly to fragmentation. Divers. Distrib., 24(5), 629–639.

[ele13450-bib-0007] Diamond, J.M. (1975). The island dilemma: lessons of modern biogeographic studies for the design of natural reserves. Biol. Cons., 7(2), 129–146.

[ele13450-bib-0008] Didham, R.K. , Kapos, V. & Ewers, R.M. (2012). Rethinking the conceptual foundations of habitat fragmentation research. Oikos, 121(2), 161–170.

[ele13450-bib-0009] Ewers, R.M. & Didham, R.K. (2005). Confounding factors in the detection of species responses to habitat fragmentation. Biol. Rev., 81(01), 117. 16318651 10.1017/S1464793105006949

[ele13450-bib-0010] Fahrig, L. (2003). Effects of habitat fragmentation on biodiversity. Annu. Rev. Ecol. Evol. Syst., 34(1), 487–515.

[ele13450-bib-0011] Fahrig, L. (2013). Rethinking patch size and isolation effects: The habitat amount hypothesis. J. Biogeogr., 40(9), 1649–1663.

[ele13450-bib-0012] Fahrig, L. (2015). Just a hypothesis: a reply to Hanski. J. Biogeogr., 42(5), 993–994.

[ele13450-bib-0013] Fahrig, L. (2017). Ecological responses to habitat fragmentation per se. Annu. Rev. Ecol. Evol. Syst., 48(1), 1–23.

[ele13450-bib-0014] Fahrig, L. , Arroyo‐Rodríguez, V. , Bennett, J.R. , Boucher‐Lalonde, V. , Cazetta, E. , Currie, D.J. *et al* (2019). Is habitat fragmentation bad for biodiversity? Biol. Cons., 230, 179–186.

[ele13450-bib-0015] Fletcher, R.J. , Didham, R.K. , Banks‐Leite, C. , Barlow, J. , Ewers, R.M. , Rosindell, J. *et al* (2018). Is habitat fragmentation good for biodiversity? Biol. Cons., 226, 9–15.

[ele13450-bib-0016] Gascon, C. , Lovejoy, T.E. , Bierregaard Jr, R.O. , Malcolm, J.R. , Stouffer, P.C. Vasconcelos, H.L. *et al* (1999). Matrix habitat and species richness in tropical forest remnants. Biol. Cons., 91(2), 223–229.

[ele13450-bib-0017] Gilarranz, L.J. & Bascompte, J. (2012). Spatial network structure and metapopulation persistence. J. Theor. Biol., 297, 11–16. 22155351 10.1016/j.jtbi.2011.11.027

[ele13450-bib-0018] Grilli, J. , Barabás, G. & Allesina, S. (2015). Metapopulation persistence in random fragmented landscapes. PLoS Comput. Biol., 11(5), e1004251. 25993004 10.1371/journal.pcbi.1004251 PMC4439033

[ele13450-bib-0019] Haddad, N.M. , Brudvig, L.A. , Clobert, J. , Davies, K.F. , Gonzalez, A. , Holt, R.D. *et al* (2015). Habitat fragmentation and its lasting impact on earth's ecosystems. Sci. Adv., 1(2), e1500052. 26601154 10.1126/sciadv.1500052 PMC4643828

[ele13450-bib-0020] Haddad, N.M. , Gonzalez, A. , Brudvig, L.A. , Burt, M.A. , Levey, D.J. & Damschen, E.I. (2017). Experimental evidence does not support the habitat amount hypothesis. Ecography, 40(1), 48–55.

[ele13450-bib-0021] Hanski, I. (1999). Metapopulation Ecology. Oxford University Press, Oxford.

[ele13450-bib-0022] Hanski, I. (2015). Habitat fragmentation and species richness. J. Biogeogr., 42(5), 989–993.

[ele13450-bib-0023] Hanski, I. & Ovaskainen, O. (2000). The metapopulation capacity of a fragmented landscape. Nature, 404(6779), 755–758. 10783887 10.1038/35008063

[ele13450-bib-0024] Hanski, I. & Ovaskainen, O. (2003). Metapopulation theory for fragmented landscapes. Theor. Popul. Biol., 64(1), 119–127. 12804876 10.1016/s0040-5809(03)00022-4

[ele13450-bib-0025] Hanski, I. , Zurita, G.A. , Bellocq, M.I. & Rybicki, J. (2013). Species‐fragmented area relationship. Proc. Natl Acad. Sci., 110(31), 12715–12720. 23858440 10.1073/pnas.1311491110 PMC3732936

[ele13450-bib-0026] Henle, K. , Davies, K.F. , Kleyer, M. , Margules, C. & Settele, J. (2004). Predictors of species sensitivity to fragmentation. Biodivers. Conserv., 13(1), 207–251.

[ele13450-bib-0027] Jackson, H.B. & Fahrig, L. (2012). What size is a biologically relevant landscape? Landscape Ecol., 27(7), 929–941.

[ele13450-bib-0028] Kahilainen, A. , Nouhuys, S. , Schulz, T. & Saastamoinen, M. (2018). Metapopulation dynamics in a changing climate: Increasing spatial synchrony in weather conditions drives metapopulation synchrony of a butterfly inhabiting a fragmented landscape. Glob. Change Biol., 24(9), 4316–4329. 10.1111/gcb.14280 PMC6120548 29682866

[ele13450-bib-0029] Kuussaari, M. , Bommarco, R. , Heikkinen, R.K. , Helm, A. , Krauss, J. , Lindborg, R. *et al* (2009). Extinction debt: a challenge for biodiversity conservation. Trends Ecol. Evol., 24(10), 564–571. 19665254 10.1016/j.tree.2009.04.011

[ele13450-bib-0030] Legrand, D. , Cote, J. , Fronhofer, E.A. , Holt, R.D. , Ronce, O. , Schtickzelle, N. *et al* (2017). Eco‐evolutionary dynamics in fragmented landscapes. Ecography, 40(1), 9–25.

[ele13450-bib-0031] Leibold, M.A. , Holyoak, M. , Mouquet, N. , Amarasekare, P. , Chase, J.M. , Hoopes, M.F. *et al* (2004). The metacommunity concept: a framework for multi‐scale community ecology. Ecol. Lett., 7(7), 601–613.

[ele13450-bib-0032] Loke, L.H.L. , Chisholm, R.A. & Todd, P.A. (2019). Effects of habitat area and spatial configuration on biodiversity in an experimental intertidal community. Ecology, 100(8), e02757. 31062341 10.1002/ecy.2757 PMC6851599

[ele13450-bib-0033] Lomolino, M. & Weiser, M. (2001). Towards a more general species‐area relationship: diversity on all islands, great and small. J. Biogeogr., 28(4), 431–445.

[ele13450-bib-0034] MacArthur, R.H. & Wilson, E.O. (1967). The Theory of Island Biogeography. Princeton University Press, Princeton, NJ.

[ele13450-bib-0035] MacDonald, Z.G. , Anderson, I.D. , Acorn, J.H. & Nielsen, S.E. (2018). The theory of island biogeography, the sample‐area effect, and the habitat diversity hypothesis: complementarity in a naturally fragmented landscape of lake islands. J. Biogeogr., 45(12), 2730–2743.

[ele13450-bib-0036] Melo, G.L. , Sponchiado, J. , Cáceres, N.C. & Fahrig, L. (2017). Testing the habitat amount hypothesis for south american small mammals. Biol. Cons., 209, 304–314.

[ele13450-bib-0037] Ovaskainen, O. (2002). Long‐term persistence of species and the SLOSS problem. J. Theor. Biol., 218(4), 419–433. 12384046

[ele13450-bib-0038] Ovaskainen, O. , Finkelshtein, D. , Kutoviy, O. , Cornell, S. , Bolker, B. & Kondratiev, Y. (2014). A general mathematical framework for the analysis of spatiotemporal point processes. Theor. Ecol., 7(1), 101–113.

[ele13450-bib-0039] Pereira, H.M. , Leadley, P.W. , Proenca, V. , Alkemade, R. , Scharlemann, J.P.W. , Fernandez‐Manjarres, J.F. *et al* (2010). Scenarios for global biodiversity in the 21st century. Science, 330(6010), 1496–1501. 20978282 10.1126/science.1196624

[ele13450-bib-0040] Pimm, S.L. , Jenkins, C.N. , Abell, R. , Brooks, T.M. , Gittleman, J.L. , Joppa, L.N. *et al* (2014). The biodiversity of species and their rates of extinction, distribution, and protection. Science, 344(6187), 1246752. 24876501 10.1126/science.1246752

[ele13450-bib-0041] Rosenzweig, M. (1995). Species diversity in space and time. Cambridge University Press.

[ele13450-bib-0042] Rybicki, J. & Hanski, I. (2013). Species‐area relationships and extinctions caused by habitat loss and fragmentation. Ecol. Lett., 16, 27–38. 23452159 10.1111/ele.12065

[ele13450-bib-0043] Sfenthourakis, S. & Triantis, K.A. (2009). Habitat diversity, ecological requirements of species and the small island effect. Divers. Distrib., 15(1), 131–140.

[ele13450-bib-0044] Thomas, C.D. , Cameron, A. , Green, R.E. , Bakkenes, M. , Beaumont, L.J. , Collingham, Y.C. *et al* (2004). Extinction risk from climate change. Nature, 427(6970), 145–148. 14712274 10.1038/nature02121

[ele13450-bib-0045] Thompson, P.L. , Rayfield, B. & Gonzalez, A. (2017). Loss of habitat and connectivity erodes species diversity, ecosystem functioning, and stability in metacommunity networks. Ecography, 40(1), 98–108.

[ele13450-bib-0046] Tilman, D. , May, R.M. , Lehman, C.L. & Nowak, M.A. (1994). Habitat destruction and the extinction debt. Nature, 371(6492), 65–66.

[ele13450-bib-0047] Tilman, D. , Lehman, C.L. & Yin, C. (1997). Habitat destruction, dispersal, and deterministic extinction in competitive communities. Am. Nat., 149(3), 407–435.

[ele13450-bib-0048] Vieira, M.V. , Almeida‐Gomes, M. , Delciellos, A.C. , Cerqueira, R. & Crouzeilles, R. (2018). Fair tests of the habitat amount hypothesis require appropriate metrics of patch isolation: an example with small mammals in the Brazilian Atlantic Forest. Biol. Cons., 226, 264–270.

[ele13450-bib-0049] Williams, C.B. (1964). Patterns in the Balance of Nature. Academic Press, New York.

[ele13450-bib-0050] Wilson, M.C. , Chen, X.‐Y. , Corlett, R.T. , Didham, R.K. , Ding, P. , Holt, R.D. *et al* (2016). Habitat fragmentation and biodiversity conservation: key findings and future challenges. Landscape Ecol., 31(2), 219–227.

