# Supplementary material for: Habitat fragmentation and species diversity in competitive communities
Source: Ecol Lett. 2019 Dec 20;23(3):506–17. doi: 10.1111/ele.13450 (PMC7027465; doi:10.1111/ele.13450)
Supplement: Supplementary file 1 [file ELE-23-506-s001.pdf]

Appendix: Supplementary material for “Habitat fragmentation and species diversity in competitive communities”

November 19, 2019

Joel Rybicki · joel.rybicki@ist.ac.at

Nerea Abrego · nerea.abrego@helsinki.fi

Otso Ovaskainen · otso.ovaskainen@helsinki.fi

Contents

A Prior work on metacommunity models 4

B Description of the simulation model 5

B.1 Details of the spatially-explicit community model 5

B.1.1 Model structure 5

B.1.2 Resource patch dynamics 6

B.1.3 Species dynamics 6

B.1.4 Comparison to a deterministic, non-spatial model 7

B.1.5 Dispersal 8

B.1.6 Habitat fragmentation 9

B.1.7 Large-scale environmental variation in habitat types 9

B.1.8 Simulation of the individual-based model 11

B.2 Meanfield approximation of species dynamics 11

B.3 Displacement of resource-deprived individuals 13

C Sensitivity of the simulation model and additional experiments 14

C.1 Convergence to the stationary state 14

C.2 A neutral model with no variation in resource types 14

C.3 Sensitivity to the number of species 14

C.4 Sensitivity to the size of the landscape 15

C.5 Testing the sample area effect with larger sampling sites 15

C.6 An alternative parameterisation of the individual-based model 16

List of Figures

S1 Examples of fragmented landscapes . . . 18
S2 Comparison of the spatial stochastic model against the deterministic meanfield model 19
S3 The average density of resource-satiated individuals vs. time . . . 20
S4 SLOSS analysis, neutral community, 128 species . . . 21
S5 SFAR analysis, neutral community, 128 species . . . 22
S6 Species richness vs. fragmentation, neutral community, 128 species . . . 23
S7 SLOSS analysis, neutral community, 64 species . . . 24
S8 SFAR analysis, neutral community, 64 species . . . 25
S9 Species richness vs. fragmentation, neutral community, 64 species . . . 26
S10 Inferring local scale for HAH, neutral community, 64 species, $\tau = 1$ . . . 27
S11 Inferring local scale for HAH, neutral community, 64 species, $\tau = 2$ . . . 28
S12 SLOSS analysis, environmental gradient, 64 species . . . 29
S13 SFAR analysis, environmental gradient, 64 species . . . 30
S14 Species richness vs. fragmentation, environmental gradient, 64 species . . . 31
S15 Inferring local scale for HAH, environmental gradient, 64 species, $\tau = 1$ . . . 32
S16 Inferring local scale for HAH, environmental gradient, 64 species, $\tau = 2$ . . . 33
S17 SLOSS analysis, neutral community, 32 species . . . 34
S18 SFAR analysis, neutral community, 32 species . . . 35
S19 Species richness vs. fragmentation, neutral community, 32 species . . . 36
S20 Inferring local scale for HAH, neutral community, 32 species, $\tau = 1$ . . . 37
S21 Inferring local scale for HAH, neutral community, 32 species, $\tau = 2$ . . . 38
S22 SLOSS analysis, environmental gradient, 32 species . . . 39
S23 SFAR analysis, environmental gradient, 32 species . . . 40
S24 Species richness vs. fragmentation, environmental gradient, 32 species . . . 41
S25 Inferring local scale for HAH, environmental gradient, 32 species, $\tau = 1$ . . . 42
S26 Inferring local scale for HAH, environmental gradient, 32 species, $\tau = 2$ . . . 43
S27 SLOSS analysis in a small 25×25 landscape . . . 44
S28 SFAR analysis in a small 25×25 landscape . . . 45
S29 Species richness vs. fragmentation in a small 25×25 landscape . . . 46
S30 SLOSS analysis in a small 25×25 landscape with an environmental gradient . . . 47

S31 SFAR analysis in a small 25×25 landscape with an environmental gradient 48
S32 Species richness vs. fragmentation in a small 25×25 landscape with an environmental-gradient 49
S33 Inferring local scale for HAH, 128 species, environmental gradient, $\tau = 2$ 50
S34 SLOSS analysis, alternative neutral parameterisation 51
S35 SFAR analysis, alternative neutral parameterisation 52
S36 Species richness vs. fragmentation, alternative neutral parameterisation 53
S37 SLOSS analysis, alternative parameterisation, environmental gradient 54
S38 SFAR analysis, alternative parameterisation, environmental gradient 55
S39 Species richness vs. fragmentation, alternative parameterisation, environmental gradient 56

List of Tables

S1 Parameters of the community model and their default values 10
S2 Results for the HAH test in landscapes with sampling site radius $\tau = 2$ 15
S3 Results for the HAH test in neutral communities of 128 species, sampling site radius $\tau = 1$ 15
S4 Results for the HAH test in neutral communities of 128 species, sampling site radius $\tau = 2$ 16

A Prior work on metacommunity models

We focus on species dynamics at the landscape level in fragmented environments, that is, metacommunity dynamics. While a metapopulation is a collection of populations (of a single species) that reside in discrete patches connected by dispersal, the metacommunity concept is its multispecies analogue (Hanski, 1999): patches are inhabited by several (possibly interacting) species and the metacommunity dynamics are governed by various coexistence processes (Leibold et al., 2004). Typically, theoretical studies on metacommunities have generalised patch-based metapopulation models into multispecies models with discrete patches in both spatially-implicit (Tilman et al., 1994, Loreau et al., 2003, Mouquet and Loreau, 2003, Allouche and Kadmon, 2009, Wang and Loreau, 2016) and spatially-explicit models (Solé et al., 2004, Rybicki and Hanski, 2013, Matias et al., 2014, Thompson et al., 2014, Fournier et al., 2016, Thompson et al., 2017). The interspecies interactions are typically competitive (e.g. competition on space or a shared limiting resource) (Tilman et al., 1994, Loreau et al., 2003, Mouquet and Loreau, 2003, Solé et al., 2004, Wang and Loreau, 2016, Matias et al., 2014, Thompson et al., 2014, Fournier et al., 2016, Thompson et al., 2017), but also mutualistic (e.g. plant-pollinator systems) (Klausmeier, 2001, Prakash and de Roos, 2004, Fortuna and Bascompte, 2006) and trophic interactions (Pillai et al., 2010) have been considered.

A relatively large proportion of the theoretical work on metacommunities has focused on understanding how e.g. coexistence mechanisms and dispersal maintain species diversity and stability (Lehman and Tilman, 2000, Loreau et al., 2003, Mouquet and Loreau, 2003, Gravel et al., 2006, Logue et al., 2011, Haegeman and Loreau, 2014, Wang and Loreau, 2016, Gravel et al., 2016). While less common, models have also been used to investigate how landscape structure, habitat loss and fragmentation influence species richness in metacommunities (Tilman et al., 1997, Solé et al., 2004, Prakash and de Roos, 2004, Rybicki and Hanski, 2013, Matias et al., 2014, Thompson et al., 2014, 2017, Xu et al., 2018).

As usual, these models make various assumptions for the sake of tractability: some completely ignore species interactions and rely on species-sorting mechanisms (Rybicki and Hanski, 2013), limit to only pairs of interacting species (Klausmeier, 2001), ignore spatial heterogeneity, assume patch-based metapopulation dynamics (Tilman et al., 1997, Prakash and de Roos, 2004, Rybicki and Hanski, 2013, Matias et al., 2014) or lack demographic and/or environmental stochasticity by employing deterministic, continuous-valued dynamics (Thompson et al., 2014).

B Description of the simulation model

B.1 Details of the spatially-explicit community model

We first define the model dynamics in a pristine landscape without habitat loss. After this, we discuss ways how to include habitat loss and fragmentation into our model. For brevity, we describe the model in the context of a single species consuming a single resource type, but the model is straightforward to generalise to multiple species and resource types.

B.1.1 Model structure

Formally, our model is a spatiotemporal point process, or in the mathematical terminology, a Markov evolution in the space of locally finite configurations (see e.g. Ovaskainen et al., 2014). The state of a model at any given time is given by the spatial location of each individual of each type. The dynamics of the model can be described by listing all events that can take place and the rates at which these events occur. These rates can depend on the current spatial configuration of all individuals (e.g. individuals can only consume resources that are within their proximity). The proximity-dependence is described via kernels, that is, functions that tell at which rate two particles at locations $x = (x_1, x_2)$ and $y = (y_1, y_2)$ react depending on their Euclidean distance $\mathrm{dist}(x, y)$. A kernel $K(x, y) = r \cdot f(x, y)$ is defined in terms of a total rate parameter r and a density function f, which describes how the total rate r is distributed accross the space.

We use two types of kernels. A *tophat* kernel K with length scale ℓ and total rate r is defined as

$$K(x, y) = \begin{cases} \frac{r}{2\pi\ell^2} & \text{if } \mathrm{dist}(x, y) \leq \ell, \\ 0 & \text{otherwise.} \end{cases}$$

A *Gaussian* kernel K with length scale ℓ and total rate r is given by

$$K(x, y) = A(r) \cdot \exp\left(-\frac{\mathrm{dist}(x, y)^2}{2\ell^2}\right),$$

where $A(r) = r/(2\pi\ell^2)$ is a normalisation constant such that K integrates to the value r. In the following, we write $\ell(K)$ and $r(K)$ to denote the length scale and total rate of kernel K of either type.

B.1.2 Resource patch dynamics

To obtain spatiotemporal variation in resource production rate, we assume that the resource patches follow birth-death dynamics independent of the species. The resource patch birth-death dynamics are described by the following processes

$$\emptyset \xrightarrow{\beta} \mathrm{P} \qquad \text{new resource patches appear at per unit area rate } \beta,$$

$$\mathrm{P} \xrightarrow{\gamma} \emptyset \qquad \text{resource patches disappear at per capita rate } \gamma,$$

where $\emptyset$ denotes "no particle" (i.e. birth of resource patches does not depend on any entity and the death of a patch simply removes the patch but not the resource units it has produced). We assume that the resource patches are circular (but may overlap) and that a resource patch located at x produces abiotic resource units to its surroundings according to the tophat resource generation kernel G. Resource units that are left unconsumed by the species decay and disappear with constant rate κ. Thus, we have the following processes:

$$\underset{x}{\mathrm{P}} \xrightarrow{G(x,y)} \underset{x}{\mathrm{P}} + \underset{y}{\mathrm{R}} \qquad \text{a patch centered at } x \text{ generates a resource to } y \text{ at per unit area rate } G(x,y),$$

$$\mathrm{R} \xrightarrow{\kappa} \emptyset \qquad \text{unused resources decay at per capita rate } \kappa,$$

where in the first reaction, the location of a new particle is randomly sampled according to the density function f of the kernel $G(x,y) = r \cdot f(x,y)$. Note that we can control the spatiotemporal properties of the landscape's habitat quality by varying the per unit area density $\rho = \beta/\gamma$ of habitat patches, the patch turnover rate γ, the resource production rate $r = r(G)$, and the radius $\ell(G)$ of the patches. First panel of Fig. S1 depicts an example snapshot of the resulting habitat structure.

B.1.3 Species dynamics

The species follow birth-death dynamics and individuals have two states. Newborn individuals start out in the *resource-deprived* state, and upon consuming a resource unit, they become *resource-satiated*; for brevity, we refer to individuals in these states as deprived (D) and satiated (S), respectively. The

satiated individuals produce new individuals into their surroundings:

$$\underset{x}{\mathrm{D}} + \underset{y}{\mathrm{R}} \xrightarrow{U(x,y)} \underset{x}{\mathrm{S}} \qquad \text{a deprived individual at } x \text{ consumes a resource at } y \text{ at rate } U(x,y)$$

$$\underset{x}{\mathrm{S}} \xrightarrow{B(x,y)} \underset{x}{\mathrm{S}} + \underset{y}{\mathrm{D}} \qquad \text{a satiated individual produces progeny to } y \text{ at per unit area rate } B(x,y),$$

where U is a tophat resource utilisation kernel, B is a Gaussian birth kernel, and the satiated individuals give birth to new individuals at total per capita rate of $r(B)$ and the locations of the new deprived individuals are sampled according to the density function of B. Eventually, satiated individuals become resource-deprived, and if they do not consume resources, they die:

$$\mathrm{S} \xrightarrow{\eta} \mathrm{D} \qquad \text{satiated individuals return to deprived state at per capita rate } \eta,$$

$$\mathrm{D} \xrightarrow{\mu} \emptyset \qquad \text{deprived individuals die at per capita rate } \mu.$$

In addition to birth by resource-satiated individuals, we assume that there is a small background rate of influx immigration into the focal landscape:

$$\emptyset \xrightarrow{\alpha} \mathrm{D} \qquad \text{new resource-deprived individuals arrive at per unit area rate of } \alpha.$$

B.1.4 Comparison to a deterministic, non-spatial model

As a brief digression, let us check that the above resource-consumer model makes sense by considering the deterministic dynamics in a non-spatial setting, which is easy to analyse (see Appendix B.2). There exists a unique positive equilibrium of satiated individuals if all rates are positive. In case $\alpha = 0$, that is, when there is no immigration of individuals outside the focal landscape, the species goes extinct if the total resource production rate is not sufficiently high. More precisely, there exists an extinction threshold of

$$\frac{G\beta}{\kappa\gamma} > \frac{\eta\mu}{BU},$$

where G, B, U correspond to the total rates (i.e. integrals) of the respective kernels. Thus, the meanfield approximation exhibits the expected dynamics of resource-consumer models: without immigration, the resource equilibrium level determines whether the consumer can persist in the system. Now let us return to the stochastic, spatially-explicit and individual-based model with heterogeneous habitat structure.

B.1.5 Dispersal

We consider two modes of dispersal for the species:

- *passive* (one-shot) dispersal, where an individual does not move during its life time,
- *active* dispersal, where resource-deprived individuals move to find suitable habitat.

For passive dispersal, individuals only move when they are born according to the birth kernel B centered around their progenitor. In active dispersal, we include an additional movement process for resourced-deprived individuals:

$$\underset{x}{\mathrm{D}} \xrightarrow{M(x,y)} \underset{y}{\mathrm{D}} \quad \text{resource-deprived individuals jump from } x \text{ to } y \text{ at per unit area rate } M(x,y).$$

The idea is that in the active mode of dispersal, resource-deprived individuals can make additional movements to move from a location lacking resources to a new location with resources. Once an individual finds resources and consumes them, it becomes resource-satiated and stops moving.

We control the length of dispersal with the scale parameter δ and the mode of dispersal with an integer parameter $k > 0$. We set length scales of the Gaussian birth and movement kernels to $\ell(B) = \ell(M) = \delta/\sqrt{k}$ and set the movement rate to $r(M) = (k-1)\mu$. Passive dispersal corresponds to case $k = 1$, as no movement after birth occurs. In active dispersal, we have $k > 1$. Thus, species with passive dispersal are sessile, whereas species with active dispersal are not.

Note that regardless of the value k, the new location of an actively dispersing resource-deprived individual during its lifetime has the same mean and variance as a passive disperser *assuming the individual does not become satiated at any point in its lifetime*. If an individual remains resource-deprived its entire lifetime, then the number of dispersal steps s is a random variable with an expectation of $r(M)/\mu$ (see Appendix B.3). Hence, as an individual always make a single movement step at birth, the total displacement is a random variable $x = (x_1, x_2)$ that satisfies

$$x_i | s = \sum_{j=0}^{s} x_{ij},$$

where $i \in \{1,2\}$ and $x_{ij} \sim \mathcal{N}(0, \delta^2/k)$ are independent Gaussian variables. It follows that x_i has the same mean 0 and variance δ^2 in both the active and passive dispersal modes (see Appendix B.3).

B.1.6 Habitat fragmentation

Above we have described the model in a pristine landscape with no habitat loss or fragmentation; while the habitat quality (resource production rate) can have patchy structure, the spatiotemporal dynamics of the resource patches guarantee that every location of the landscape has statistically the same properties over time.

We conduct our simulations in a finite focal landscape of size $V \times V$, which is assumed to be part of a larger landscape that has statistically similar structure evewhere. To avoid boundary effects, we assume it to be a two-dimensional torus $\mathscr{L} \subseteq \mathbb{R}^2$. In order to model habitat loss and fragmentation, we assume that the focal landscape $\mathscr{L}$ is partitioned into N disjoint (non-overlapping) circular *habitat fragments*, where the habitat fragment i is centered at location x_i and consists of all points within radius r_i. The *matrix* $\mathscr{M}$ then consists of the points that are not part of any habitat fragment. We emphasise that the notions of *habitat fragment* and *resource patch* (an entity that produces abiotic resource units into its surroundings) refer to distinct concepts in our model.

We generate *fragmented* landscapes where the patch sizes follow a log-normal distribution so that there are patches of various sizes. Given the total habitat cover $0 < C < 1$ and number of fragments $N > 0$, we sample the relative area of each fragment $A_i \sim \exp \mathscr{N}(\mu, \sigma^2)$ with parameters $\mu = 1/2$ and $\sigma = 1$ for every fragment $i \in \{1, \dots, N\}$ and normalise the areas so that $\sum A_i = C \cdot V^2$.

The fragments are placed onto the landscape in a decreasing order in area and both coordinates of the fragment i are sampled uniformly at random from the interval $[0, V]$, where V is the parameter controlling the size of the focal landscape. The coordinates of fragment i are resampled until there is no overlap with any fragment $j < i$ to ensure that all fragments are disjoint. Fig. S1 illustrates examples of fragmented landscapes for different values of C and N. For the special case $C = 1$, we assume that the entire landscape is covered by a single fragment.

B.1.7 Large-scale environmental variation in habitat types

In the main text, we consider a variant of the model with large-scale environmental variation in habitat types. Here, the species are divided into four groups, each of which is specialised to a certain type of a resource. The resource distributions follow different environmental gradients, but the overall resource production rate is the same for each resource type throughout the landscape (in a non-fragmented setting). Thus, while the species rely on different resource types, the species are equivalent in the sense of having the same expected fitness: all species have the same parameters

Table S1: Parameters of the community model and their default values

Process	Name	Value / Rate (r)	Length scale (ℓ)
Patch birth rate (per unit area)	β	0.01	–
Patch turnover/death rate (per capita)	γ	0.1	–
Resource decay rate (per capita)	κ	0.1	–
Resource production kernel (tophat)	G	4	2
Resource utilisation kernel (tophat)	U	1	0.5
Resource deprivation rate (per capita)	η	0.1	–
Mortality rate (per capita)	μ	1	–
Background immigration rate (per unit area)	α	0.001	–
Dispersal scale parameter	δ	1, 3, 10	–
Mode of dispersal parameter	k	4	–
Birth kernel (Gaussian)	B	1	$\delta/\sqrt{k}$
Movement kernel (Gaussian)	M	$(k-1)\mu$	$\delta/\sqrt{k}$

(Table S1) and the landscape produces all types of resource at the same rate. Later below, we also consider a *completely neutral* model (Hubbell, 2001), where all species are identical and consume exactly the same resource type.

Generating large-scale environmental variation. We assumed that there were four distinct resource patch types each producing a distinct resouce type. Each species i was then specialised to consume only resources of type $i \bmod 4 + 1$. To produce environmental gradients for the habitat types, the location of a new resource patch of type $j \in \{1,2,3,4\}$ was sampled according to the density function $g_j(x,y)$ on $[0,1]^2$, where

$$\begin{aligned} g_1(x,y) &= 1+\sin(2\pi x) \\ g_2(x,y) &= 1+\sin(2\pi x+\pi) \\ g_3(x,y) &= 1+\sin(2\pi y) \\ g_4(x,y) &= 1+\sin(2\pi y+\pi). \end{aligned}$$

The sampled coordinates were then scaled to the domain size of $V \times V$ of the focal landscape. In this setting, the landscapes are heterogeneous also in terms of habitat *types* in addition to habitat quality. We conducted the experiments described in the main text also for the nine scenarios under (1) specialist communities in landscapes with large-scale environmental gradients in habitat types and (2) completely neutral communities with no variation in habitat types.

B.1.8 Simulation of the individual-based model

To simulate the individual-based model, we use the Gibson–Bruck next reaction method (Gibson and Bruck, 2000), a Gillespie-style simulation algorithm (Gillespie, 1976). The algorithm produces exact stochastic trajectories of the stochastic individual-based model. The algorithm is adapted to the spatial setting similarly as done by Cornell et al. (2019). The source code for the simulation software used in this work is available online (Rybicki et al., 2019).

B.2 Meanfield approximation of species dynamics

Let us examine the single-species dynamics in a non-spatial setting. If we assume that the system is well-mixed and let the length scale $\ell \to \infty$ for each kernel, then the deterministic meanfield model can be written as

$$\begin{aligned}\frac{\mathrm{d}P}{\mathrm{d}t} &= \beta - \gamma P\\ \frac{\mathrm{d}R}{\mathrm{d}t} &= GP - \kappa R - UDR\\ \frac{\mathrm{d}S}{\mathrm{d}t} &= UDR - \eta S\\ \frac{\mathrm{d}D}{\mathrm{d}t} &= \alpha + (\eta + B)S - \mu D - UDR,\end{aligned}$$

where the capital letter X denotes the integral $r(X)$ of the kernel X, α is non-negative and all other parameters are positive. The two non-trivial equilibria are

$$\begin{aligned}\hat{P} &= \frac{\beta}{\gamma}\\ \hat{R} &= [a + b(c+d) - \xi] \cdot \frac{1}{2BU\kappa\gamma}\\ \hat{S} &= [a - b(c+d) + \xi] \cdot \frac{1}{2BU\eta\gamma}\\ \hat{D} &= [a + b(d-c) + \xi] \cdot \frac{1}{2U\eta\mu\gamma},\end{aligned}$$

where we abbreviate

$$\begin{aligned}a &= \beta BUG\\ b &= \gamma\eta\\ c &= \kappa\mu\\ d &= \alpha U\\ \xi &= \pm\sqrt{(a+b(c+d))^2-4abc}.\end{aligned}$$

Since we have $a, b, c > 0$ and $d \geq 0$, this yields that ξ is real, as

$$(a+b(c+d))^2-4abc = (a-bc)^2+bd(2a+(b(2c+d))) > 0.$$

Therefore, a unique positive equilibrium $\hat{S} > 0$ of satiated individuals exists when

$$a-b(c+d)\pm\sqrt{(a+b(c+d))^2-4abc} > 0.$$

If the third term has a negative sign, the condition cannot be satisfied. However, if the third term has a positive sign, then the condition is always satisfied if all parameters $a, b, c, d > 0$ are positive. In the case that $\alpha = 0$, that is, there is no immigration of individuals from outside the focal landscape, we have $d = 0$ and a positive equilibrium exists only if $a > bc$ holds. Rewriting this condition gives

$$\frac{G\beta}{\kappa\gamma} > \frac{\eta\mu}{BU},$$

where the left-hand side corresponds to the resource density of the habitat in the absence of the species. Thus, without outside immigration, the species cannot persist if the total amount and/or quality of habitat is not sufficiently high. In other words, we obtain an extinction threshold for the deterministic, non-spatial single-species model in the absence of outside immigration.

Comparison between the spatially-explicit stochastic model and the meanfield model. As the size parameter of the domain $V \to \infty$ goes to infinity, the stochastic model approaches the meanfield model in the limit of large-scale interactions (length scales $\ell \to \infty$). This is illustrated by Fig. S2.

B.3 Displacement of resource-deprived individuals

Expected number of jumps. A resource-deprived individual will die at rate μ and jumps at rate $r = r(M)$. Let s be the number of jumps a resource-deprived individual makes in its lifetime assuming it never becomes satiated (excluding the initial jump at birth). The number of jumps during an interval of length T is a random variable $X|T \sim \text{Poisson}(\lambda)$ with $\lambda = rT$. The length of the interval T is an exponentially distributed random variable $T \sim \text{Exp}(\mu)$. Thus,

$$\begin{aligned}
\Pr[s = k] &= \int_{t=0}^{\infty} \Pr[s = k | T = t] \cdot f(t; \mu) dt \\
&= \int_{t=0}^{\infty} e^{-rt} \frac{(rt)^k}{k!} \cdot f(t; \mu) dt \\
&= \int_{t=0}^{\infty} e^{-rt} \frac{(rt)^k}{k!} \cdot \mu e^{-\mu t} dt \\
&= \frac{\mu r^k}{(\mu + r)^{k+1}},
\end{aligned}$$

where $f(t; \mu)$ is the probability density function of the exponential distribution. The expected number $\mathrm{E}[s]$ of jumps is

$$\mathrm{E}[s] = \sum_{k=0}^{\infty} k \cdot \Pr[s = k] = \sum_{k=0}^{\infty} \frac{k \mu r^k}{(\mu + r)^{k+1}} = r/\mu.$$

Total displacement. The total displacement in coordinate i is a random variable

$$\sum_{j=0}^{s} x_{ij},$$

where $x_{ij} \sim \mathcal{N}(0, \delta^2/k)$ are independent Gaussian random variables (recall that x_{i0} is the initial displacement at birth). Since the expectation of x_{ij} is zero, the total displacement satisfies

$$\mathrm{E}\left[\sum_{j=0}^{s} x_{ij}\right] = (1 + \mathrm{E}[s]) \cdot 0 = 0 \qquad \text{and} \qquad \mathrm{Var}\left[\sum_{j=1}^{s} x_{ij}\right] = (1 + \mathrm{E}[s]) \cdot \delta^2/k = \delta^2,$$

as $\mathrm{E}[s] = r(M)/\mu = (k-1)\mu/\mu = k - 1$.

C Sensitivity of the simulation model and additional experiments

In this section, we provide additional experiments that show the model is robust to small changes to the assumptions and parameter values used. For example, we investigate the case of a completely neutral model (without environmental gradients in resource types), varying the number of species, and changing the size of the focal landscape. All variants provide qualitatively similar results. We start by showing that the simulations times are sufficient to reach a stationary state.

C.1 Convergence to the stationary state

All the simulation experiments were conducted with a simulation time $T = 400$ time units. Fig. S3 shows that on average the density of resource-satiated individuals quickly converges towards the stationary state well before 400 time units.

C.2 A neutral model with no variation in resource types

We considered a completely neutral variant of the model, where all the species compete on the same limiting resource. The patterns produced are similar to the ones produced by the non-neutral model with large-scale environmental variation in resource types. In the neutral setting, the all habitat areas produce the same type of resource. This yields that the effect of habitat loss on species richness is not as pronounced. For example, Fig. S6 does not show the downward slope of the unimodal curve in the second and third columns.

C.3 Sensitivity to the number of species

We repeated the main experiments underlying with 32 and 64 species (in contrast to the 128 species used in the main text). Irrespective of the number of species, the analyses yield qualitatively similar results (i.e. at high amount of total habitat fragmentation has positive effect, but at smaller amount of total habitat high levels of fragmentation tend have a negative effect on species richness):

- Results for a species pool of size $S = 64$:
  - neutral community: Fig. S7, Fig. S8, Fig. S9, Fig. S10, Fig. S11
  - environmental gradient: Fig. S12, Fig. S13 and Fig. S14, Fig. S15, Fig. S16.
- Results for a species pool of size $S = 32$:
  - neutral community: Fig. S17, Fig. S18 and Fig. S19,

- environmental gradient: Fig. S22, Fig. S23 and Fig. S24,

C.4 Sensitivity to the size of the landscape

The main experiments have been conducted in a landscape of size 100 × 100. We repeated the experiments in smaller landscapes of size 25 × 25. Figures S27, S28, and S29 show that in these smaller landscapes the SLOSS and SFAR analyses provide qualitatively same results, but the threshold at which fragmentation effects appear is higher (as there is less absolute amount of habitat available).

C.5 Testing the sample area effect with larger sampling sites

We repeated the analysis of the sample area effect using larger sampling sites (i.e. we increased the radius τ of sampling sites from 1 to 2). The results of the sample area effect analysis with a larger sampling radius $\tau = 2$ are given in Table S2 and Fig. S33.

	δ	β_L (M_{L+F})	β_F (M_{L+F})	β_L (M_L)	β_F (M_F)	AIC M_{L+F}	AIC M_L	AIC M_F	AIC M_{null}
H	1	0.31	0.01	0.34	0.09	0	0.73	206.61	631.67
H	3	0.35	0.03	0.41	0.16	0	21.96	567.51	2686.65
H	10	0.39	0.04	0.44	0.23	0	41.67	1376.99	5317.45
P	1	0.3	0.02	0.35	0.09	0	5.51	185.89	654.47
P	3	0.34	0.05	0.44	0.16	0	56.92	477.59	2607.98
P	10	0.39	0.03	0.43	0.22	0	23.07	1601.34	5256.4
A	1	0.26	0.02	0.32	0.1	0	11.71	230.15	1024.24
A	3	0.28	0.01	0.29	0.13	0	0.39	583.72	2255.06
A	10	0.31	-0.01	0.3	0.15	0	3.2	1719.37	3863.53

Table S2: Results for the HAH test in landscapes with sampling site radius $\tau = 2$

	δ	β_L (M_{L+F})	β_F (M_{L+F})	β_L (M_L)	β_F (M_F)	AIC M_{L+F}	AIC M_L	AIC M_F	AIC M_{null}
H	1	0.22	0.03	0.28	0.1	0	11.11	99.28	550.35
H	3	0.21	0	0.22	0.12	1.82	0	194.09	1072.13
H	10	0.2	0	0.2	0.13	2	0	306.7	1239.33
P	1	0.27	0.02	0.31	0.1	0	2.47	152.7	623.36
P	3	0.23	0	0.23	0.11	1.99	0	189.4	911.92
P	10	0.18	0.03	0.21	0.14	0	8.82	197.53	1297.67
A	1	0.2	0.02	0.23	0.1	0	2.61	108.59	627.28
A	3	0.14	0.02	0.17	0.09	0	3.17	87.91	673.77
A	10	0.11	0.02	0.13	0.09	0	3.78	141.05	638.52

Table S3: Results for the HAH test in neutral communities of 128 species, sampling site radius $\tau = 1$

	δ	β_L (M_{L+F})	β_F (M_{L+F})	β_L (M_L)	β_F (M_F)	AIC M_{L+F}	AIC M_L	AIC M_F	AIC M_{null}
H	1	0.23	0.02	0.28	0.09	0	8.69	114.14	543.32
H	3	0.23	0	0.23	0.11	1.7	0	297.11	1281.35
H	10	0.18	0.01	0.2	0.12	0	2	492.31	1803.83
P	1	0.26	0.01	0.28	0.08	0.41	0	152.08	525.55
P	3	0.23	0	0.22	0.11	1.66	0	287.01	1236.04
P	10	0.18	0.02	0.21	0.12	0	5.79	316.61	1683.08
A	1	0.23	0.02	0.26	0.1	0	2.2	172.13	788.28
A	3	0.14	0.01	0.16	0.09	0	0.7	142.63	865.35
A	10	0.14	-0.01	0.13	0.07	0	1.52	410.19	981.85

Table S4: Results for the HAH test in neutral communities of 128 species, sampling site radius $\tau = 2$

C.6 An alternative parameterisation of the individual-based model

We also considered a parameterisation, where the species were more sensitive to resource availability, but in contrast had increased birth rates. Specifically, we changed the parameterisation as follows:

- resource units disappear faster when left unconsumed (κ was increased from 0.1 to 0.2),
- resource-satiated individuals become resource deprived faster (η increased from 0.2 to 0.5),
- resource-satiated individuals produce more propagules (birth rate B increased from 1 to 2).

Here, we used a species pool of $S = 64$ species. We repeated the SFAR and SLOSS analyses for this parameterisation of the model with and without large-scale environmental gradients. We observed that the species communities became more sensitive to loss of habitat but also to fragmentation. The experiments are summarised in the following figures:

- SLOSS analysis *without* environmental gradient Fig. S34,
- SLOSS analysis *with* environmental gradient Fig. S37,
- SFAR analysis *without* environmental gradient Fig. S35 and Fig. S36,
- SFAR analysis *with* environmental gradient Fig. S38 and Fig. S39.

The SLOSS analyses show negative responses to fragmentation, particularly in the case with environmental gradients in habitat types (Fig. S34 and Fig. S37). We see that in both cases, the species become more sensitive to habitat loss, as the SFAR curves have steeper slopes (Fig. S35 and Fig. S38). Moreover, we see that species richness responds to fragmentation per se in a non-monotone way, but it is predominantly much less positive than in the prior parameterisation ((Fig. S36 and Fig. S39). We note that increasing fragmentation had little to no effect when the total amount of habitat was high (32% habitat cover), but in the alternative parameterisation with an environmental gradient

also these high levels of total habitat showed a non-monotone response to fragmentation (top lines in Fig. S39).

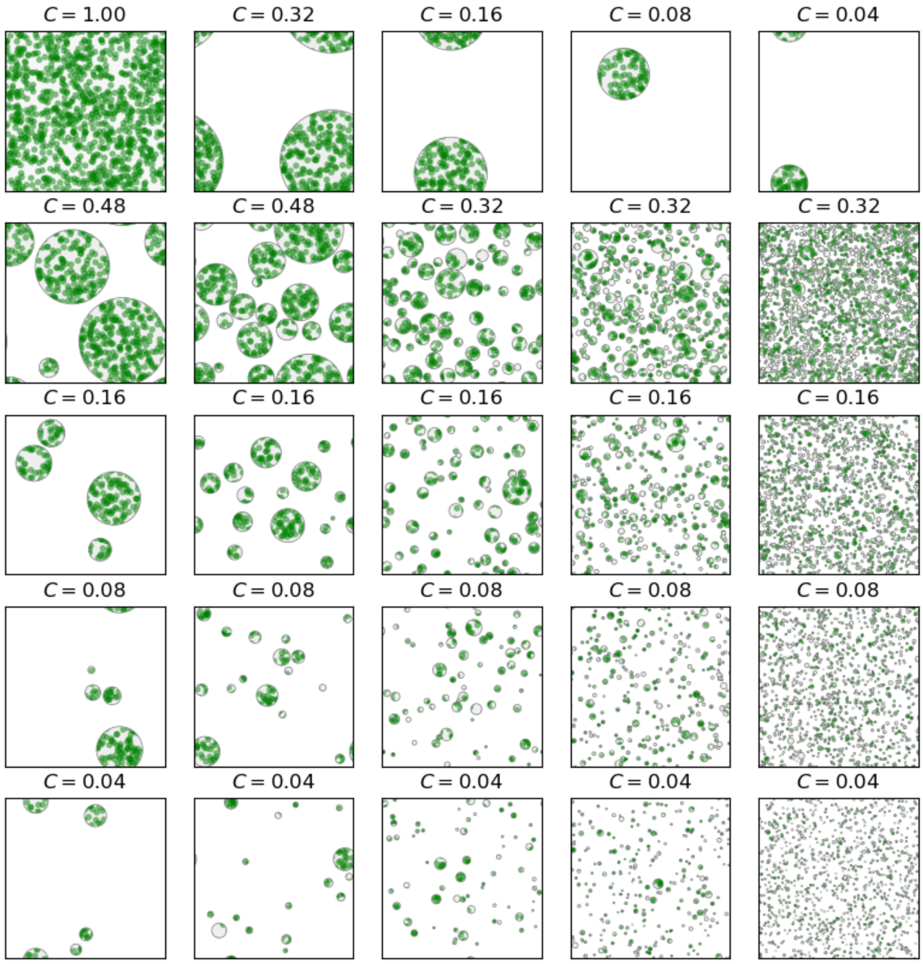

Figure S1: Examples of fragmented landscapes with varying levels of fragmentation and habitat cover. The landscape is a torus (i.e. periodic boundaries) of size 100×100. Green cirles are resource patches, which may overlap to create higher resource production rates at some areas (darker green). The filled gray circles represent habitat fragments within which resource patches follow stochastic birth-death dynamics, and within these circles, the gray area represents habitat (currently) without resource patches. The white areas represent the matrix, that is, area which has zero resource production rate, as resource units cannot establish in the matrix. The habitat fragments do not overlap.

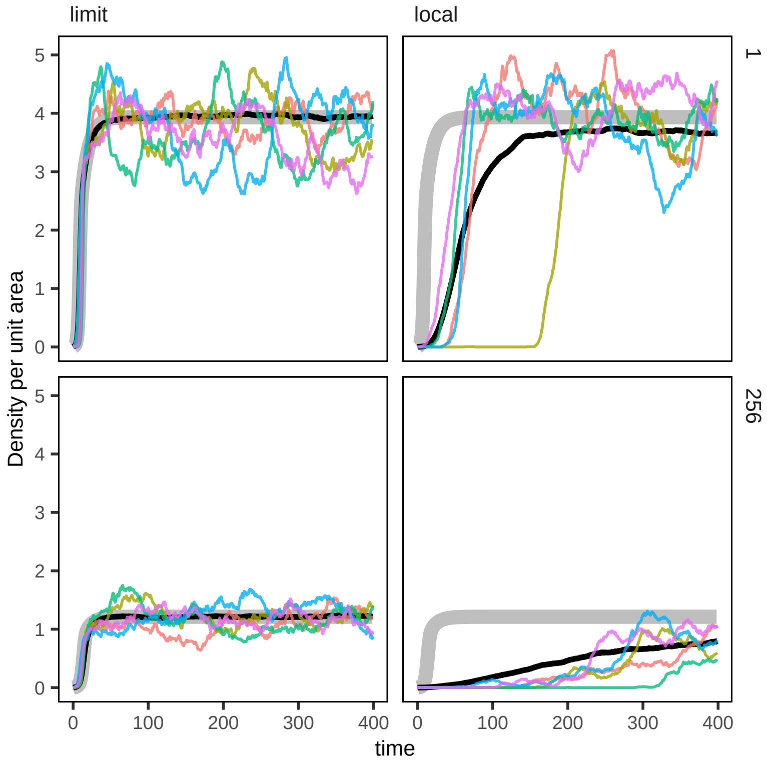

Figure S2: Comparison between the spatial stochastic model with a single species and the meanfield model. Here, to exaggarate the difference between the spatial and meanfield models, we have used a parameterization which differs from the parameterisation given in Table S1 as follows: the patch birth and turnover rates are $\beta = 0.05$ and $\gamma = 0.025$, resource production rate is 2 (rate of G), resource deprivation rate is $\eta = 2$, and birth rate is 1.5 (rate of B). The spatial domain of the simulation is 15×15. The grey line is the equilibrium solution $\hat{S}$ for the density of resource-satiated individuals, the coloured lines are example trajectories of the per unit area densities of resource satiated individuals in the stochastic model, and the black line gives the average density over 200 replicate trajectories. The column “limit“ considers simulations with length scales of all the kernels at $V/2$, whereas “local” has the length scales as before. The top row considers a scenario with completely intact landscape, whereas the bottom row considers a scenario with a landscape that has been fragmented to 256 habitat fragments that cover 16% of the simulation area.

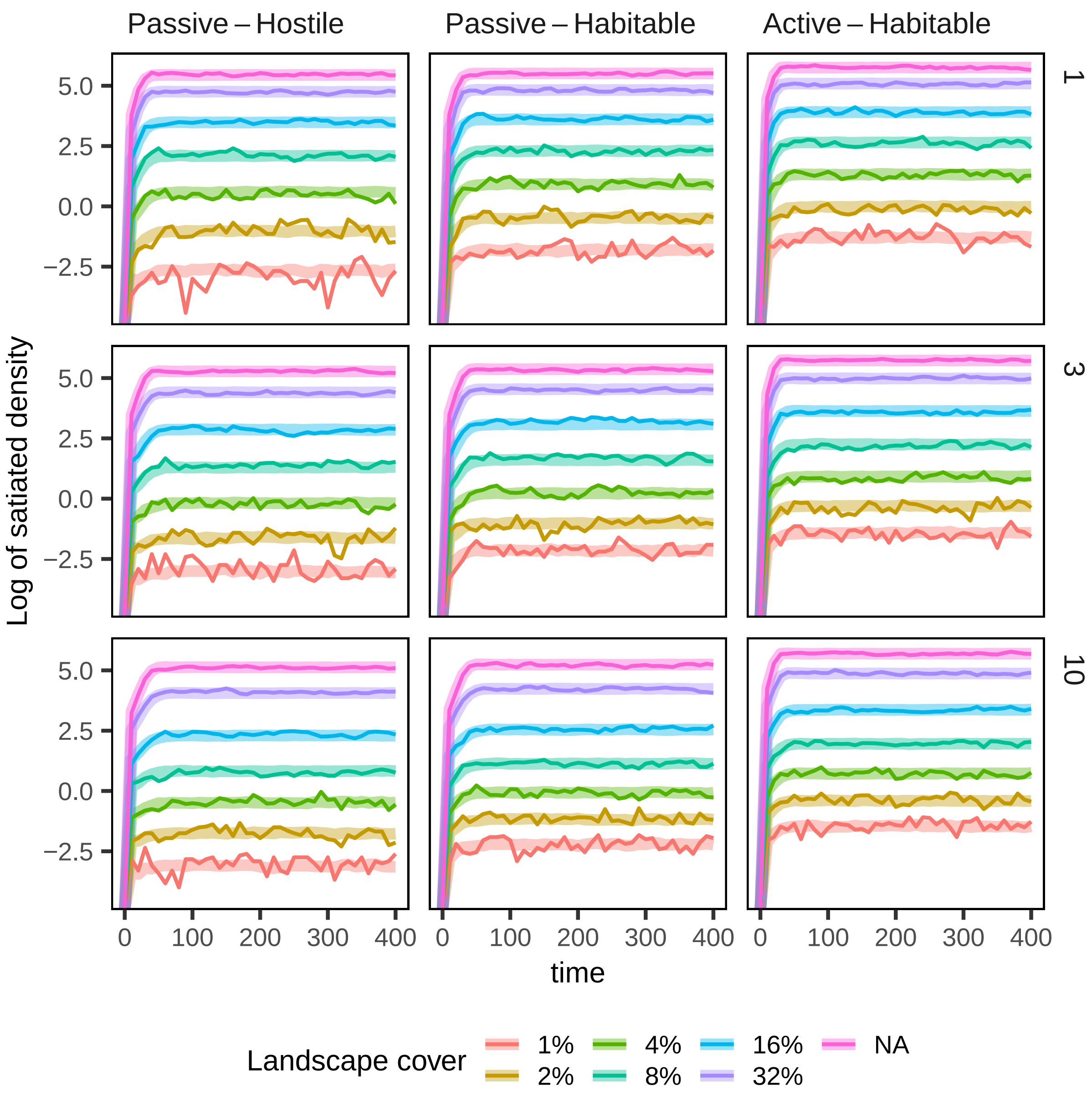

Figure S3: The average density of resource-satiated individuals over 100 replicate landscapes. The rows denote different values of the dispersal parameter δ and each columns considers a different dispersal/matrix scenario. In each panel, the thick lines give the average density of resource-satiated individuals across all simulation replicates as a function of time. The thin lines give the average density of resource-satiated individuals in a single replicate landscape. The figure shows the results for experiments where the focal landscapes have size 100×100 and which have been fragmented into 256 parts.

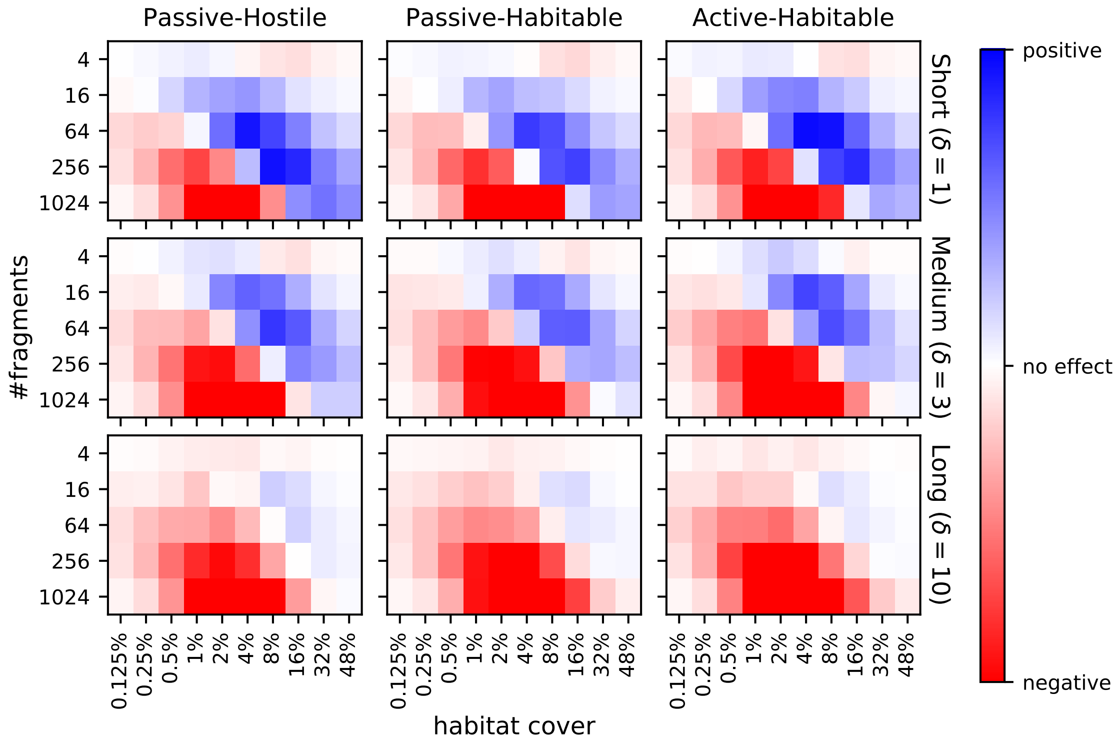

Figure S4: SLOSS analysis on a neutral community with 128 species.

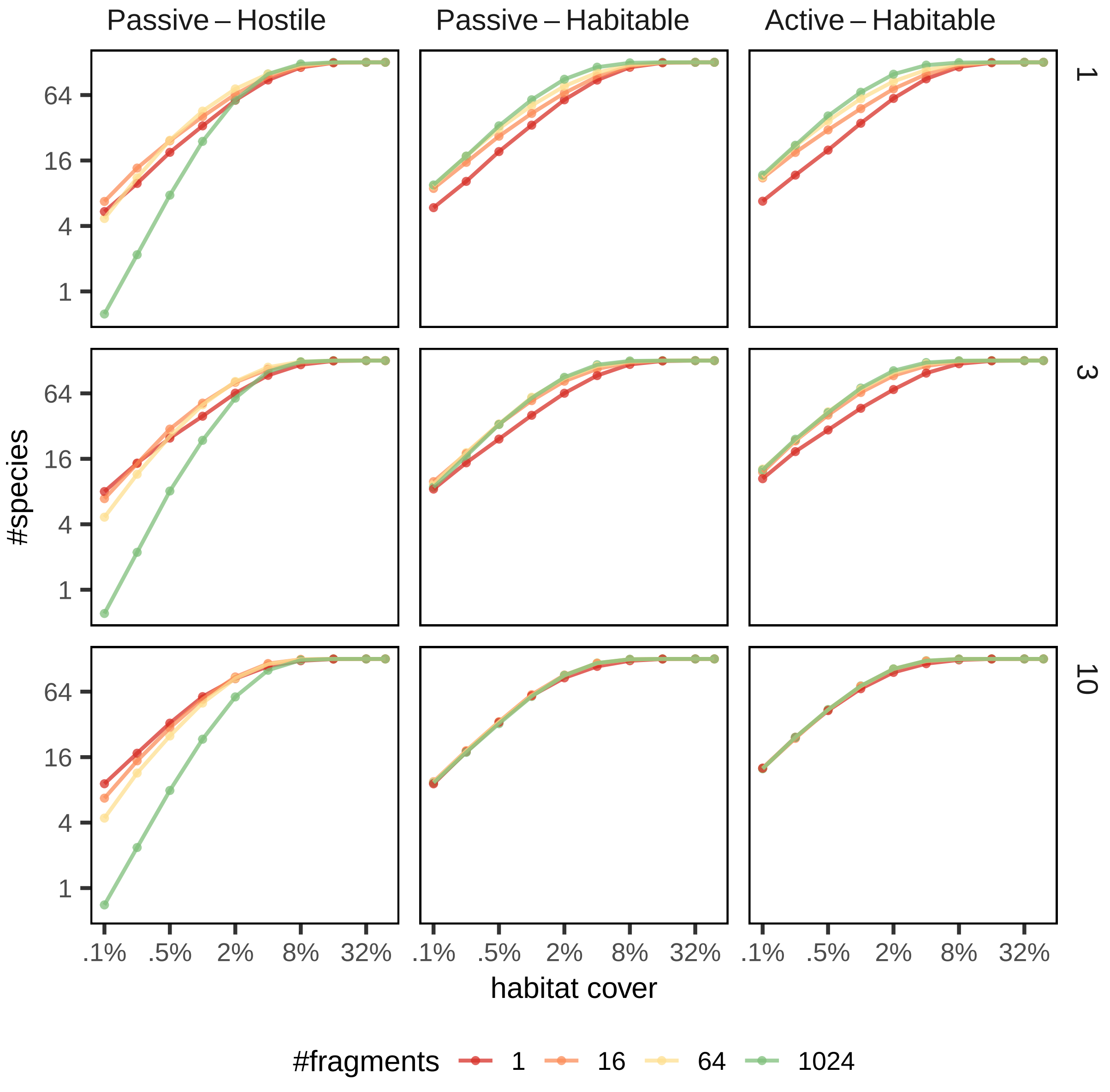

Figure S5: SFAR analysis on a neutral community with 128 species.

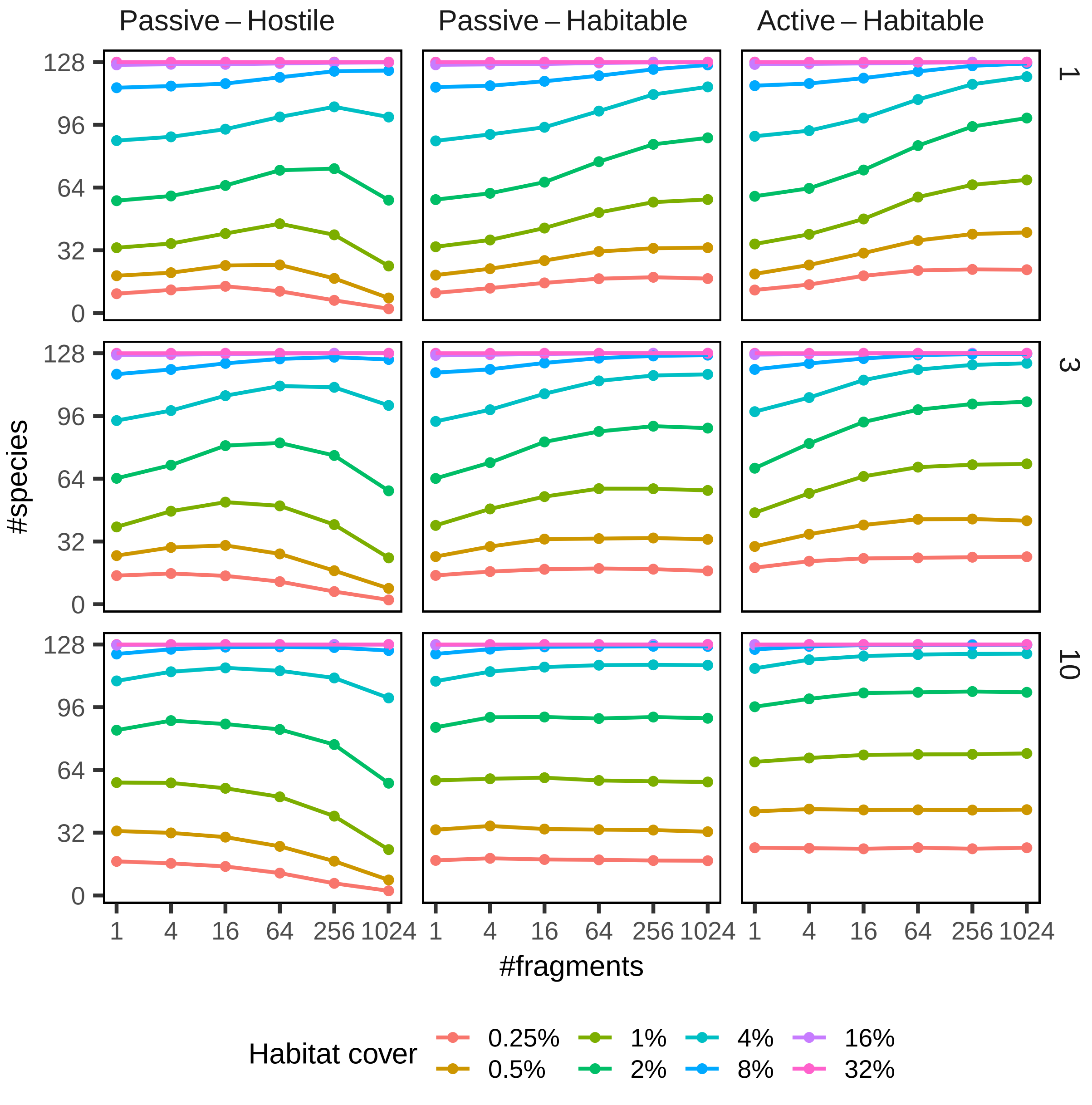

Figure S6: Species richness as a function of habitat fragmentation in a community with 128 species.

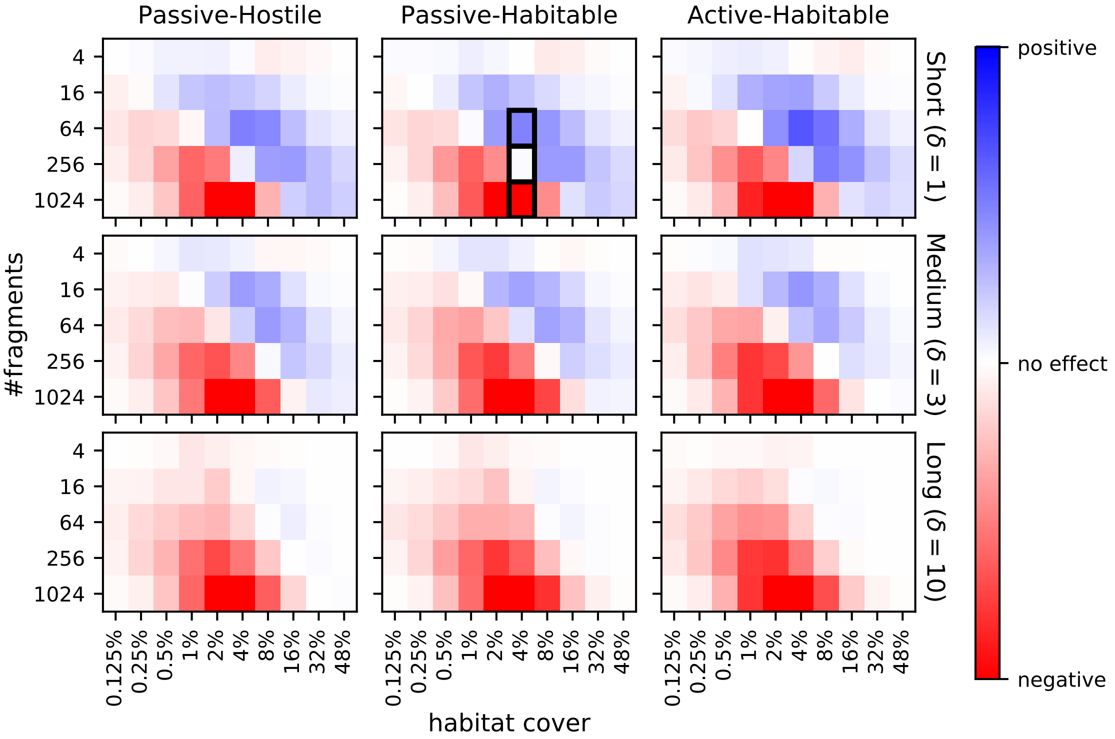

Figure S7: SLOSS analysis of the neutral community of 64 species in a 100 × 100 landscape.

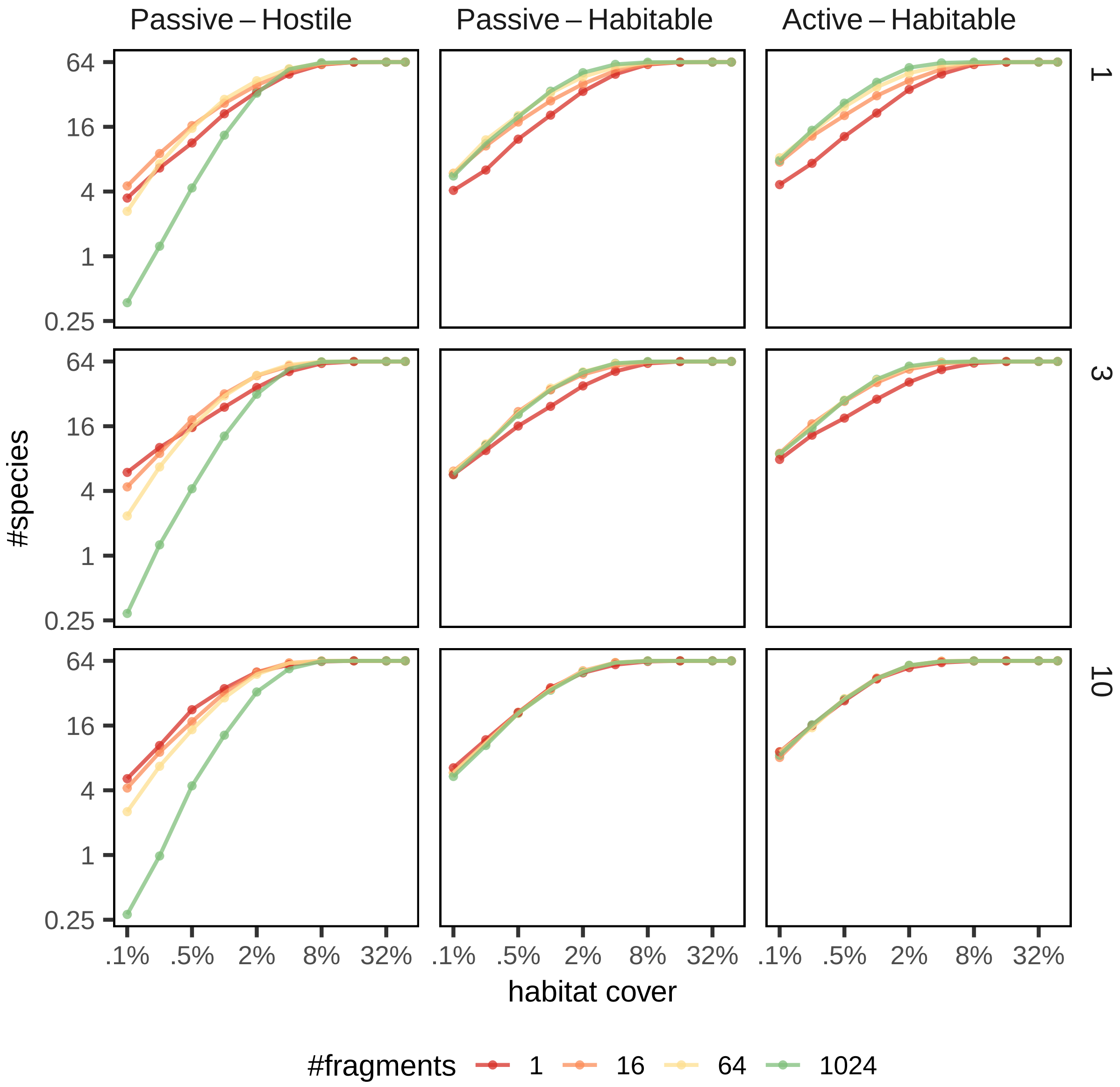

Figure S8: SFAR analysis on the neutral community of 64 species in a 100 × 100 landscape.

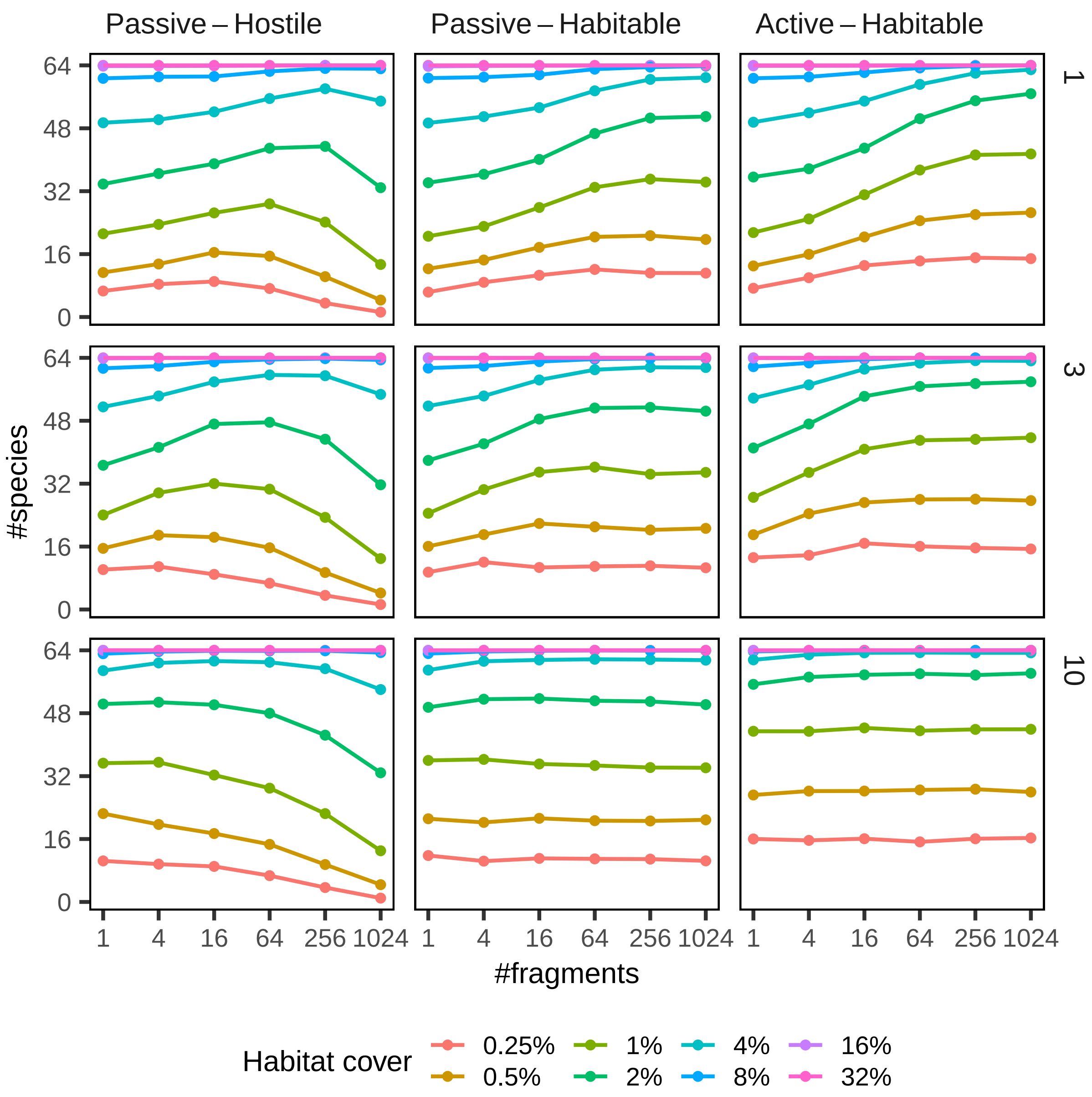

Figure S9: Species richness as a function of habitat fragmentation in a neutral community of 64 species in a 100×100 landscape.

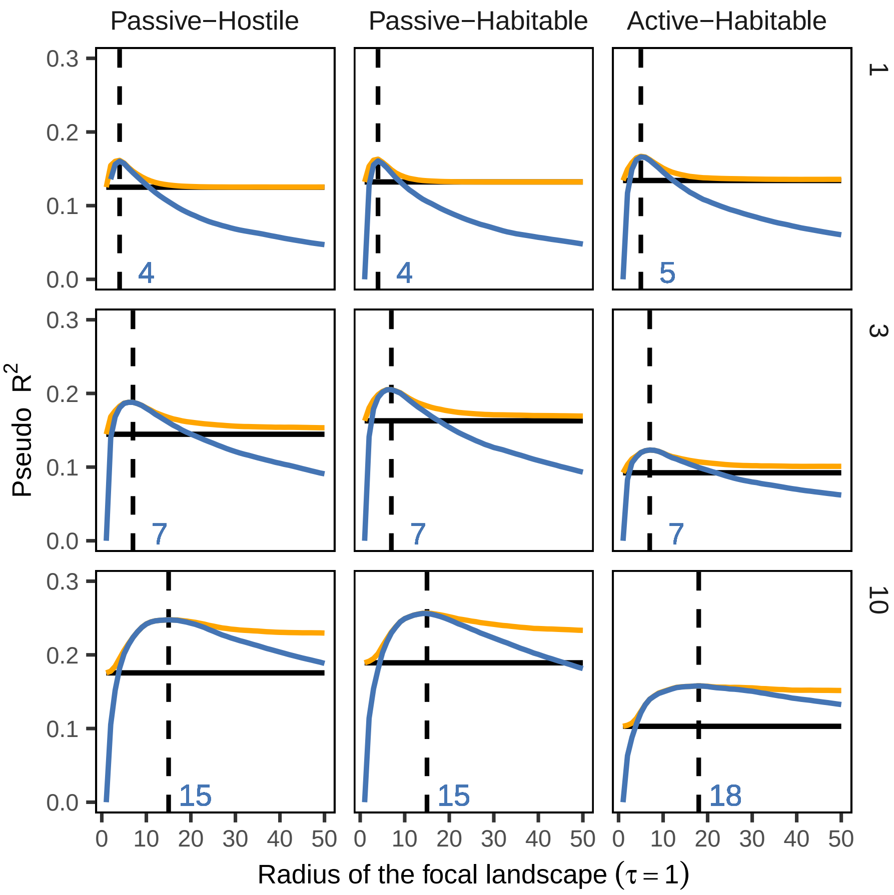

Figure S10: Inferring the appropriate scale of local landscape and testing the sample area effect on a neutral community of size 32. The radius of the sample site is $\tau = 1$.

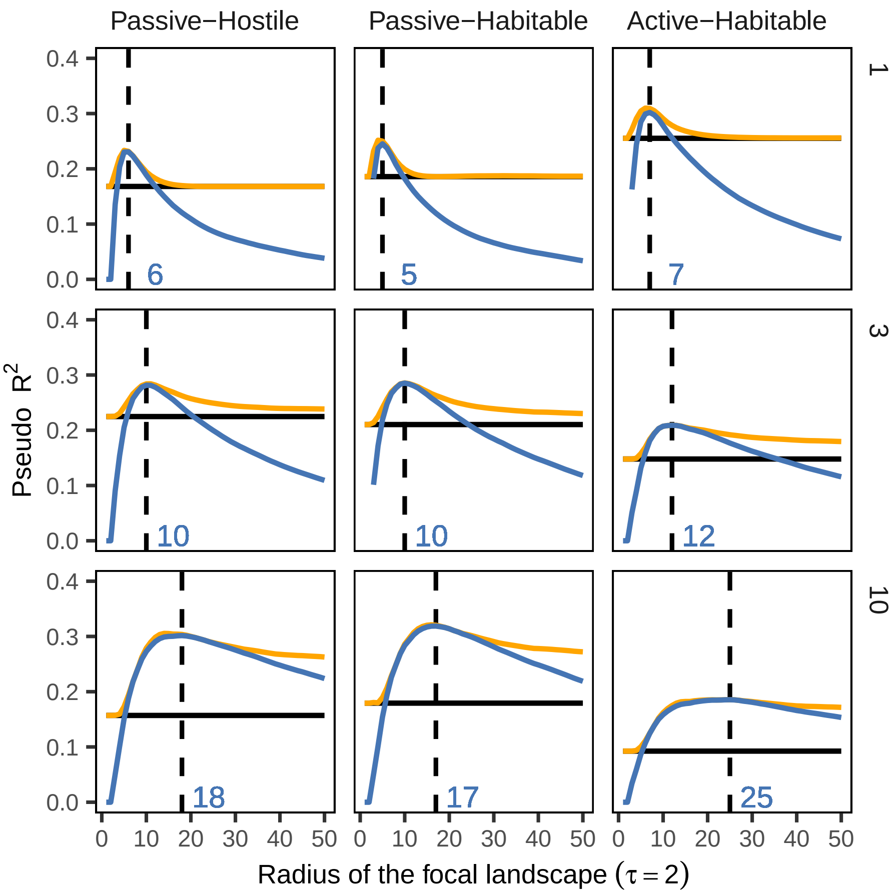

Figure S11: Inferring the appropriate scale of local landscape and testing the sample area effect on a neutral community of size 32. The radius of the sample site is $\tau = 2$.

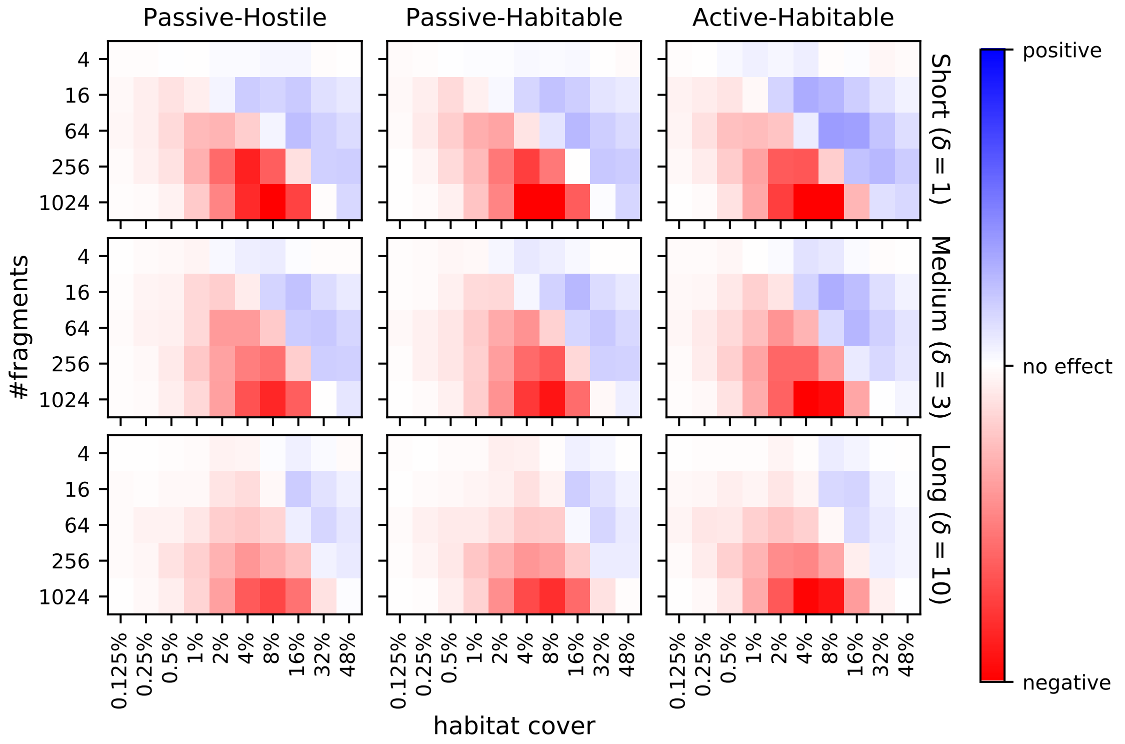

Figure S12: SLOSS analysis on the community of 64 species in a 100 × 100 landscape with an environmental gradient.

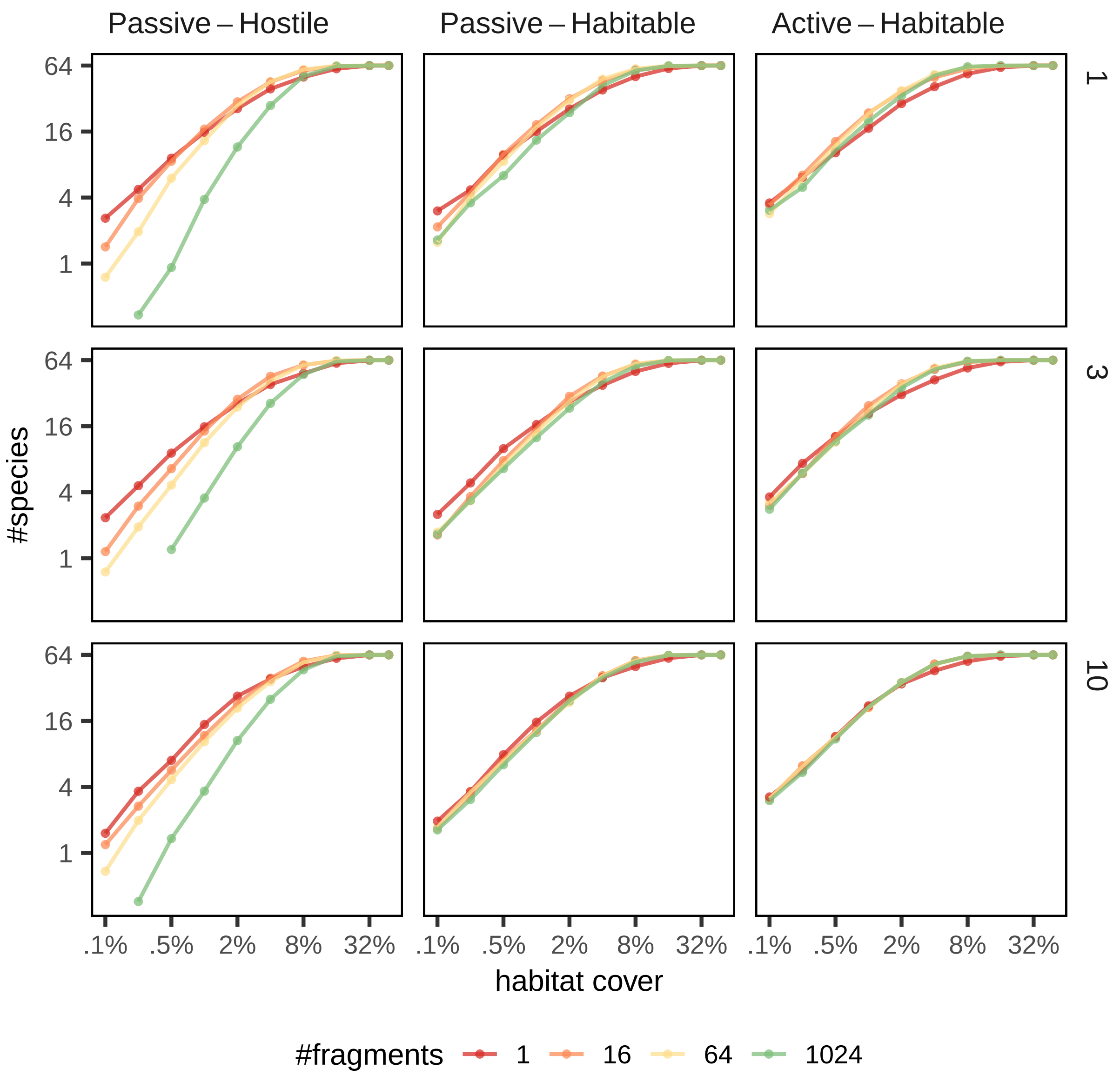

Figure S13: SFAR analysis on the community of 64 species in a 100 × 100 landscape with an environmental gradient.

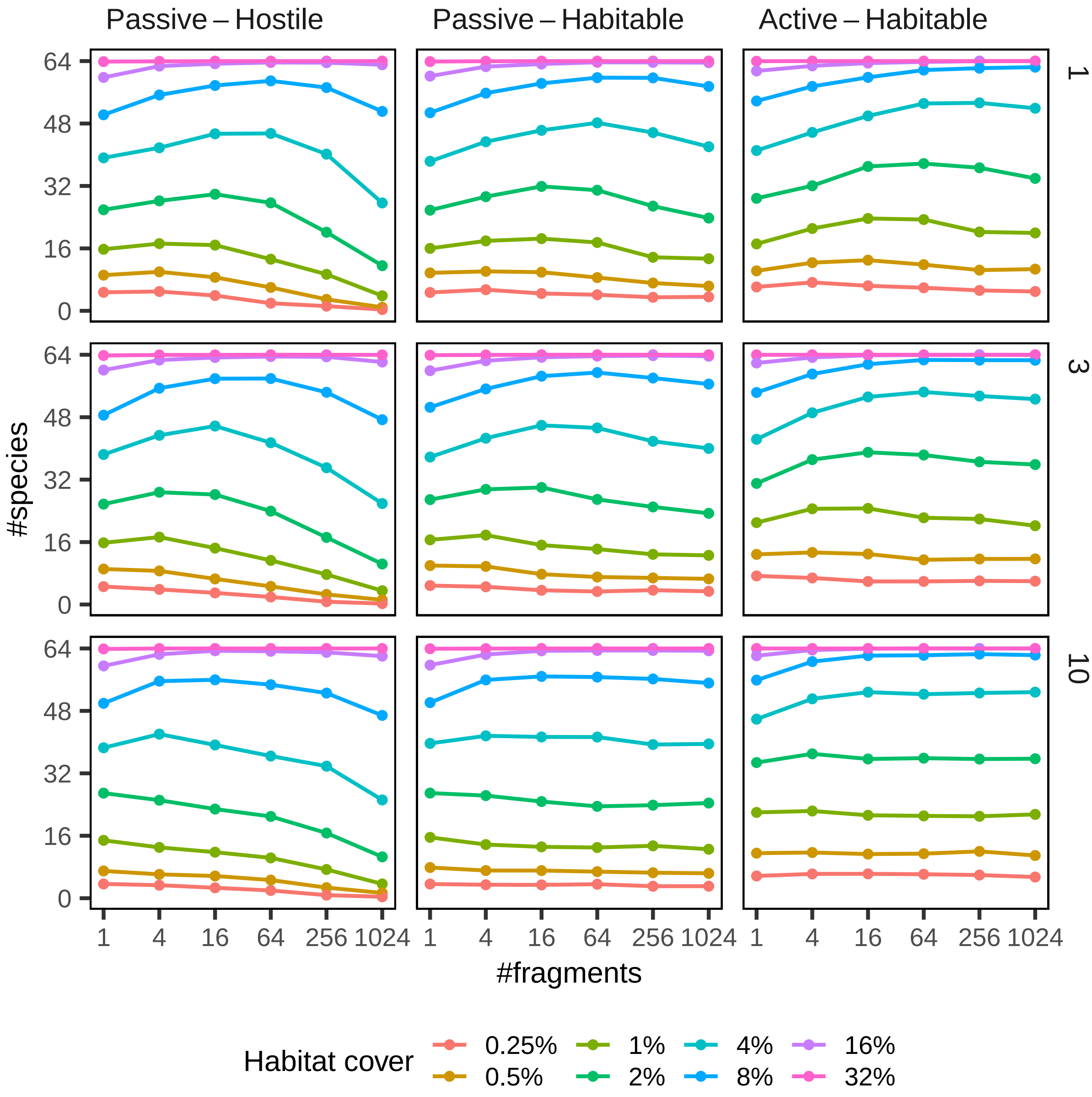

Figure S14: Species richness as a function of habitat fragmentation in a community of 64 species in a 100×100 landscape with an environmental gradient.

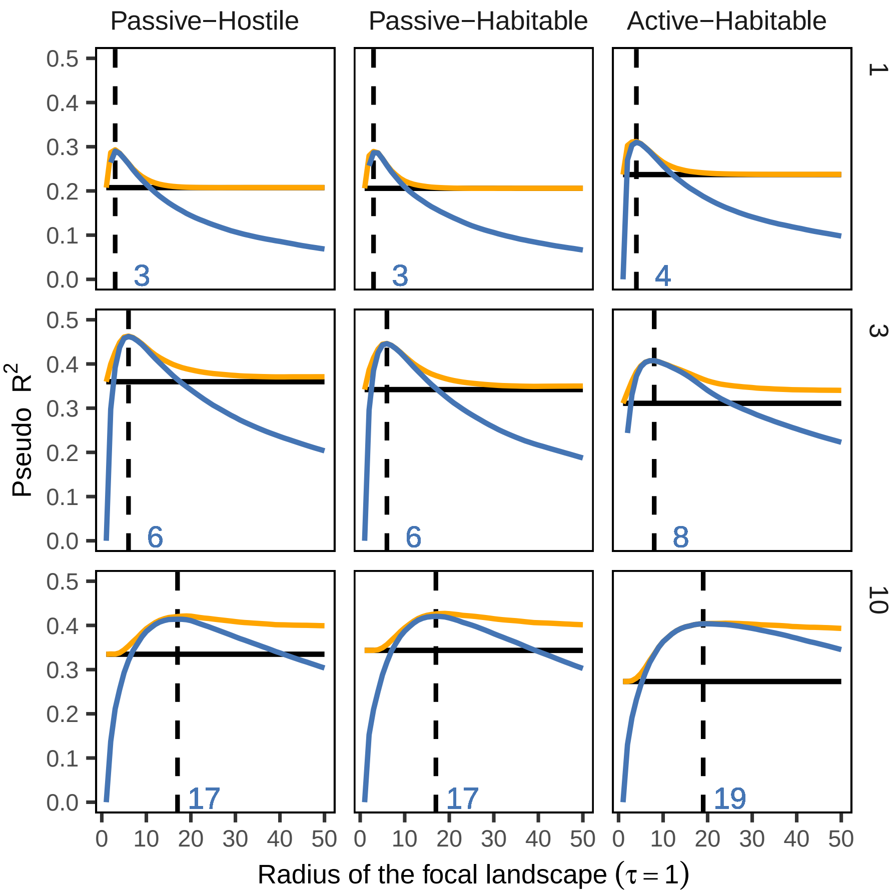

Figure S15: Inferring the appropriate scale of local landscape and testing the sample area effect on a community of size 64 with an environmental gradient. The radius of the sample site is $\tau = 1$.

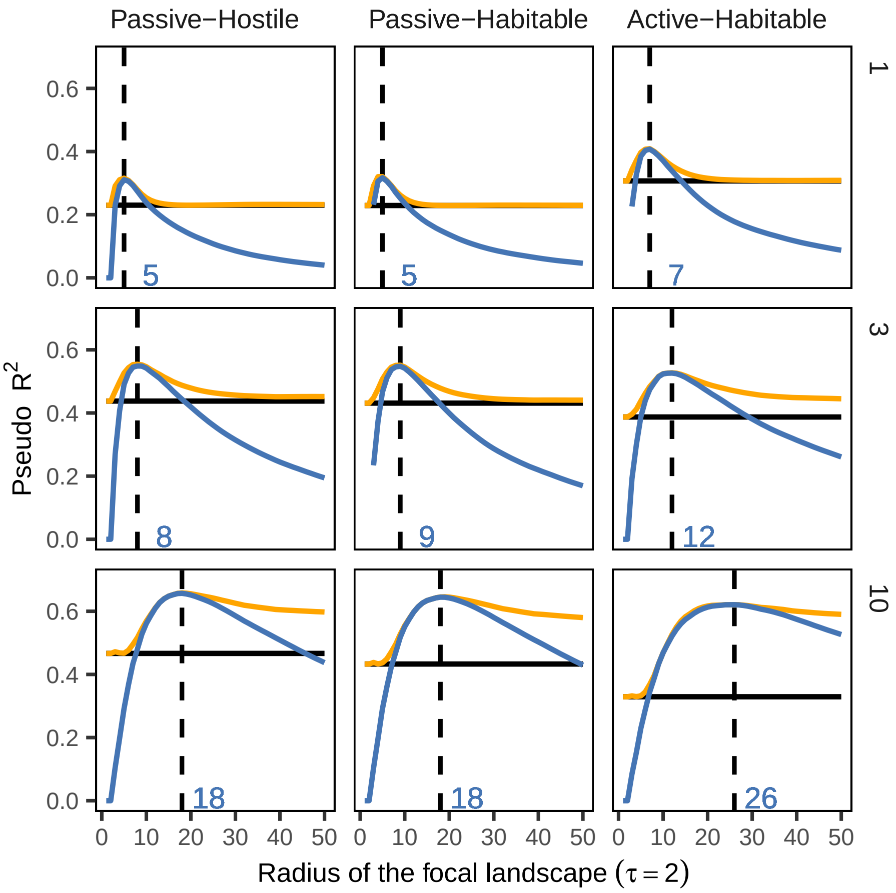

Figure S16: Inferring the appropriate scale of local landscape and testing the sample area effect on a neutral community of size 32. The radius of the sample site is $\tau = 2$.

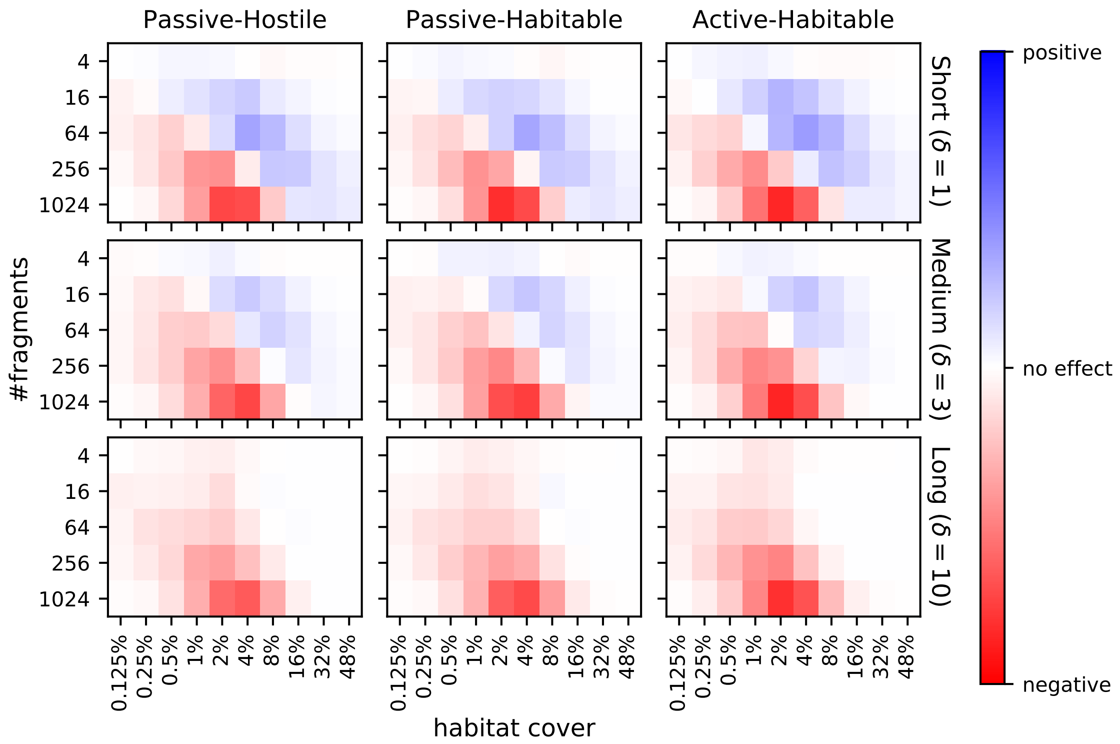

Figure S17: SLOSS analysis of the neutral community of size 32 species in a 100 × 100 landscape.

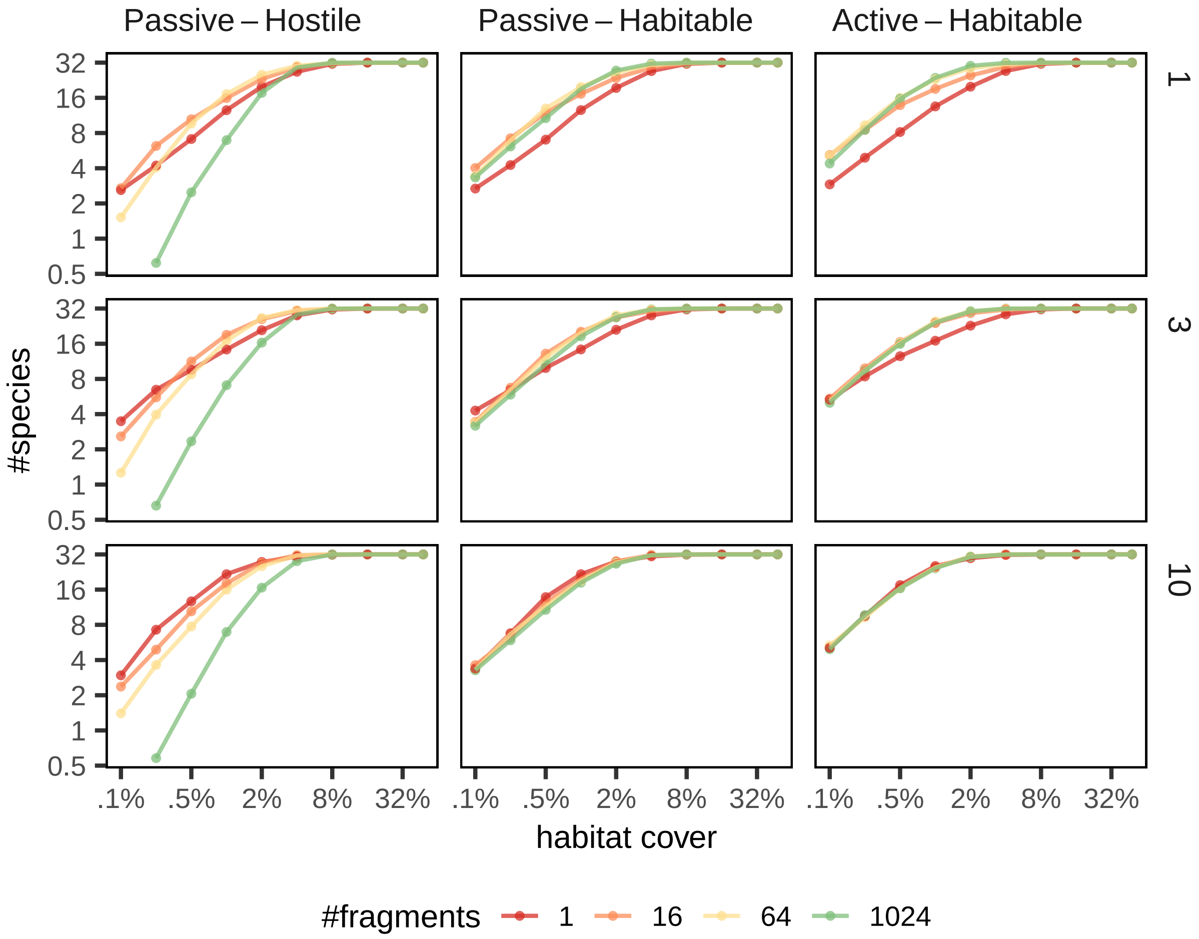

Figure S18: SFAR analysis on the neutral community of size 32 species in a 100 × 100 landscape.

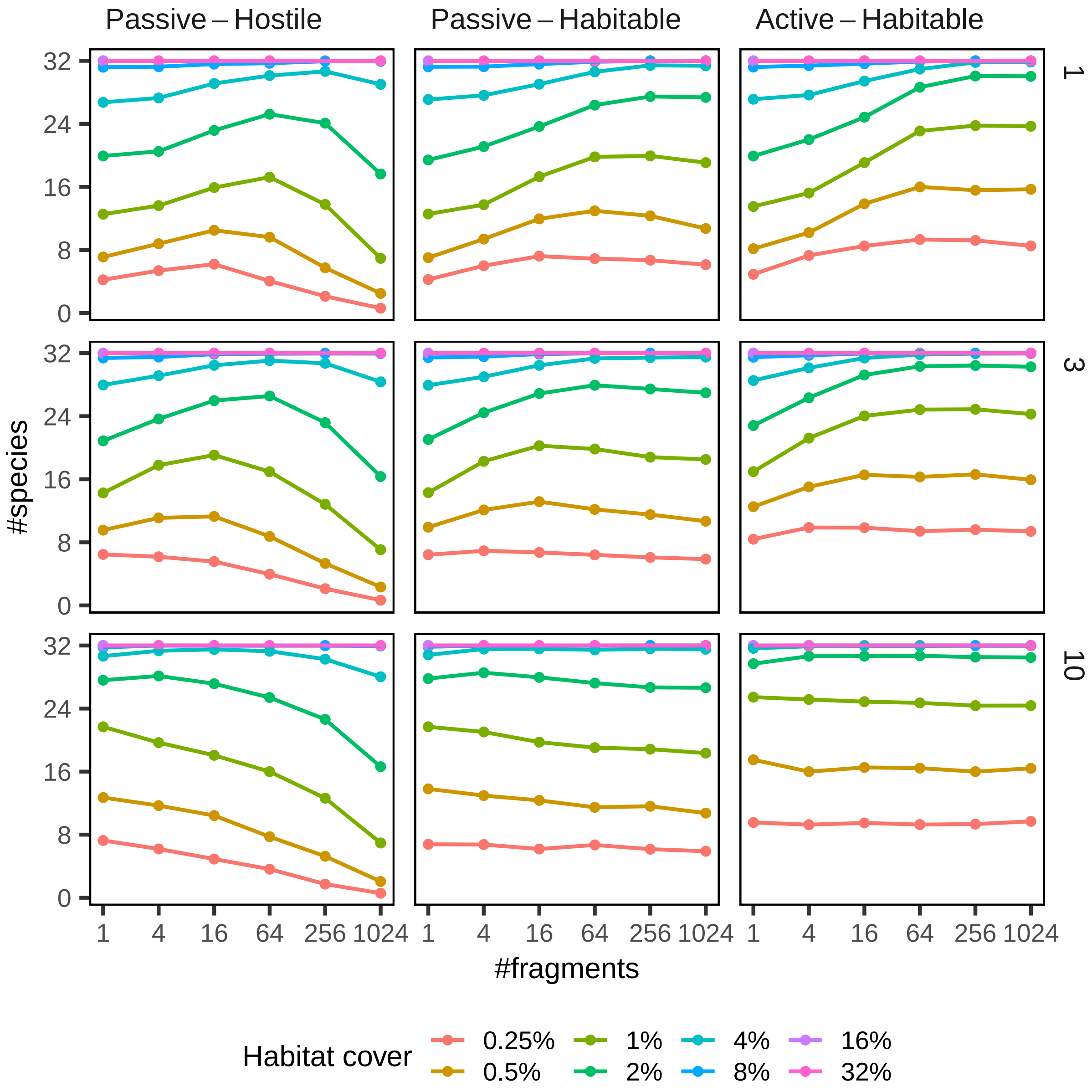

Figure S19: Species richness as a function of habitat fragmentation in a neutral community of 32 species.

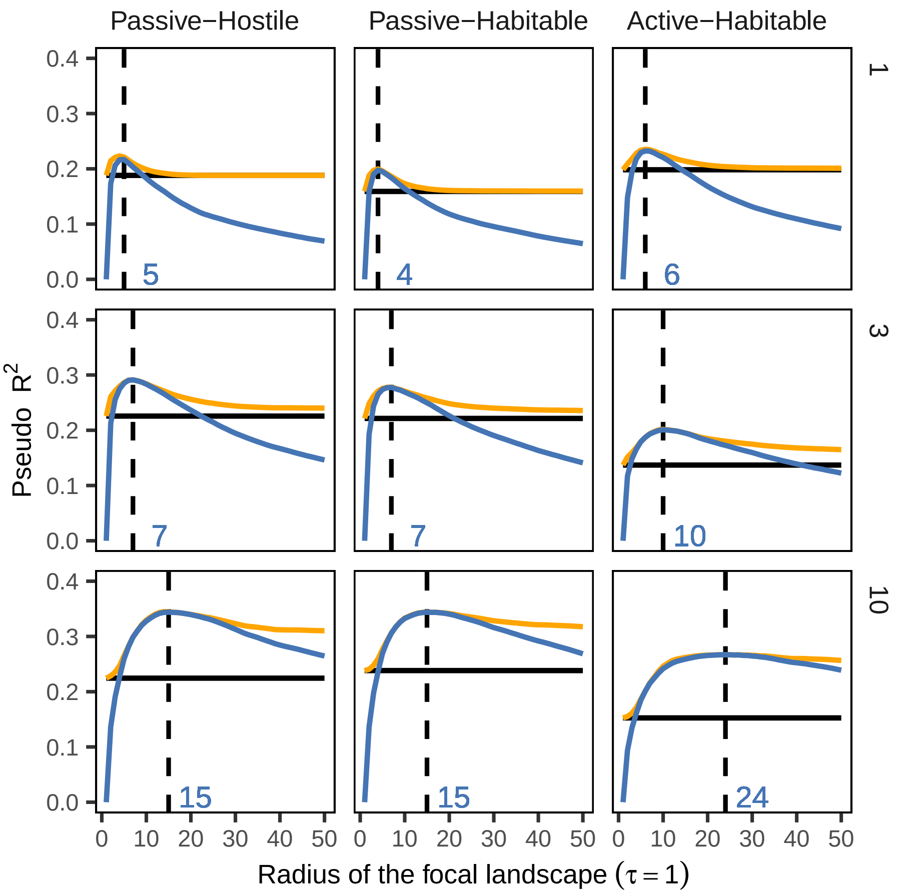

Figure S20: Inferring the appropriate scale of local landscape and testing the sample area effect on a neutral community of size 32. The radius of the sample site is $\tau = 1$.

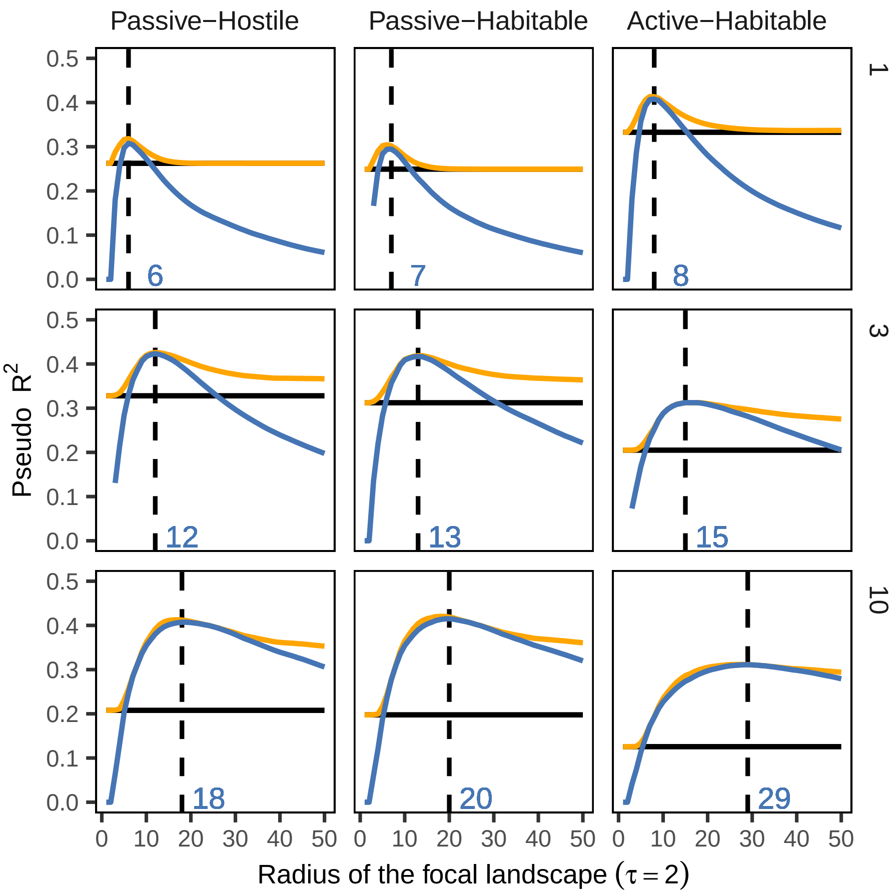

Figure S21: Inferring the appropriate scale of local landscape and testing the sample area effect on a neutral community of size 32. The radius of the sample site is $\tau = 2$.

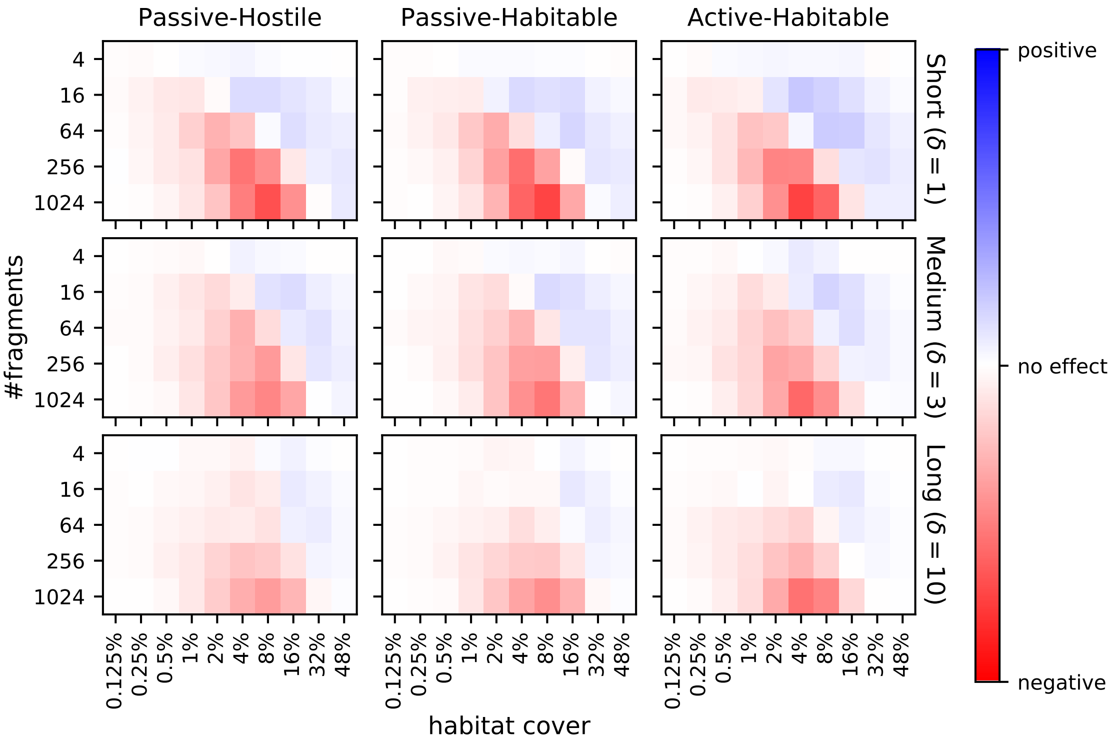

Figure S22: SLOSS analysis of a community of size 32 species in a 100 × 100 landscape with an environmental gradient.

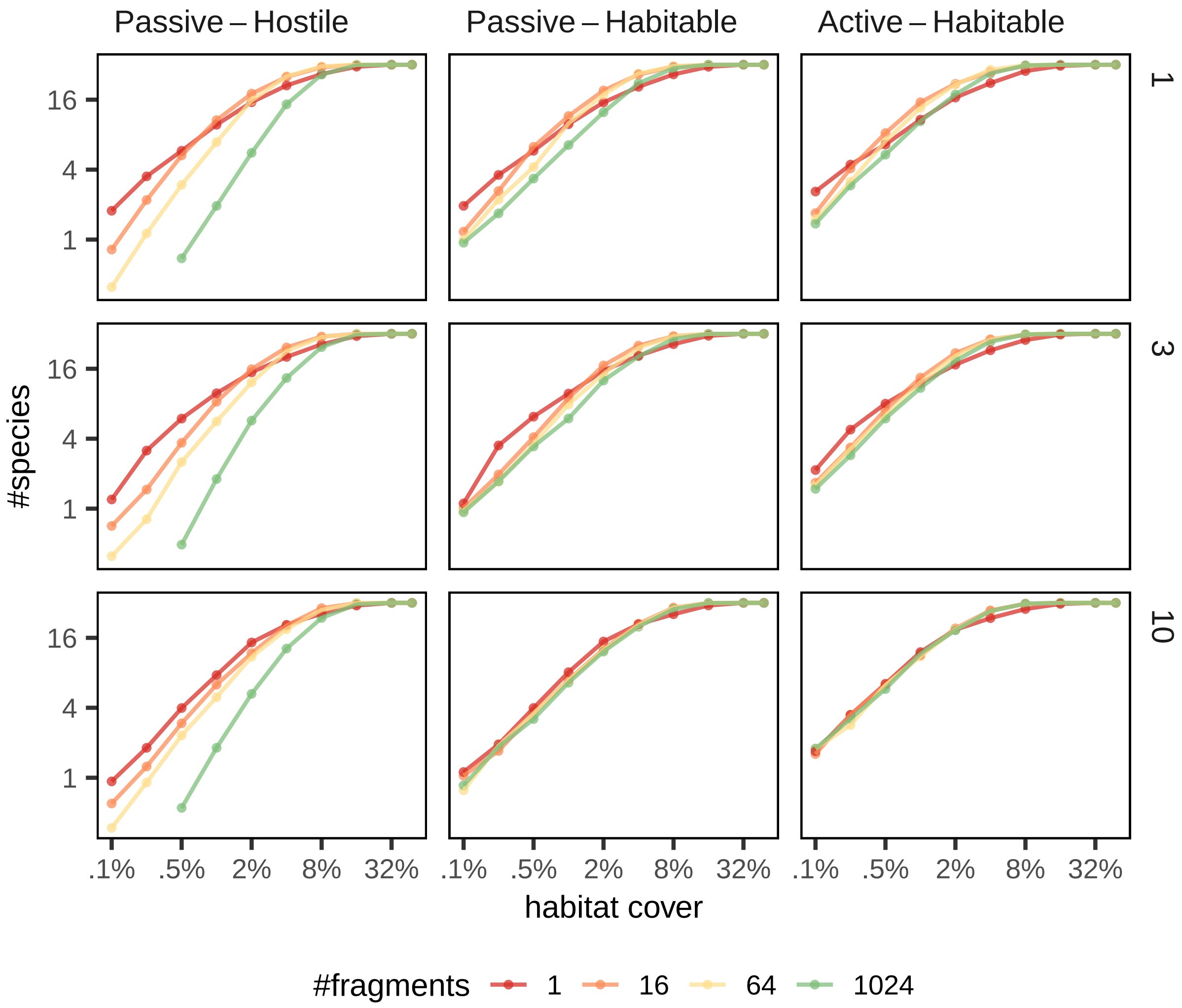

Figure S23: SFAR analysis on the community of size 32 species in a 100 × 100 landscape with an environmental gradient.

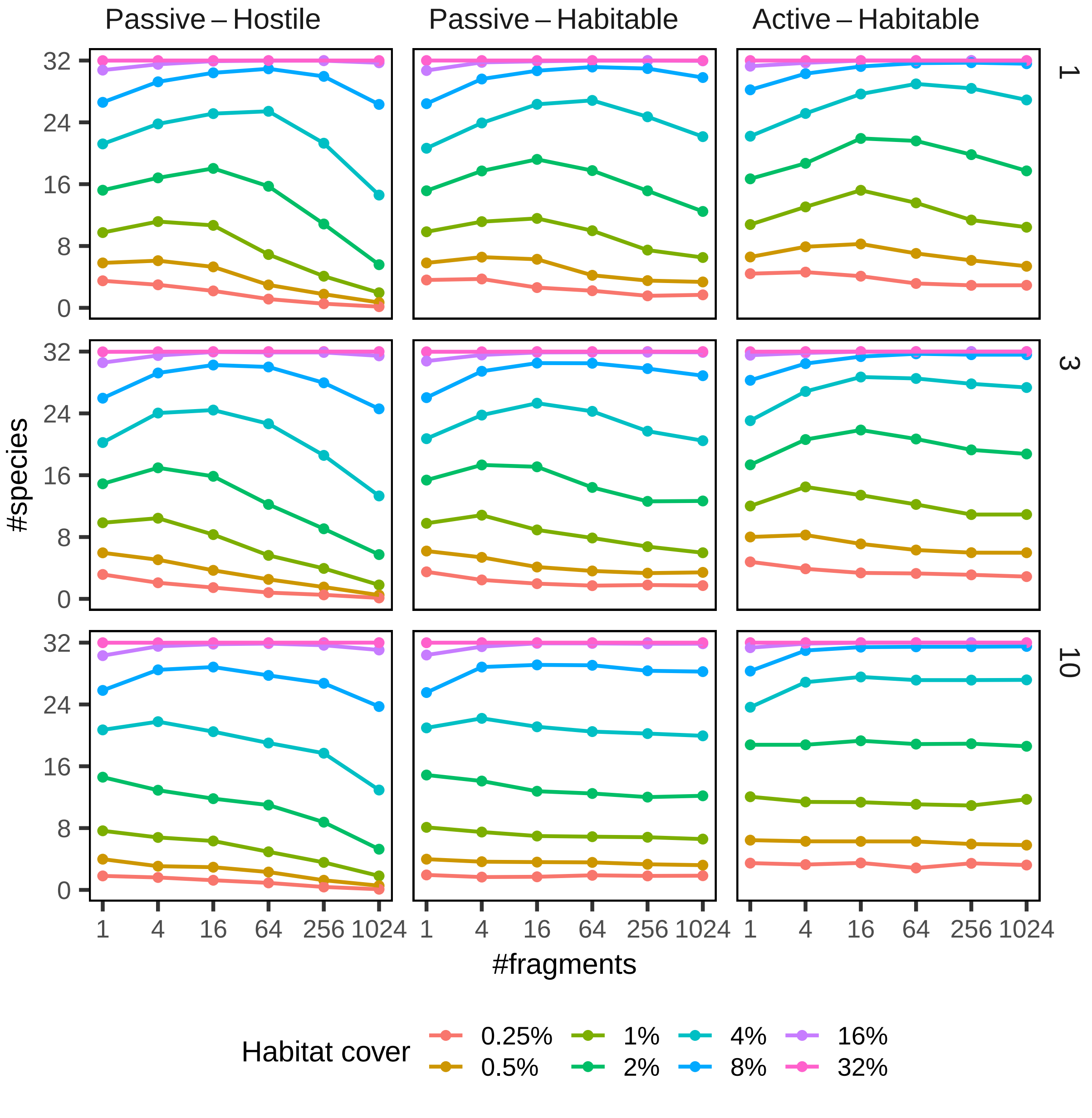

Figure S24: Species richness as a function of habitat fragmentation in a community of 32 species with an environmental gradient.

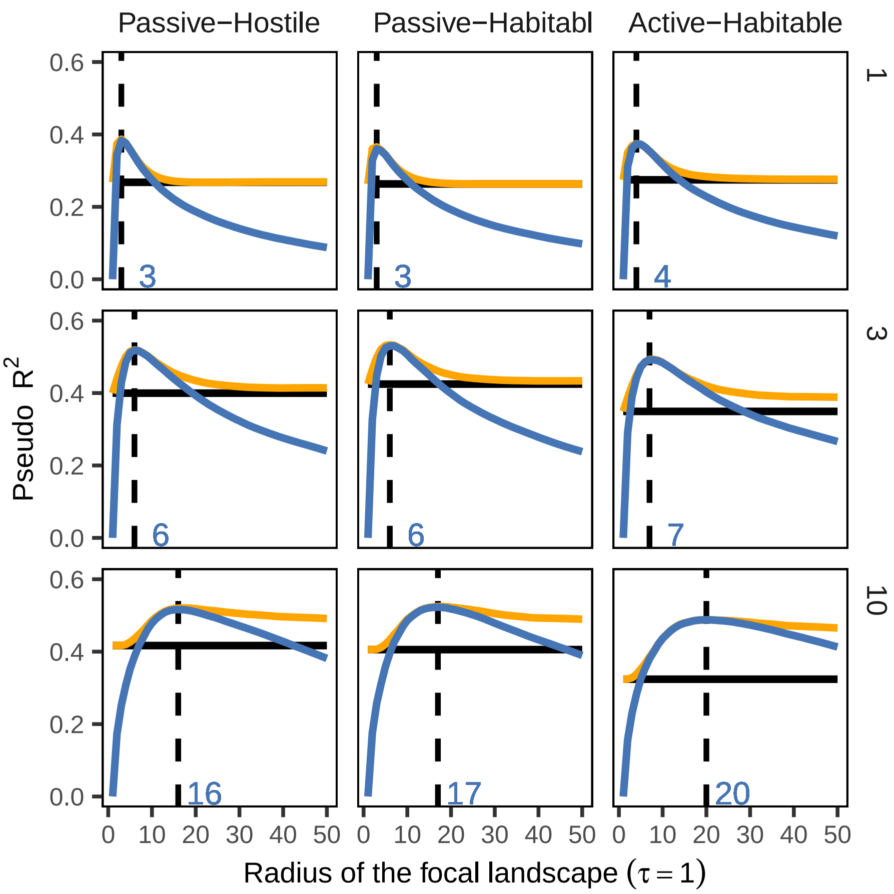

Figure S25: Inferring the appropriate scale of local landscape and testing the sample area effect on a neutral community of size 32. The radius of the sample site is $\tau = 1$.

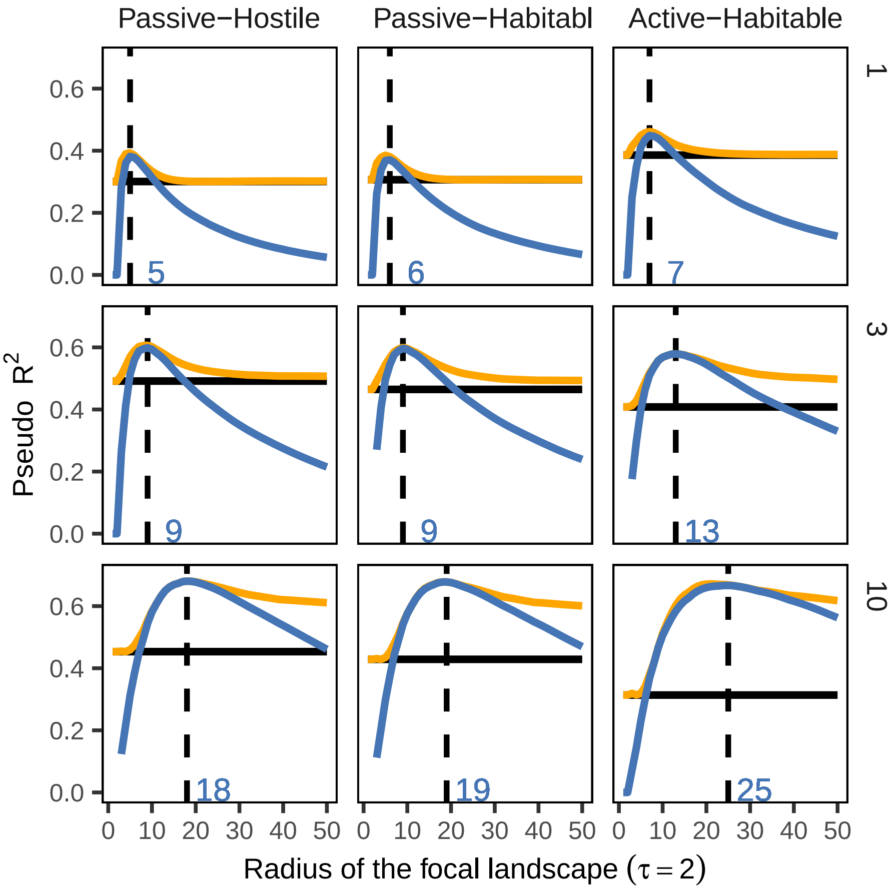

Figure S26: Inferring the appropriate scale of local landscape and testing the sample area effect on a community of size 32. The radius of the sample site is $\tau = 2$.

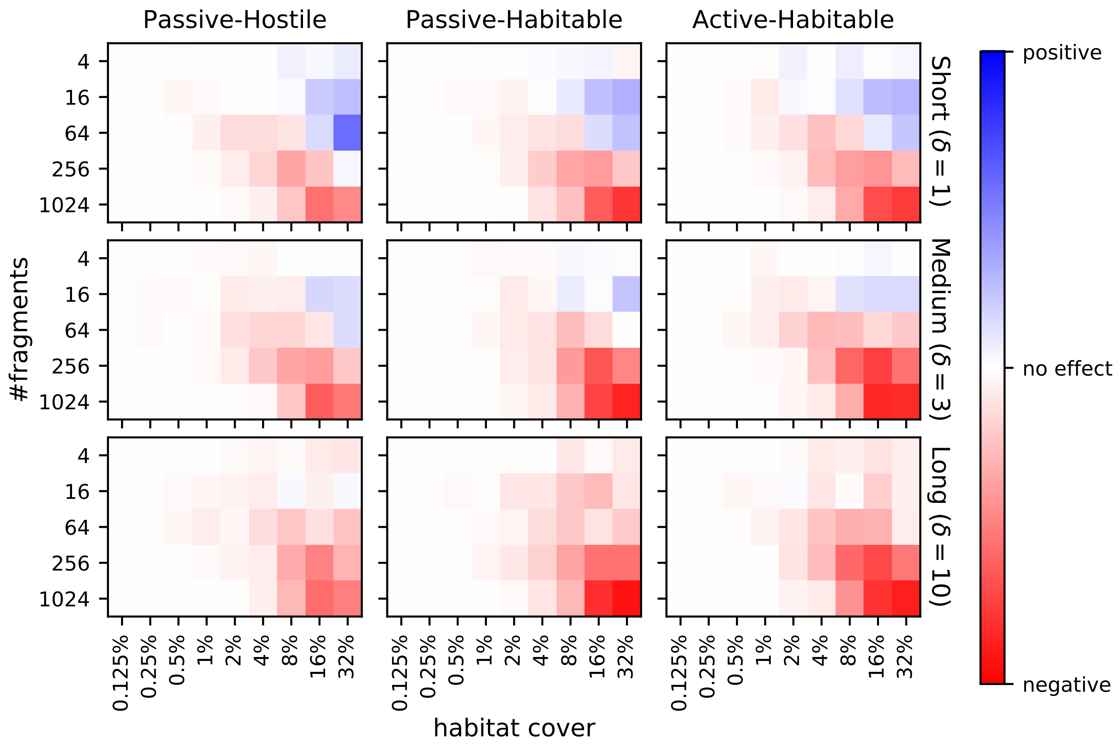

Figure S27: SLOSS analysis of a neutral community of $S = 64$ species in a 25×25 landscape.

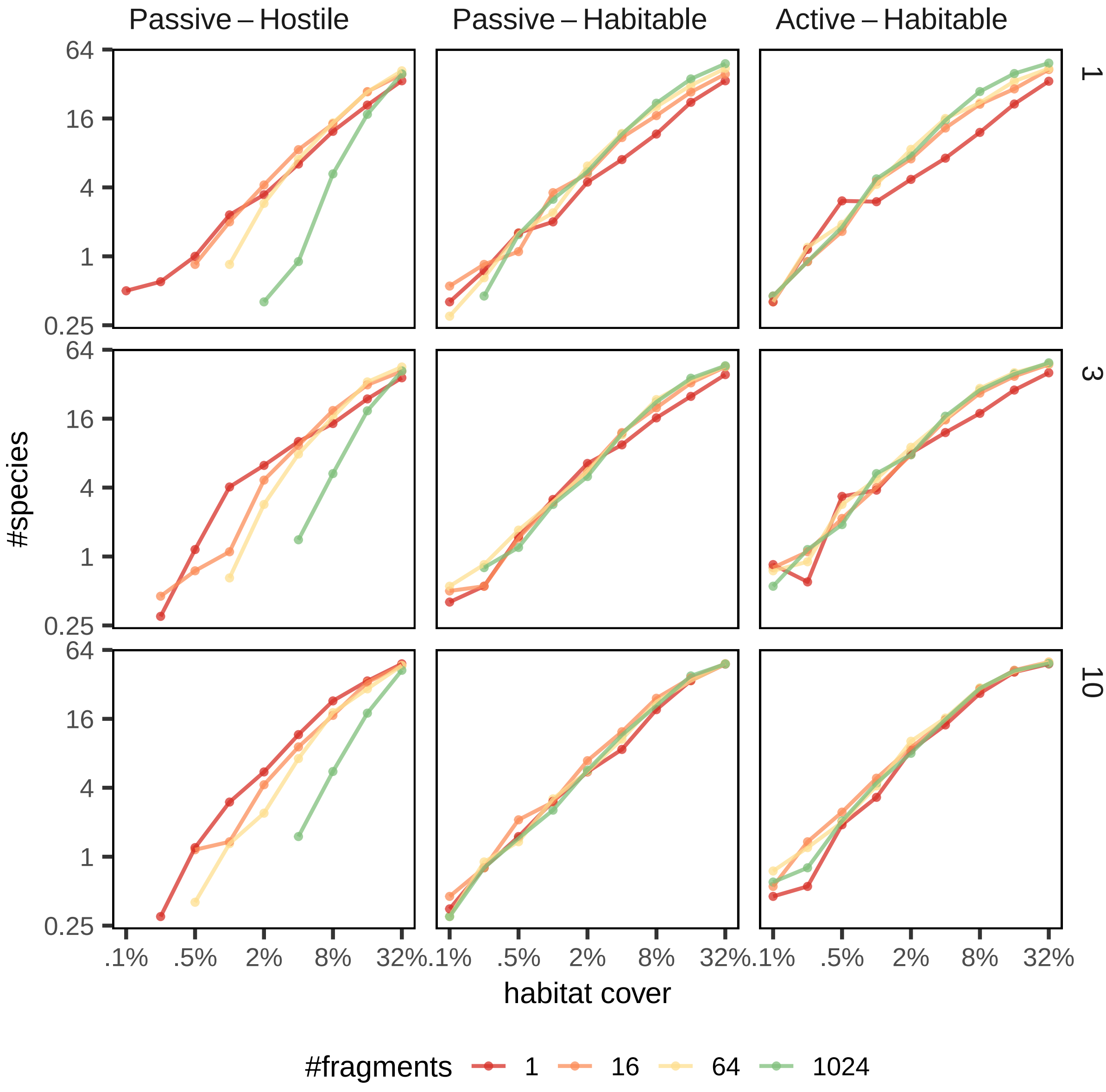

Figure S28: SFAR analysis of of a neutral community of $S = 64$ species in a 25×25 landscape.

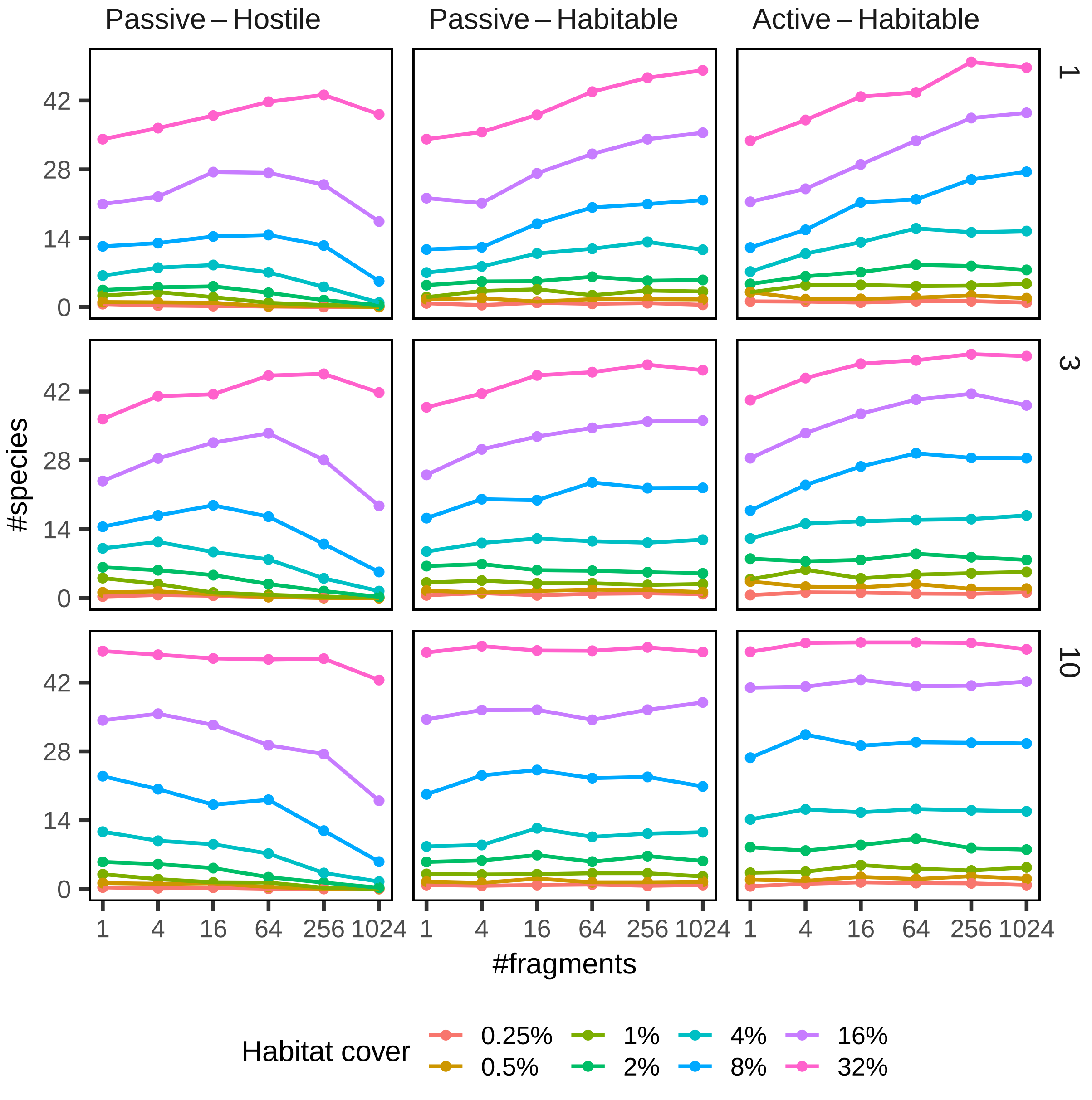

Figure S29: Species richness as a function of habitat fragmentation in a neutral community of $S = 64$ species in a 25×25 landscape.

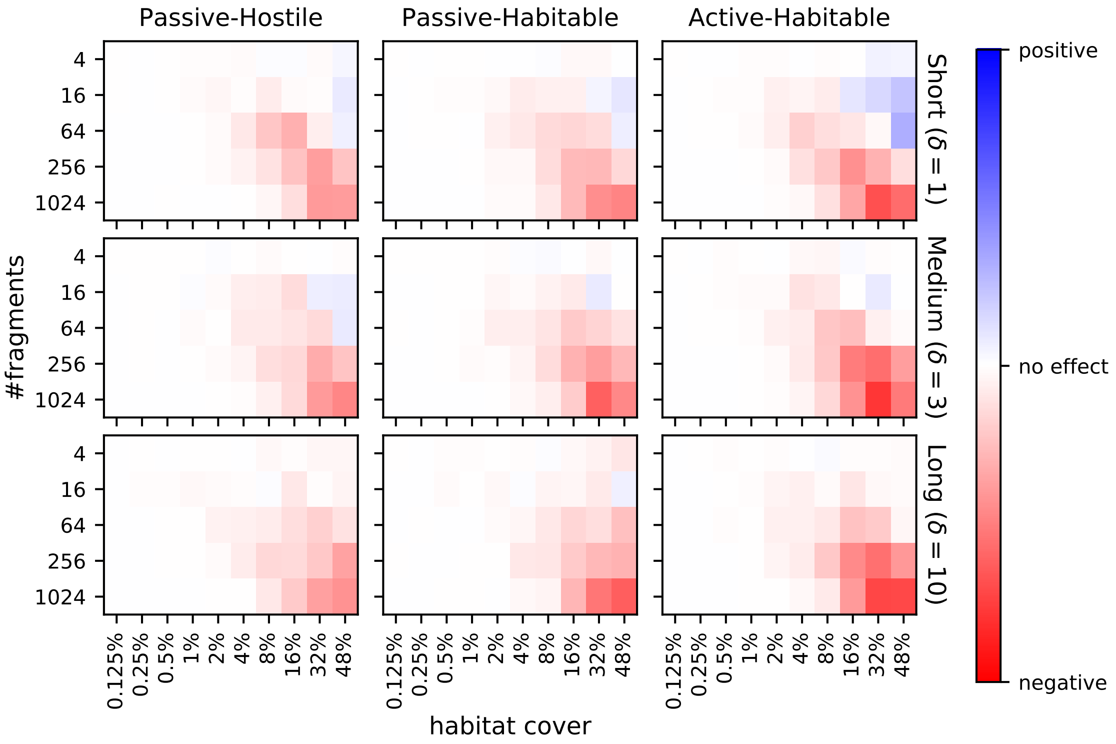

Figure S30: SLOSS analysis of a neutral community of $S = 64$ species in a 25×25 landscape.

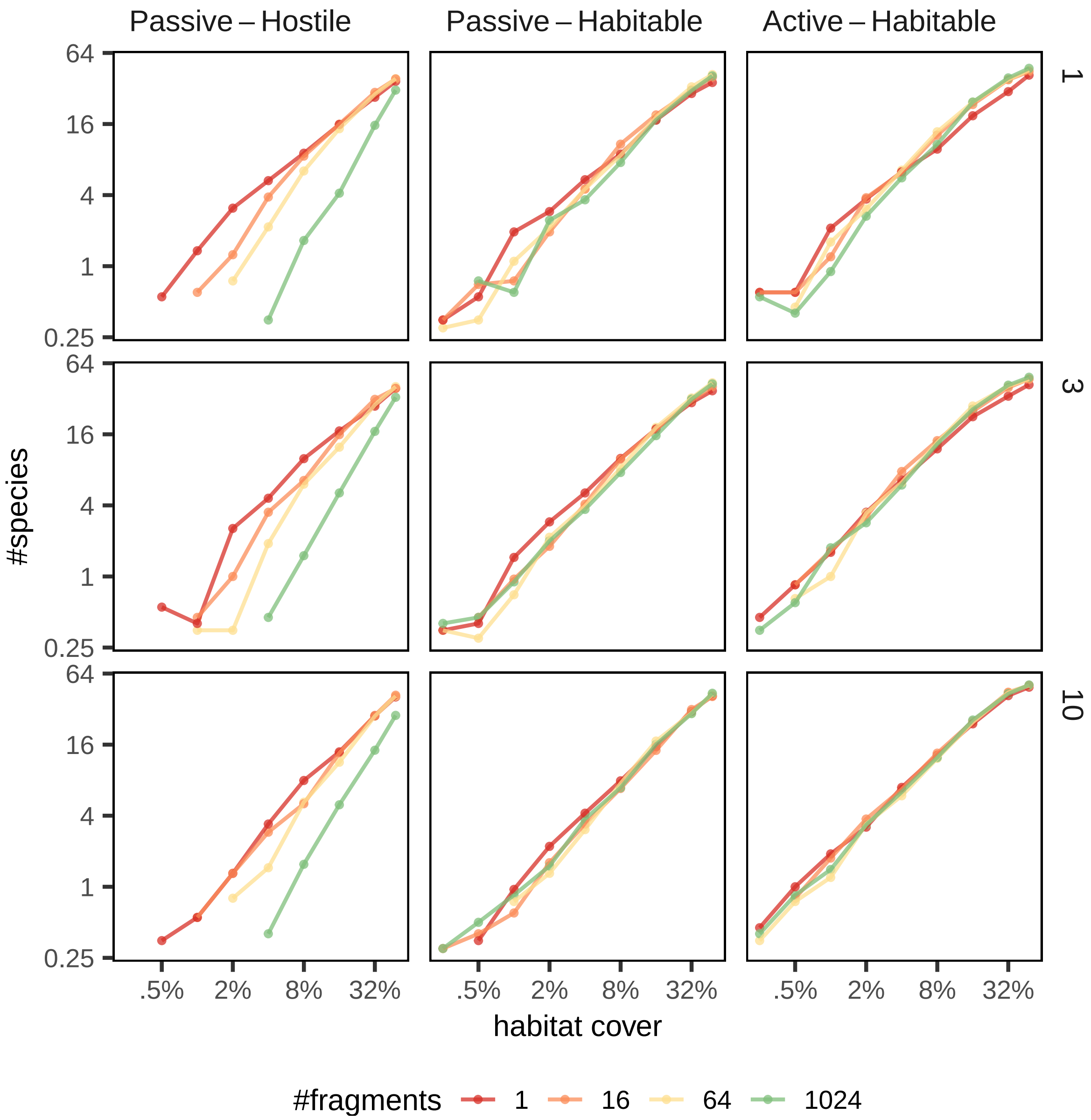

Figure S31: SFAR analysis of of a neutral community of $S = 64$ species in a 25×25 landscape.

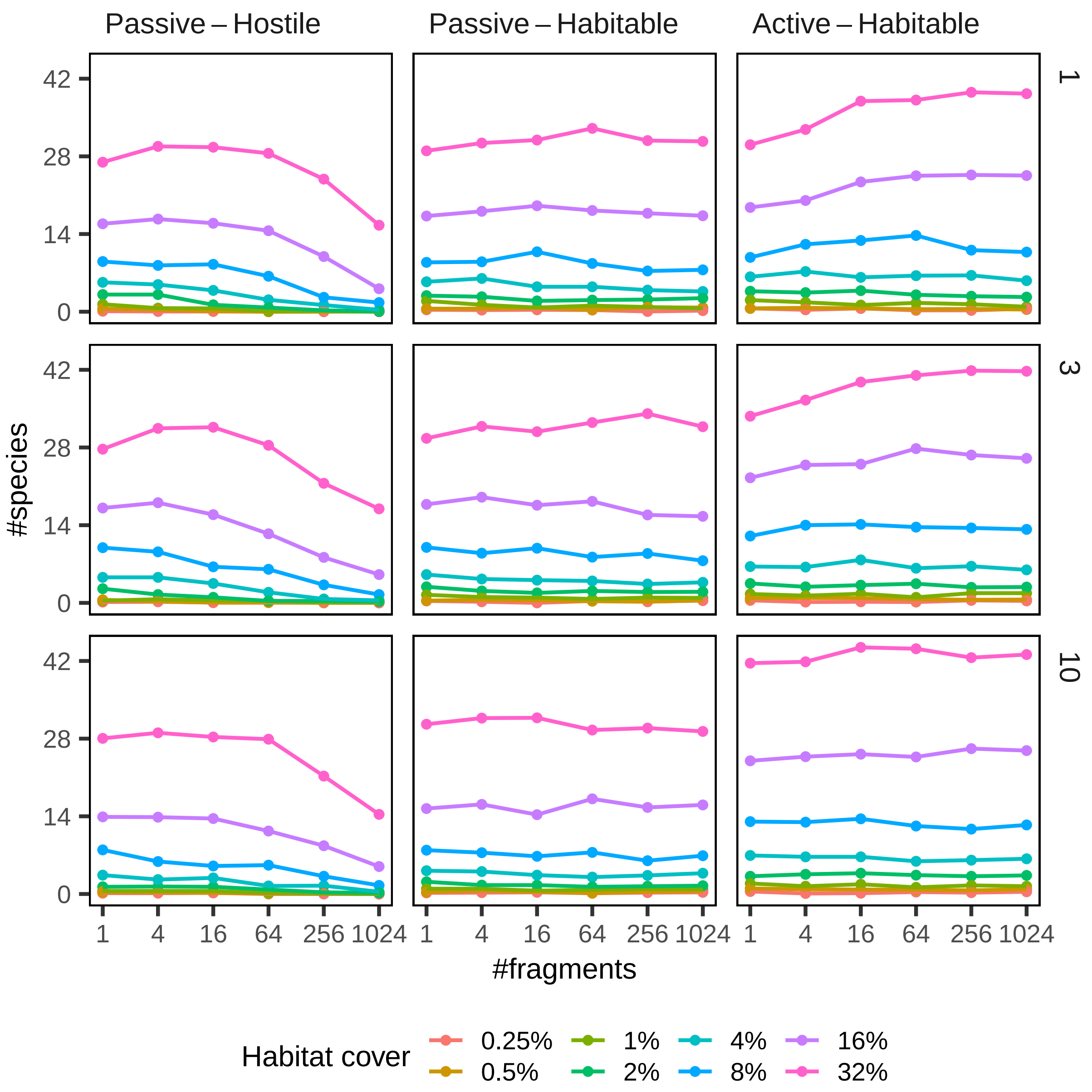

Figure S32: Species richness as a function of habitat fragmentation in a neutral community of $S = 64$ species in a 25×25 landscape.

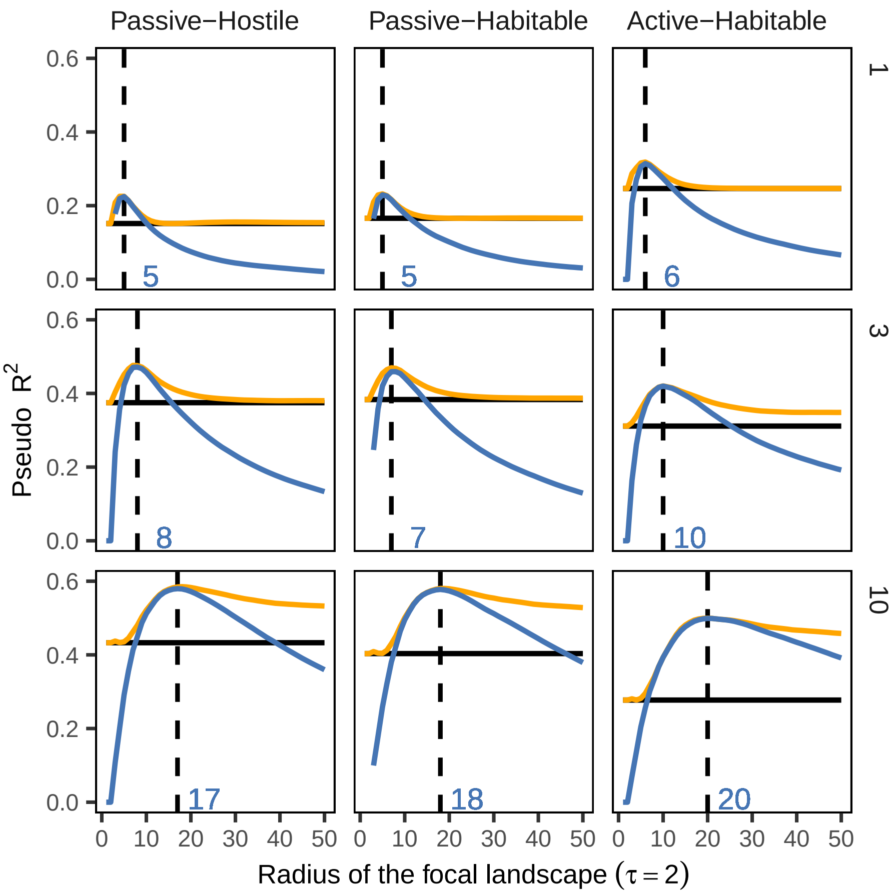

Figure S33: Inferring the appropriate scale of local landscape and testing the sample area effect. This corresponds to the experiment given in the main text, but the radius of the sample site is $\tau = 2$.

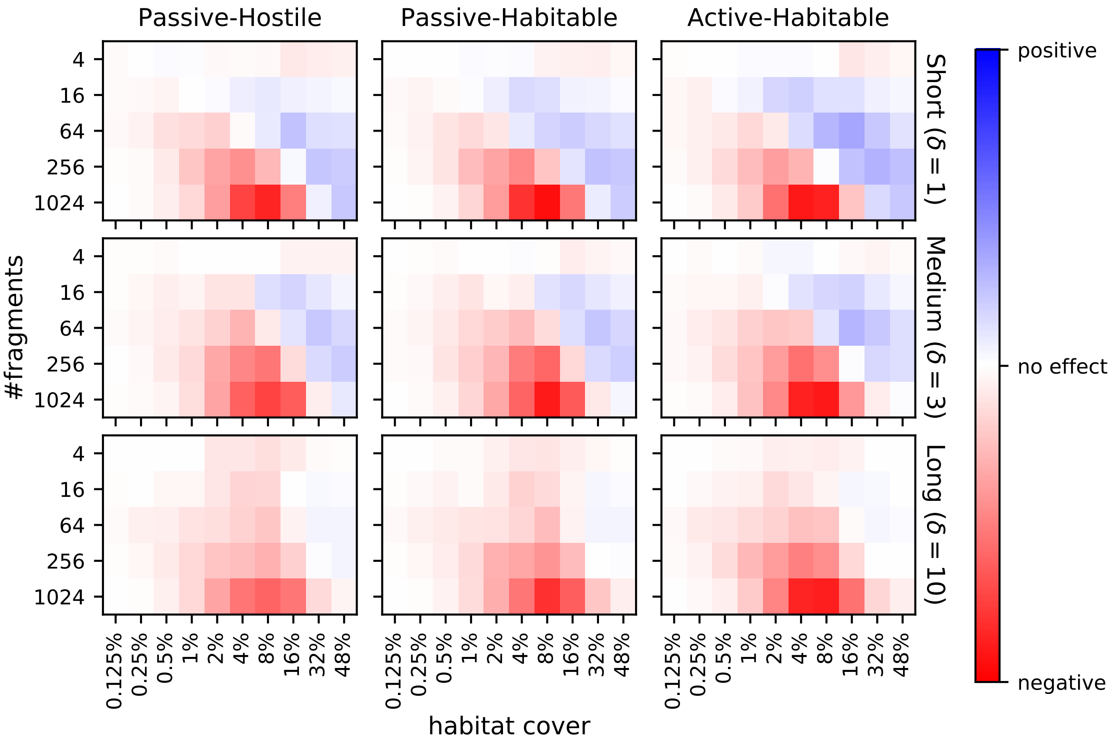

Figure S34: Summary of the SLOSS analysis under the alternative parameterisation (Appendix C.6).

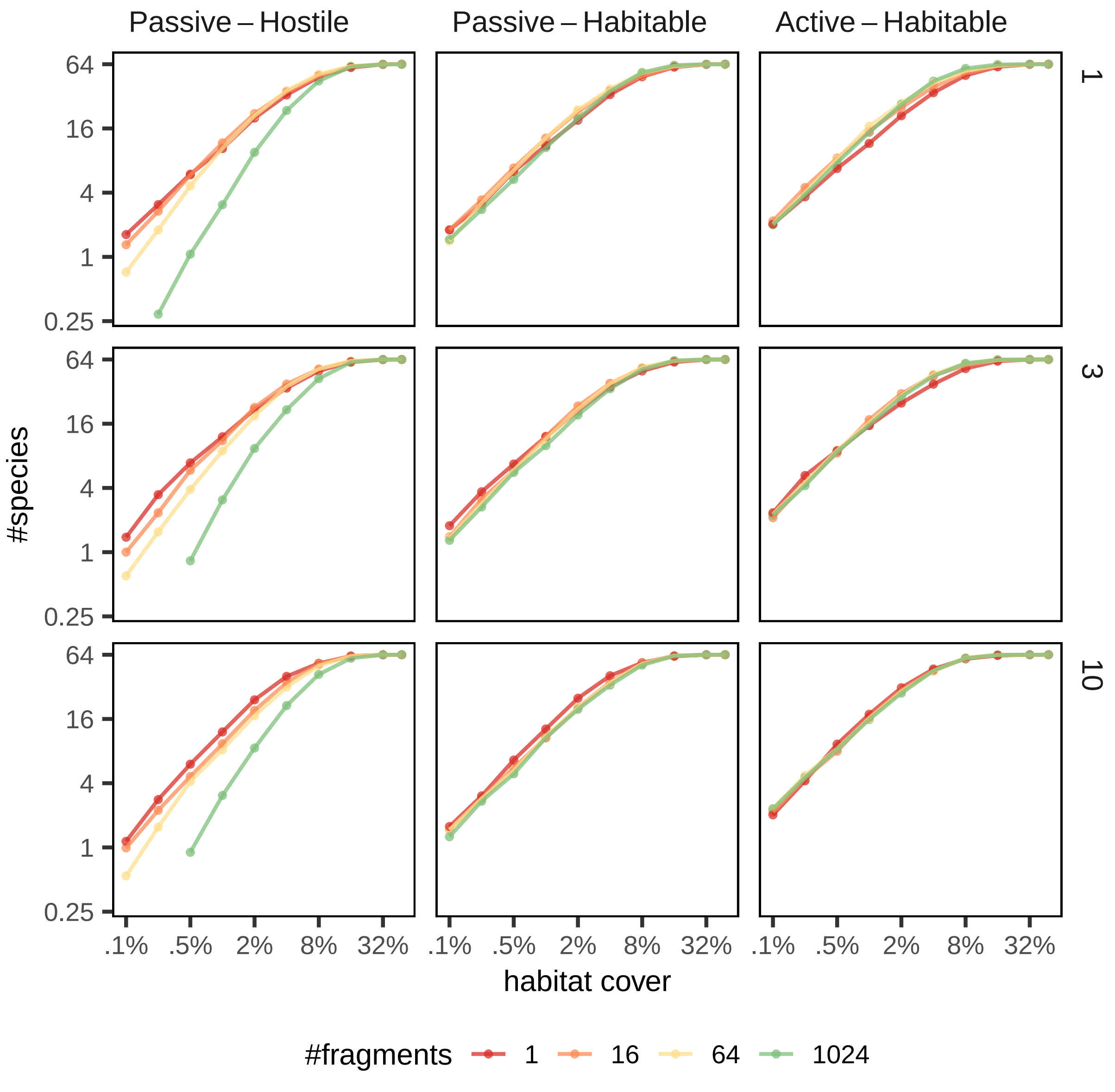

Figure S35: Summary of the SFAR analysis under the alternative parameterisation (Appendix C.6).

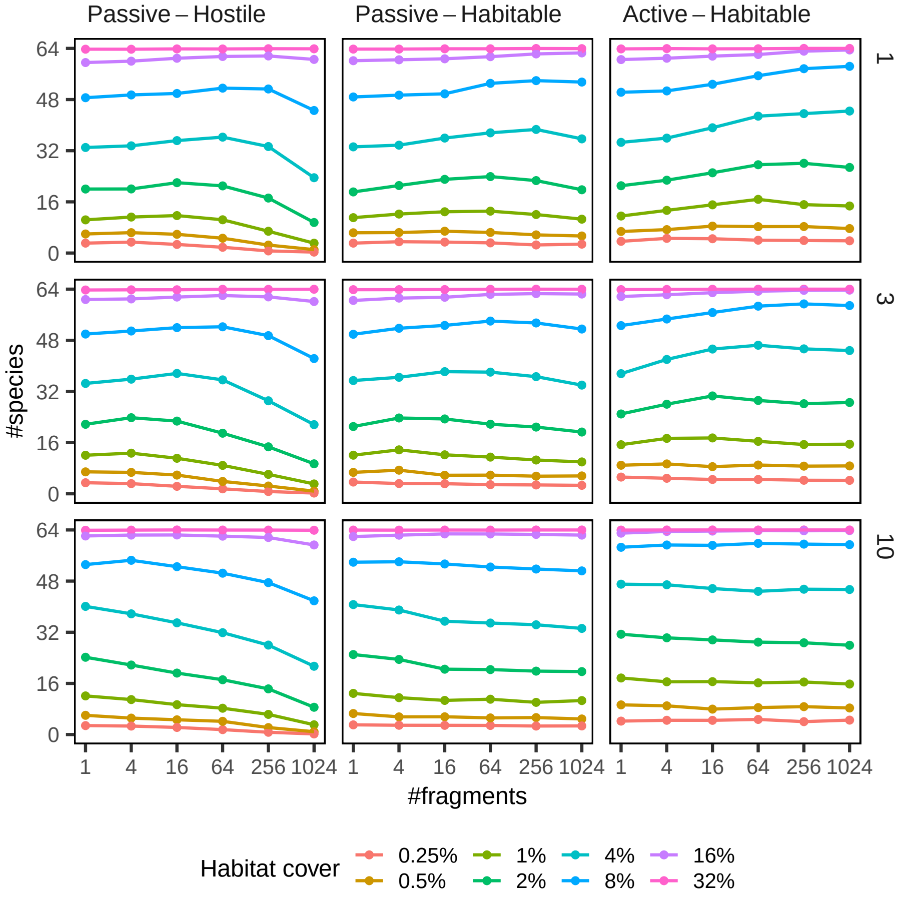

Figure S36: Species richness as a function of habitat fragmentation under the alternative parameterisation (Appendix C.6).

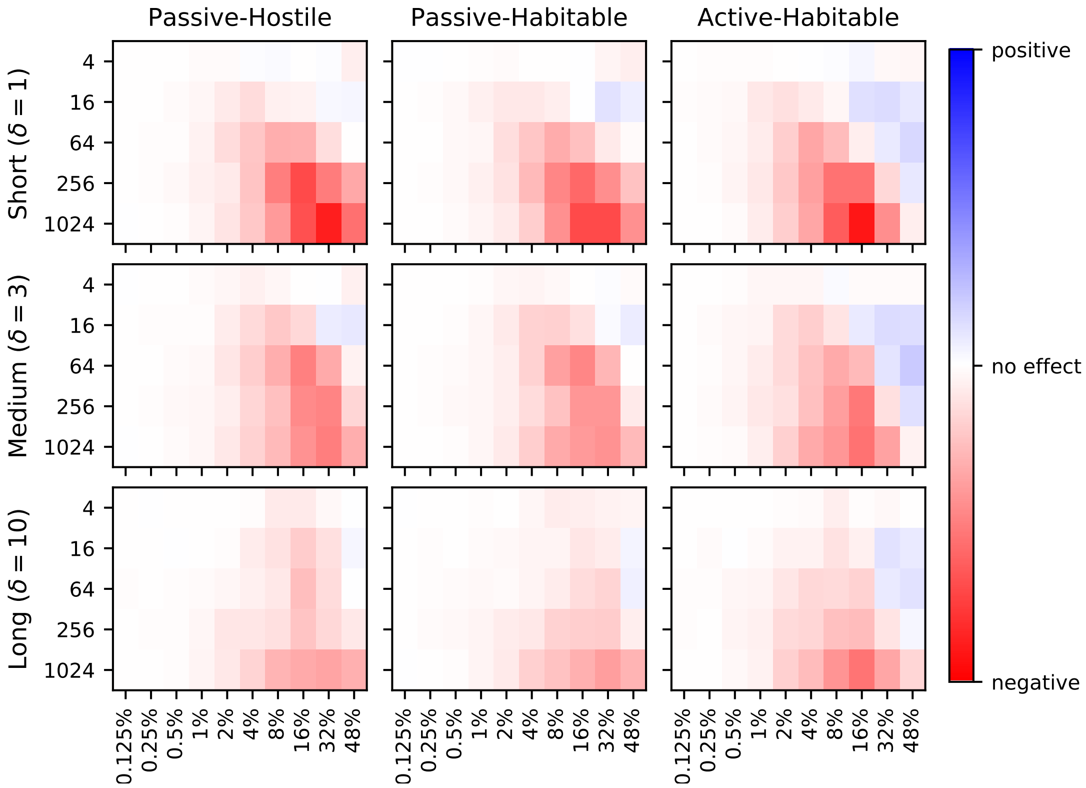

Figure S37: Summary of the SLOSS analysis under the alternative parameterisation (Appendix C.6) in landscapes with environmental gradients (see Fig. 2b in the main text for figure explanation).

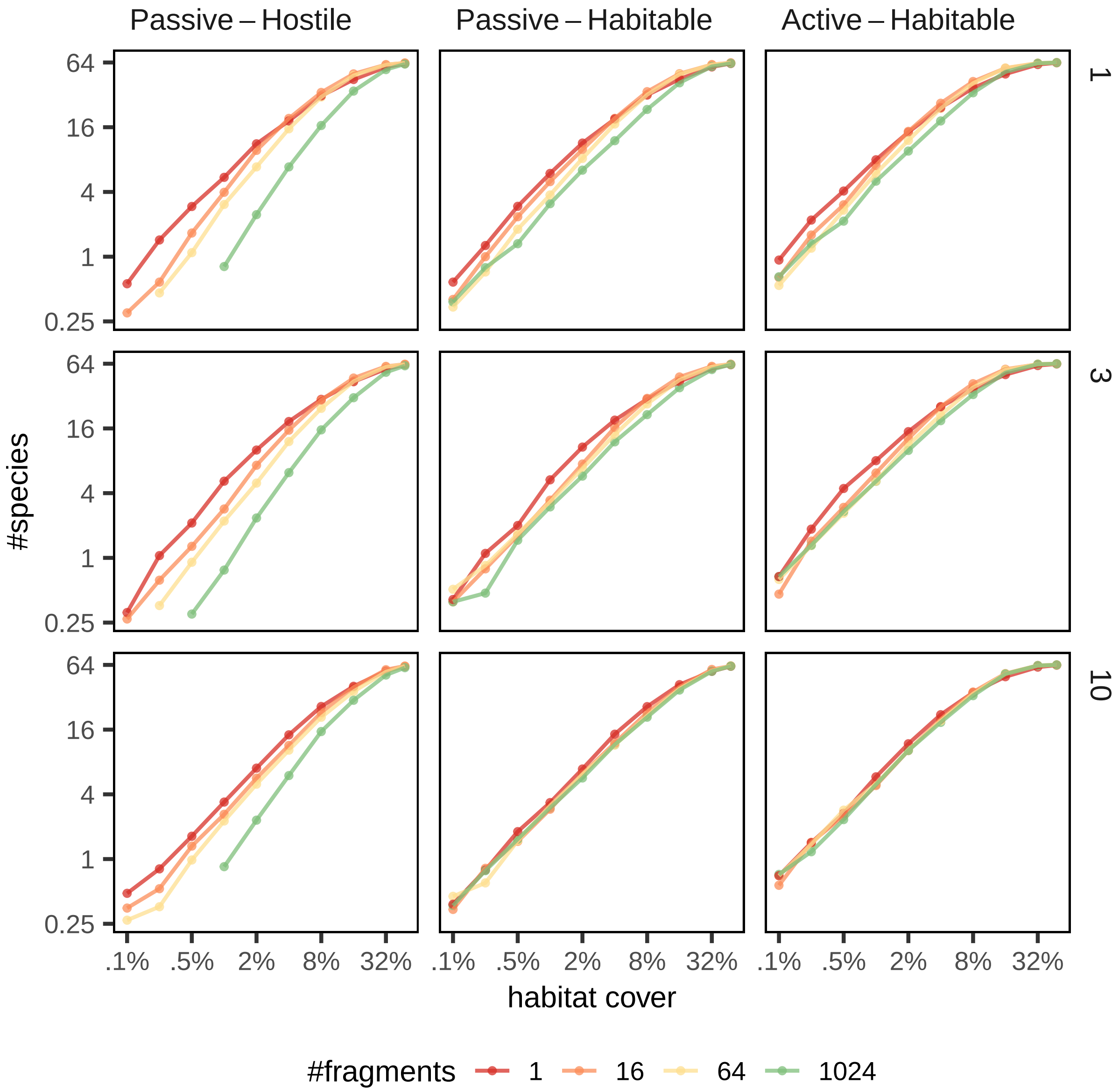

Figure S38: The SFAR analysis under the alternative parameterisation (Appendix C.6) in landscapes with environmental gradients (see Fig. 3 in the main text for figure explanation).

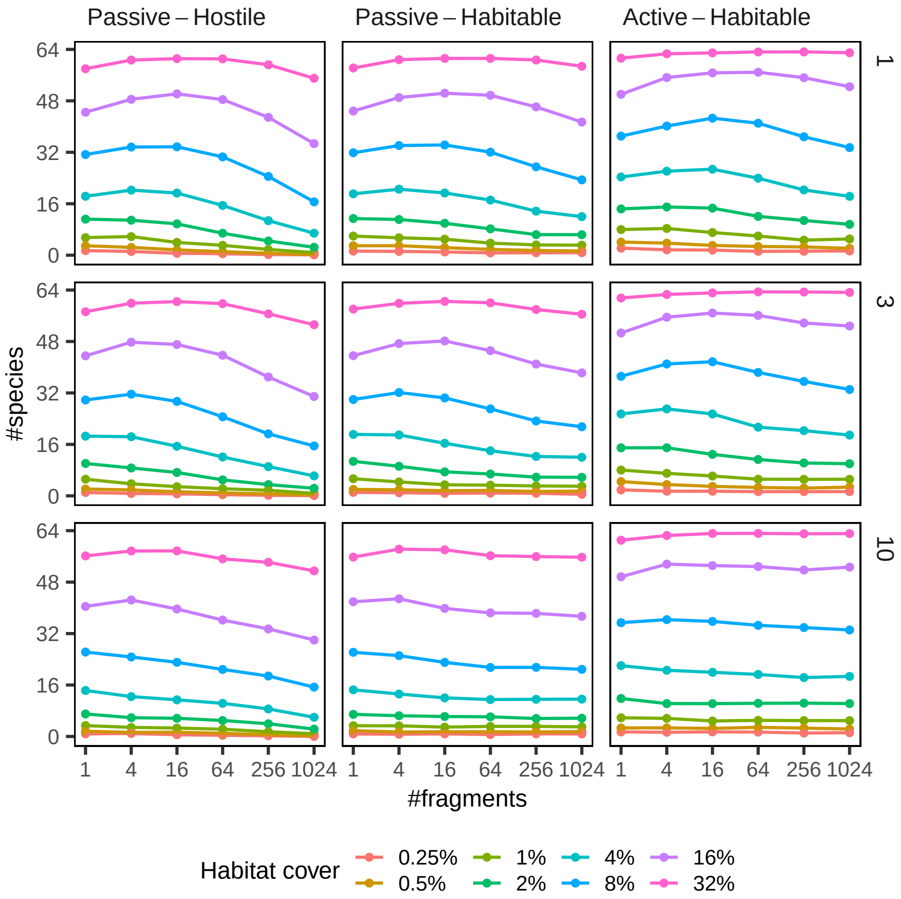

Figure S39: Species richness as a function of habitat fragmentation under the alternative parameterisation (Appendix C.6) in landscapes with environmental gradients (see Fig 4 in the main text for figure explanation).

References

Ilkka Hanski. *Metapopulation Ecology*. Oxford University Press, 1999.

M. A. Leibold, M. Holyoak, N. Mouquet, P. Amarasekare, J. M. Chase, M. F. Hoopes, R. D. Holt, J. B. Shurin, R. Law, D. Tilman, M. Loreau, and A. Gonzalez. The metacommunity concept: A framework for multi-scale community ecology. *Ecology Letters*, 7(7):601–613, 2004. doi:10.1111/j.1461-0248.2004.00608.x.

David Tilman, Robert M. May, Clarence L. Lehman, and Martin A. Nowak. Habitat destruction and the extinction debt. *Nature*, 371(6492):65–66, 1994. doi:10.1038/371065a0.

Michel Loreau, Nicolas Mouquet, and Andrew Gonzalez. Biodiversity as spatial insurance in heterogeneous landscapes. *Proceedings of the National Academy of Sciences of the United States of America*, 100(22):12765–70, 2003. doi:10.1073/pnas.2235465100.

Nicolas Mouquet and Michel Loreau. Community patterns in source-sink metacommunities. *The American Naturalist*, 162(5):544–557, 2003. doi:10.1086/378857.

Omri Allouche and Ronen Kadmon. A general framework for neutral models of community dynamics. *Ecology Letters*, 12(12):1287–1297, 2009. doi:10.1111/j.1461-0248.2009.01379.x.

Shaopeng Wang and Michel Loreau. Biodiversity and ecosystem stability across scales in metacommunities. *Ecology Letters*, 19(5):510–518, 2016. doi:10.1111/ele.12582.

Ricard V. Solé, David Alonso, and Joan Saldaña. Habitat fragmentation and biodiversity collapse in neutral communities. *Ecological Complexity*, 1(1):65 – 75, 2004. ISSN 1476-945X. doi:10.1016/j.ecocom.2003.12.003.

Joel Rybicki and Ilkka Hanski. Species-area relationships and extinctions caused by habitat loss and fragmentation. *Ecology Letters*, 16(SUPPL.1):27–38, 2013. doi:10.1111/ele.12065.

Miguel G. Matias, Dominique Gravel, François Guilhaumon, Philippe Desjardins-Proulx, Michel Loreau, Tamara Münkemüller, and Nicolas Mouquet. Estimates of species extinctions from species-area relationships strongly depend on ecological context. *Ecography*, 37(5):431–442, 2014. doi:10.1111/j.1600-0587.2013.00448.x.

Patrick L. Thompson, Bronwyn Rayfield, and Andrew Gonzalez. Robustness of the spatial insurance effects of biodiversity to habitat loss. *Evolutionary Ecology Research*, 16:445–460, 2014.

Bertrand Fournier, Nicolas Mouquet, Mathew A. Leibold, and Dominique Gravel. An integrative framework of coexistence mechanisms in competitive metacommunities. *Ecography*, (April):1–12, 2016. doi:10.1111/ecog.02137.

Patrick L. Thompson, Bronwyn Rayfield, and Andrew Gonzalez. Loss of habitat and connectivity erodes species diversity, ecosystem functioning, and stability in metacommunity networks. *Ecography*, 40 (1):98–108, 2017. doi:10.1111/ecog.02558.

C.A. Klausmeier. Habitat destruction and extinction in competitive and mutualistic metacommunities. *Ecology Letters*, 4(1):57–63, 2001. doi:10.1046/j.1461-0248.2001.00195.x.

Sona Prakash and André M. de Roos. Habitat destruction in mutualistic metacommunities. *Theoretical Population Biology*, 65(2):153–163, 2004. doi:10.1016/J.TPB.2003.10.004.

Miguel A. Fortuna and Jordi Bascompte. Habitat loss and the structure of plant-animal mutualistic networks. *Ecology Letters*, 9(3):281–286, 2006. doi:10.1111/j.1461-0248.2005.00868.x.

Pradeep Pillai, Michel Loreau, and Andrew Gonzalez. A patch-dynamic framework for food web metacommunities. *Theoretical Ecology*, 3(4):223–237, 2010. doi:10.1007/s12080-009-0065-1.

Clarence L. Lehman and David Tilman. Biodiversity, stability, and productivity in competitive communities. *The American Naturalist*, 156(5):534–552, 2000. doi:10.1086/303402.

Dominique Gravel, Charles D. Canham, Marilou Beaudet, and Christian Messier. Reconciling niche and neutrality: the continuum hypothesis. *Ecology Letters*, 9(4):399–409, 2006. doi:10.1111/j.1461-0248.2006.00884.x.

Jürg B. Logue, Nicolas Mouquet, Hannes Peter, and Helmut Hillebrand. Empirical approaches to metacommunities: a review and comparison with theory. *Trends in Ecology & Evolution*, 26(9): 482–491, 2011. doi:10.1016/J.TREE.2011.04.009.

Bart Haegeman and Michel Loreau. General relationships between consumer dispersal, resource dispersal and metacommunity diversity. *Ecology Letters*, 17(2):175–184, 2014. doi:10.1111/ele.12214.

Dominique Gravel, François Massol, and Mathew A. Leibold. Stability and complexity in model meta-ecosystems. *Nature Communications*, 7(10):12457, 2016. doi:10.1038/ncomms12457.

David Tilman, Clarence L. Lehman, and Chengjun Yin. Habitat destruction, dispersal, and deterministic extinction in competitive communities. *The American Naturalist*, 149(3):407–435, 1997.

Zhichao Xu, Yang Shen, and Jinbao Liao. Patch dynamics of various plant-animal interactions in fragmented landscapes. *Ecological Modelling*, 368:27–32, 2018. doi:10.1016/J.ECOLMODEL.2017.11.017.

Otso Ovaskainen, Dmitri Finkelshtein, Oleksandr Kutoviy, Stephen Cornell, Benjamin Bolker, and Yuri Kondratiev. A general mathematical framework for the analysis of spatiotemporal point processes. *Theoretical Ecology*, 7(1):101–113, 2014. doi:10.1007/s12080-013-0202-8.

Stephen P. Hubbell. *The Unified Neutral Theory of Biodiversity and Biogeography*. Princeton University Press, 2001. ISBN 9780691021287.

Michael A Gibson and Jehoshua Bruck. Efficient exact stochastic simulation of chemical systems with many species and many channels. *The journal of physical chemistry A*, 104(9):1876–1889, 2000. doi:10.1021/jp993732q.

Daniel T Gillespie. A general method for numerically simulating the stochastic time evolution of coupled chemical reactions. *Journal of computational physics*, 22(4):403–434, 1976. doi:10.1016/0021-9991(76)90041-3.

Stephen J. Cornell, Yevhen F. Suprunenko, Dmitri Finkelshtein, Panu Somervuo, and Otso Ovaskainen. A unified framework for analysis of individual-based models in ecology and beyond. *Nature Communications*, 10(4716), 2019. doi:10.1038/s41467-019-12172-y.

Joel Rybicki, Nerea Abrego, and Otso Ovaskainen. Online supplementary material, 2019. `https://bitbucket.org/jrybicki/habitat-fragmentation-and-species-diversity`.
